# 5-oxoETE links redox control of epithelial damage detection and resilience

**DOI:** 10.1038/s41586-026-11121-2

**Published:** 2026-10-07

**Authors:** Yanan Ma, Miklós Lengyel, King Lam Hui, Yohannes A. Ambaw, Zaza Gelashvili, Leehyeon Kim, Ritchie Ly, Siyang Peng, Meysoon Quraishi, Tobias C. Walther, Robert V. Farese, Philipp Niethammer

**Affiliations:** 1 Cell Biology Program, Memorial Sloan Kettering Cancer Center, New York, NY, USA.; 2 Gerstner Sloan Kettering Graduate School of Biomedical Sciences, New York, NY, USA.; 3 Department of Pharmacology, Weill Cornell Medical College, New York, NY, USA.; 4 Howard Hughes Medical Institute, New York, NY, USA.; 5 These authors contributed equally: Yanan Ma, Miklós Lengyel.

## Abstract

Organisms harness oxidative stress to rapidly attract white blood cells to wound sites and to kill pathogens^1–3^. To this end, host tissues increase their own oxidative stress resilience and repair capacity via adaptive redox signalling^4–6^. Here, using live zebrafish and human cells, we identify a metabolic redox signalling mechanism that integrates oxidative immune defence with tissue adaptation. We demonstrate that DHRS7, an orphan short-chain fatty acid dehydrogenase–reductase, generates or consumes the pro-inflammatory lipid 5-oxoETE as a function of cytoplasmic NADP^+^/NADPH ratio. At wounds, where oxidative stress and NADP^+^ are high, 5-oxo-eicosatetraenoic acid (5-oxoETE) production by DHRS7 rapidly alerts antimicrobial white blood cells through the G-protein-coupled receptor OXER1. In undamaged tissue, where NADP^+^ is low, DHRS7 quenches unnecessary inflammation. Notably, we find that 5-oxoETE also supports epithelial redox resilience; OXER1-deficient zebrafish exhibit intestinal apoptosis, barrier disruption and microbial inflammation. Mechanistically, 5-oxoETE induces the expression of NUDIX hydrolases, which protect the cytoplasmic nucleotide pool from oxidation and prevent apoptosis in zebrafish and human intestinal cells. Thus, our data reveal a conserved mode of redox sensing and signalling—beyond classic thiol oxidation—that leverages NADPH metabolism to orchestrate the antimicrobial and pro-resilience functions of oxidative stress.

Oxidative stress is a hallmark of inflammatory, cardiovascular, fibrotic, infectious and neurological diseases^1^. Although in excess it can cause toxic damage to biomolecules, oxidative stress also serves fundamental innate immune functions. For example, NADPH oxidases (NOX proteins) of integumental surfaces and white blood cells produce large amounts of reactive oxygen species (ROS) to kill intruding microbes. Oxidative stress also marks potential sites of pathogen entry, such as wounds, to bring in immune reinforcements^2,3^. Concomitantly, non-lethal oxidative stress confers host resilience against ROS-induced bystander damage by upregulating antioxidant circuits or triggering regenerative responses^4,5^. The latter may include the polarization or recruitment of repair leukocytes (N2 neutrophils or M2 macrophages), stimulation of growth factor signalling and upregulation of antioxidant defences (for example, glutathione peroxidases)^5^. To serve as diffusible immune and resilience signals, ROS can reversibly modify cysteines of signalling proteins^6^. In addition, oxidative stress may trigger redox-mediated metabolic signals. For instance, at zebrafish wounds, NOX-mediated ROS generation locally alters the NADP^+^/NADPH cofactor balance in vivo^7^. Animals and humans that lack epithelial or myeloid NOX activity are prone to infection, for instance, during chronic granulomatous disease, a hereditary NOX2 deficiency^8^. The NOX-deficiency phenotypes cannot be solely explained by a lack of microbicidal ROS function and seem to involve aberrant redox signalling. Most current research on redox signalling focuses on the posttranslational modification of kinases and phosphatases by reversible thiol oxidation, whereas the contribution of ‘metabolic’ redox cues remains largely unexplored.

Zebrafish is a powerful animal model for the study of redox biology and rapid wound responses by intravital imaging. Their innate and adaptive immune repertoire and their responses to infection or wounds largely mirror those of mammals^9^. In zebrafish larvae, Ca^2+^ influx at the wound margin activates the epithelial NOX Duox. This enzyme produces H_2_O_2_ while consuming NADPH to mediate rapid leukocyte recruitment^2^. Acute neutrophilic wound inflammation via Duox and H_2_O_2_ was shown to involve both reversible Lyn kinase oxidation and production of 5-oxoETE^3,10,11^. 5-oxoETE serves as a potent leukocyte chemoattractant in fish and primates and is linked to allergic inflammation, infection defence, colitis and possibly cancer^11–13^. During oxidative stress, 5-oxoETE is formed from arachidonic acid by non-enzymatic lipid peroxidation or by 5-lipoxygenase (ALOX5) and NADP^+^-dependent 5-hydroxyeicosanoid dehydrogenase (5-HEDH) activity^14^. Research into the oxoeicosanoid pathway has been hampered by the unknown genetic identity of 5-HEDH and the lack of obvious OXER1 orthologues in rodents. Here we set out to identify the 5-HEDH enzyme to better understand its regulation by oxidative stress and its physiological function.

## Identification and characterization of 5-HEDH as DHRS7

Earlier work^14^ characterized 5-HEDH as a microsomal lipid dehydrogenase–reductase that interconverts 5(*S*)-HETE and 5-oxoETE in an NADP^+^/NADPH-dependent manner^15^ (Fig. 1a and Extended Data Fig. 1a), without identifying its genetic identity. We confirmed reversible 5-HEDH activity in human lung and intestinal epithelial cancer cells (A549 and Caco-2) using liquid chromatography–mass spectrometry (LC–MS) to detect product formation after supplying 5(*S*)-HETE or 5-oxoETE as substrates (Fig. 1b and Extended Data Fig. 1b). Consistent with a low cytoplasmic NADP^+^/NADPH ratio in unstressed cells, there was a pronounced bias towards 5-oxoETE consumption. Based on the known cofactor dependence, subcellular localization^16^ and evolutionary conservation of 5-HEDH, we compiled a high-priority shortlist of 5-HEDH candidates and tested it using short hairpin RNA (shRNA) knockdown in A549 cells. Only knockdown of *DHRS7*, which encodes a short-chain dehydrogenase–reductase (SDR)^17^, significantly reduced 5-oxoETE or 5-HETE below wild-type control levels (Extended Data Fig. 1c,d). Although knockdown of *LMNA*, *HSD17B2*, *PECR* and *CBR1* also reduced 5-HETE levels, the effects were less pronounced than those observed with DHRS7 knockdown, and none of these knockdowns decreased 5-oxoETE levels (Extended Data Fig. 1c,d).

The DHRS7 nucleotide and protein sequences are conserved from fish to humans (Extended Data Fig. 2a,b). Zebrafish and human DHRS7 proteins are approximately 58% identical. Although there are no available experimental DHRS7 structures, the superposition of AlphaFold predictions for DHRS7 orthologues points to high structural conservation (Extended Data Fig. 2c). DHRS7 features a NADP(H)-binding Rossman fold, and localizes to intracellular membranes, including those of the endoplasmic reticulum^18,19^, nucleus and mitochondria (Extended Data Fig. 2d–f). Based on co-localization metrics (Pearson’s *r*, Manders’ M1 and Manders’ M2), DHRS7 overlaps stronger with endoplasmic reticulum and nuclear markers than with mitochondrial markers (Extended Data Fig. 2f). Its cofactor preference and localization (endoplasmic reticulum)^16^ are consistent with those reported for 5-HEDH. Whereas DHRS7 was initially proposed to face the endoplasmic reticulum lumen, subsequent studies demonstrated its cytoplasmic orientation^20,21^ (Extended Data Fig. 2d). DHRS7 is broadly expressed in immune cells (for example, macrophages) and non-immune cells (for example, epithelial and glandular cells and fibroblasts) in various tissues including the prostate, liver and digestive tract^19^. Zebrafish single-cell RNA sequencing resources suggest that the overall expression pattern is relatively conserved^22^. In vitro, DHRS7 possesses reductase activity towards dihydrotestosterone, benzil and 4,4′-dimethylbenzil^23^. More recently, DHRS7 was identified as an acyl–alkyl-glycerone phosphate reductase that generates a distinct subset plasmalogen species compared with DHRS7B^24^. However, its physiological substrates and broader biological functions remain largely unknown. Of note, dehydrogenase activity was not previously described for DHRS7. Furthermore, the previously described conversion of 5α-dihydroxytestosterone to 3α-androstanediol also reflects reductase rather than dehydrogenase activity^21^.

To independently confirm our shRNA knockdown results, we generated DHRS7-knockout A549 cell lines using two different single guide RNAs (sgRNA1 and sgRNA2) (Extended Data Fig. 1e). 5-oxoETE and 5-HETE synthesis was significantly reduced in three independent DHRS7-knockout clones compared with the wild type (Fig. 1c). Similar results were obtained in CRISPR–Cas9-mediated DHRS7-knockdown Caco-2 cells (Extended Data Fig. 1f). In line with the previously reported endogenous subcellular localization, doxycycline (Dox)-induced DHRS7–eGFP localized to endomembranes (Fig. 1d and Extended Data Fig. 2d–f) and rescued 5-oxoETE and 5-HETE production (Fig. 1e). Dox alone (that is, without DHRS7 induction) had no such effect (Fig. 1f). DHRS7 overexpression also increased 5-oxoETE production of Caco-2 cells (Extended Data Fig. 1g). Overall, our data show that DHRS7 reversibly mediates the conversion of 5(*S*)-HETE to 5-oxoETE. In both unperturbed lung- and intestinal-derived epithelial cells, the reaction is biased towards the reverse (reductase) direction, suggesting that DHRS7 functions as 5-oxoETE sink at baseline.

## Lipid peroxidation stimulates 5-oxoETE via DHRS7

To delineate whether or how oxidative stress affects 5-oxoETE production (Fig. 1g–i and Extended Data Fig. 1h), we exposed A549 DHRS7-knockout cells with or without Dox-induced DHRS7 expression overnight to RSL3 (Fig. 1g), which facilitates lipid peroxidation by inhibiting GPX4, or imidazole ketone erastin (IKE), which impairs cellular cystine import and glutathione (GSH) production. Alternatively, cells were briefly subjected to H_2_O_2_, which—unlike RSL3 and IKE—can directly initiate lipid peroxidation in the presence of iron^25^. H_2_O_2_ also increases the NADP^+^/NADPH ratio (Extended Data Fig. 3a), most probably because the rapid oxidation of GSH to glutathione disulfide (GSSG) drives NADPH consumption through glutathione reductase and other NADPH-dependent antioxidant systems. This compensatory response helps to protect cells against irreversible lipid and protein oxidation.

Pointing to additional enzymatic or non-enzymatic production routes, H_2_O_2_ exposure of cells increased 5-oxoETE levels in the absence of DHRS7 (Fig. 1h and Extended Data Fig. 1h, left). In the presence of DHRS7, these levels were substantially increased (Fig. 1h and Extended Data Fig. 1h, right). Hydrophilic (Trolox) and lipophilic (liproxstatin-1 (LPX1)) ROS quenchers completely abrogated H_2_O_2_-dependent enzymatic and non-enzymatic 5-oxoETE production, presumably by buffering oxidative stress (that is, limiting NADPH consumption by antioxidant circuits) and scavenging reactive oxygen intermediates that are involved in non-enzymatic 5-oxoETE formation. RSL3 and IKE significantly stimulated 5-oxoETE production in the presence of DHRS7 or after H_2_O_2_ pretreatment (Extended Data Fig. 1h), suggesting that DHRS7 sensitizes 5-oxoETE production to oxidative stress. Lipid peroxidation quenchers (LPX1 and vitamin E) curb rapid neutrophil recruitment to larval zebrafish wounds in vivo^26^. At least in part, this anti-inflammatory effect might derive from inhibition of 5-oxoETE production. Together, these regulatory relationships (Fig. 1i) suggest that DHRS7 mediates inflammatory redox sensing.

## Conserved 5-HEDH activity of DHRS7

5-HEDH activity was often studied in microsome suspensions, where redox cofactor concentrations can be freely adjusted, and the reaction monitored via an NADPH fluorescence readout^27^. As mentioned above, previous studies suggest that the DHRS7 faces the cytosol^20,21^ (Extended Data Fig. 2d)—that is, the enzyme is exposed to the cytoplasmic cofactor pool. By western blotting and NADPH fluorescence monitoring, we detected endogenous DHRS7 protein and activity in nuclear, granular (mitochondria and lysosomes), and microsomal fractions of HEK293T cells, but not in the cytosol (Extended Data Fig. 3b,c). To generate DHRS7-deficient microsomes, we depleted DHRS7 in HEK293T cells using CRISPR–Cas9 (Fig. 2a,b). Compared with wild-type microsomes, the DHRS7-deficient microsomes were severely impaired in converting 5(*S*)-HETE to 5-oxoETE and vice versa (Fig. 2c). Overexpression of human DHRS7 (*Hs*DHRS7) or zebrafish Dhrs7 (*Dr*Dhrs7) restored both the forward and reverse reactions (Fig. 2d–g and Extended Data Fig. 3d,e). 5-HEDH activity was retained after purification of the Flag-tagged protein. Compared with the purified *Hs*DHRS7, purified *Dr*Dhrs7 exhibited pronounced dimerization along with lower enzyme activity, possibly caused by sub-optimal purification conditions (Fig. 2h and Extended Data Fig. 3h–m). Collectively, our cell, microsome and purified protein experiments establish the evolutionary conserved 5-HEDH function of DHRS7.

On microsomes, the dehydrogenase reactions of *Hs*DHRS7 and *Dr*Dhrs7 showed similar pseudo-first order kinetics when varying 5(*S*)-HETE or NADP^+^ and measuring NADPH product formation using the same amount of microsomal *Dr*Dhrs7 or *Hs*DHRS7 (Michaelis constant (*K*_m_) ≈ 0.5–1.5 μM, maximal velocity (*V*_max_) ≈ 15–50 nmol min^−1^ pmol^−1^ enzyme; Extended Data Fig. 3f,g). By contrast, the related SDR enzymes DHRS7B, DHRS7C, DHRS9, RDH11 and 15-PGDH^28^ showed no significant 5-HEDH activity (Extended Data Fig. 4a–h), suggesting that 5-HEDH activity is specific for DHRS7. As expected, NAD^+^ was a less efficient cofactor than NADP^+^ (Extended Data Fig. 3f, middle). Besides 5(*S*)-HETE, DHRS7 also oxidized 5(*S*)-HEPE, 6-*trans*-12-*epi* leukotriene B_4_ (LTB4) and 5*S*,15*S*-dihydroxy-6E,8Z,10Z,13E-eicosatetraenoic acid (5*S*,15*S*-DiHETE), but not 5(*R*)-hydroxyeicosatetraenoic acid (HETE), LTB4 or 12*S*-HETE (Extended Data Fig. 4i–n), consistent with previous findings obtained using whole microsomal fractions^15,29^. Thus, the allylic 5(*S*)-hydroxyl group seems to be required and (*R*)-hydroxyls are preclusive for 5-HEDH dehydrogenase activity, in line with previous reports on 5-HEDH. Assuming a cytoplasmic NADP(H) pool size of about 3 μM (ref. 7), the micromolar *K*_m_ of DHRS7 would be expected to function well as a cytoplasmic NADP^+^/NADPH sensor^29,30^.

Informed by previous studies of the catalytic^31^ and cofactor-binding sites^32^ of SDR family proteins, we generated five site-directed mutants of *Hs*DHRS7 (K207E, Y203F, S190A, N162L and R82E). Although none of the mutations altered the subcellular localization of the enzyme, the K207E, Y203F and S190A mutations abrogated DHRS7 activity, whereas the N162L and R82E mutations had a stimulatory effect (Fig. 2i). When microsomes were provided with 5(*S*)-HETE, human, zebrafish and bovine DHRS7 showed similar rates of NADPH generation, but rat and mouse DHRS7 were much less active towards the same substrate (Fig. 2j). Species-dependent differences in the substrate selectivity among highly homologous enzymes or transporters are not uncommon^33^. Despite their conserved catalytic centre and similar secondary structures, SDR enzymes are known to display high catalytic plasticity^34^. Sequence alignment of the human, bovine, zebrafish, mouse and rat DHRS7 protein sequences indicates various amino acids as differentially conserved between the rodent or non-rodent orthologues (Extended Data Fig. 5). Probing their functional significance by systematic mutation may further explain the structural basis of species-specific 5-HEDH.

## Dhrs7 mediates rapid wound detection in live zebrafish

Overall, the above experiments underlined the redox-regulated bidirectionality of the Dhrs7 reaction (Fig. 1 and Extended Data Fig. 6f). In unstressed tissue, where the cellular NADPH/NADP^+^ ratio is high, the reductase direction of the enzyme (5-oxoETE → 5-HETE) is thermodynamically favoured, that is, DHRS7 functions as a metabolic sink for 5-oxoETE at baseline conditions. The removal of this sink from uninjured tissue should give rise to 5-oxoETE accumulation from DHRS7-independent sources. 5-oxoETE accumulation was indicated by our DHRS7-knockout cell line experiments (Fig. 1h, –Dox). Using CRISPR–Cas9, we generated *dhrs7* mutant fish (Extended Data Fig. 6a–e) and subjected their uninjured tail fins to LC–MS lipidomic (LIPID MAPS) analysis. As predicted from the enzymology of DHRS7–5-HEDH, the samples from *dhrs7* mutant adults showed a significantly higher 5-oxoETE baseline along with decreased levels of select prostaglandin-related lipids (for example, bicyclo-prostaglandin E_2_ (bicyclo-PGE_2_)) (Fig. 3a and Supplementary Table 1).

Given the highly transient and localized dynamics of oxidative stress at zebrafish wound margins^2^ as well as the tissue quantities needed for LC–MS lipid measurements, a matching analysis of wounded tail fins was impractical. As we previously established that early leukocyte chemotaxis to injured tail fins requires 5-oxoETE along with Hcar1–4 (refs. 10,11), we instead decided to harness the leukocyte wound response as a proxy readout for 5-oxoETE generation at larval wound sites^10,11^. To this end, we counted the number of neutrophils (*lyz*^+^), macrophages (*mpeg*^+^) or both (transmitted light) at the injury site after triggering the wound response by hypotonic shock as previously described^10^ (Fig. 3b and Extended Data Fig. 6g–i). Similar to our previously published *hcar1–4* mutants (*hcar1–4*^*mk214/mk214*^)^11^ and morphants^10^, homozygous *dhrs7* mutants (*dhrs7*^*mk224/mk224*^ and *dhrs7*^*mk225/mk225*^) showed significantly impaired leukocyte recruitment to tail fin wounds (Fig. 3c,d, Extended Data Fig. 6g–i and Supplementary Videos 1 and 2). Consistent with previous findings^12,35^, macrophage chemotaxis was also inhibited (Fig. 3e,f and Supplementary Videos 3 and 4).

Bath isotonicity blocks rapid wound closure, biomechanical cytosolic phospholipase A2 (cPla_2_) activation and arachidonic acid release at zebrafish wound margins. The underlying osmo-responsive mechanotransduction mechanism has been extensively studied and reviewed elsewhere^11,36^. Since rapid leukocyte migration to zebrafish wound sites depends on 5-oxoETE, this response is diminished by isotonic bathing. Notably, leukocyte migration can be reconstituted either by switching to hypotonic bathing solution, which activates endogenous arachidonic acid production by cPla_2_ (Fig. 3b and Extended Data Fig. 6g–i), or by adding pure arachidonic acid (or 5(*S*)-HETE or 5-oxoETE) to the isotonic bath^10^.

If Dhrs7 mediates the conversion of arachidonic acid or 5(*S*)-HETE into 5-oxoETE at wounds, rapid leukocyte chemotaxis to externally provided arachidonic acid or 5(*S*)-HETE should be significantly impaired in *dhrs7* mutant but not wild-type larvae. Indeed, *dhrs7* disruption significantly blocked arachidonic acid- or 5(*S*)-HETE-dependent leukocyte chemotaxis to isotonic wounds (Extended Data Fig. 6j,k). The less complete inhibition of leukocyte migration to 5(*S*)-HETE-perfused, isotonic wounds aligns with the notion that 5(*S*)-HETE itself can act as a low-affinity agonist for the OXER1 receptor^37^. As expected, neutrophil chemotaxis to 5-oxoETE-perfused, isotonic (that is, ‘open’) wounds was unaltered in the *dhrs7* mutants. Of note, 5-oxoETE-mediated macrophage migration was significantly affected (Extended Data Fig. 6l–n). Single-cell RNA sequencing data suggest strong Dhrs7 expression in macrophages^22^. Macrophages could potentially harness Dhrs7 as an adaptive chemoattractant sink to steepen shallow 5-oxoETE gradients ‘on the go’. This idea is fully consistent with the known redox-dependent enzymology of Dhrs7 and the concept of self-generated chemotactic gradients^38^. Although it may elegantly explain the above observation, more work is required to validate this idea.

*dhrs7/hcar1–4* double mutation impaired acute leukocyte recruitment akin to the individual mutations, confirming that Dhrs7 and Hcar1–4 operate within the same acute, leukocyte recruitment pathway (Extended Data Fig. 6o,p). Tail fin regrowth after amputation was slightly impaired in *hcar1–4*^*mk214*/*mk214*^ but less so in *dhrs7*^*mk224*/*mk224*^ mutant larvae (Extended Data Fig. 6q,r). As both mutants exhibited similar leukocyte recruitment defects, these defects were unlikely to mediate this subtle regeneration phenotype. Whether it instead reflects trophic 5-oxoETE signalling functions remains to be investigated.

## Sterile inflammation in *dhrs7* mutant guts

DHRS7 and OXER1 mRNA and protein are expressed in the human digestive system, with highest mutual overlap in the small intestine^19^. In our previous work, we demonstrated that zebrafish Hcar1–4 is functionally orthologous to human OXER1^11^. According to single-cell RNA sequencing resources^22,39^, their orthologues are expressed in the zebrafish digestive tract. However, the role of Dhrs7 in the zebrafish digestive tract is unknown. Consistent with a loss of 5-oxoETE sink function under homeostatic (that is, unstressed) conditions (Fig. 3a), *dhrs7* mutants exhibited increased baseline neutrophil infiltration into their intestinal linings (Fig. 3g), which was sensitive to antioxidant treatment (Fig. 3i and Extended Data Fig. 7c). As intestinal apoptosis was unchanged in those fish (Fig. 3h and Extended Data Fig. 7a for representative images) and because the phenotype was insensitive to broad-spectrum antibiotics (Extended Data Fig. 7b), intestinal barrier defects with opportunistic microbial infection are unlikely to have contributed to it.

## Leaky gut barrier in *hcar1–4* mutants

*Hcar1–4* mutation (*hcar1–4*^*mk213/mk213*^ and *hcar1–4*^*mk214/mk214*^) increased baseline neutrophil infiltration (Fig. 4a and Extended Data Fig. 7d), as in *dhrs7* mutation or dextran sodium sulfate (DSS)-mediated damage of wild-type guts. Of note, *hcar1–4* and *alox5a*^26^ mutations abrogated the DSS-induced inflammation, implying its dependence on 5-oxoETE (Fig. 4a and Extended Data Figs. 6f and 7e). In mammalian animal models and human patients^40^, 5-LOX inhibitors such as Zileuton and MK-866 are known to improve colitis- or asthma-related symptoms, arguing for the physiological relevance of these zebrafish findings.

Whereas the sterile inflammatory *dhrs7* mutant gut phenotype was consistent with non-enzymatic 5-oxoETE accumulation, it was difficult to reconcile the pro-inflammatory effect of *hcar1–4* mutation with the established chemotactic role of 5-oxoETE^10,11,14^. As our previous work had shown increased expression of cell death-related transcripts (for example, *casp6a* and *casp6l1*) in *hcar1–4* mutant larvae^11^, we investigated whether the pathway might have anti-apoptotic effects on epithelial cells. Indeed, we observed a pronounced increase in intestinal apoptosis in *hcar1–4*, but not in *alox5a* or *dhrs7,* mutant fish (Figs. 3h and 4b and Extended Data Fig. 7f,g). Application of 5-oxoETE at a concentration lower than that used in the tail fin wounding experiments had no effect on intestinal neutrophil counts but reduced intestinal apoptosis in an *hcar1–4*-dependent manner (Extended Data Fig. 7h,i). Furthermore, *hcar1–4* mutation significantly increased gut barrier permeability as measured by fluorescent dextran leakage from the gut lumen into the adjacent interstitial space (Fig. 4c). Pharmacological inhibition of apoptosis normalized the baseline neutrophil numbers in *hcar1–4* mutant intestines (Fig. 4d and Extended Data Fig. 7j,k).

ROS are cytotoxic and can cause cell death of host cells as well as pathogens. These highly reactive and diffusive molecules may be generated when immune or intestinal epithelial cells become exposed to gut microbes, and have been ascribed both microbicidal and pro-repair functions^41^. However, hosts only benefit from ROS-mediated signalling and defence if they can effectively protect themselves against their toxic effects by increasing their own redox resilience. We conceived that Hcar1–4 signalling might contribute to such resilience.

Scavenging of hydrophilic ROS with Trolox rescued both apoptosis and neutrophilic inflammation (Fig. 4e) in *hcar1–4* mutant intestines, whereas blocking lipophilic ROS with LPX1 had little effect (Extended Data Fig. 7l,m). Thus, Hcar1–4 selectively suppressed the toxicity of hydrophilic ROS. To test whether neutrophils are the source of the ROS, we chemogenetically ablated nitroreductase (NTR2.0)-expressing neutrophils in *hcar1–4* mutants by bathing the larvae in metronidazole. Neutrophil depletion did not reduce intestinal apoptosis (Extended Data Fig. 7n). By contrast, treatment with broad-spectrum antibiotics rescued both the apoptosis and neutrophilic inflammation phenotypes in *hcar1–4* mutants (Fig. 4f). Together, these data suggest that *hcar1–4* deletion impairs the tolerance of intestinal linings towards microbiota-linked ROS in vivo, which in turn increases apoptotic damage, chronic barrier leakiness and inflammation.

## Protective OXER1 function in human cells

To test for phylogenetic conservation of the anti-apoptotic effects of OXER1, we silenced the human receptor in monolayer cultures of Caco-2 cells using either small interfering RNA (siRNA)- or CRISPR–Cas9-mediated knockdown (Extended Data Fig. 8a,c,d). OXER1 knockdown, but not non-targeting control (NTC) oligonucleotides, increased baseline apoptosis in serum-starved cells (Fig. 5a and Extended Data Fig. 8b). This was prevented by Trolox, but not LPX1, treatment (Extended Data Fig. 9a,b), mirroring our results in zebrafish. Furthermore, pretreatment with a physiologically relevant concentration of 5-oxoETE (1 μM) blunted H_2_O_2_-induced apoptosis compared with the vehicle control in an OXER1-dependent manner (Fig. 5b and Extended Data Fig. 9c,d). These experiments suggested that baseline OXER1 signalling protects Caco-2 cells against baseline ROS toxicity.

## The effect of OXER1 is mediated by NUDT1

Again, we sought clues for the putative resilience mechanism in our previously published *hcar1–4* mutant transcriptomics data^11^. Those data revealed a strong repression of *nudt15* transcription in *hcar1–4* mutants (Extended Data Fig. 9e), whereas other antioxidant systems showed little change. NUDT15 (also known as MTH2) is one of several NUDIX hydrolase enzymes (NUDT1, NUDT15 and NUDT18, also known as MTH1, MTH2 and MTH3, respectively) involved in the elimination of oxidized purine nucleotides, which have been shown to protect cultured cells from oxidative stress^42^. Silencing of NUDT1, but not NUDT15, increased apoptosis in Caco-2 cells compared with NTC (Extended Data Figs. 8g and 9f), highlighting NUDT1 as the relevant functional isoform in human cells. OXER1 and NUDT1 knockdown blunted NUDT1 protein expression as assessed by β-actin-normalized NUDT1 band densitometry (Extended Data Figs. 8e,f, 9g and 10a–d) or immunofluorescence (Extended Data Fig. 10e,f). Conversely, pretreatment with 5-oxoETE enhanced NUDT1 expression in wild-type, but not in OXER1-silenced, cells (Fig. 5c and Extended Data Fig. 10g–i). Notably, the anti-apoptotic effect of 5-oxoETE pretreatment was lost in NUDT1-silenced cells (Fig. 5d and Extended Data Fig. 10j,k). Together, these experiments argue for a mechanism in which NUDIX hydrolases (Nudt15 or NUDT1) mediate anti-apoptotic functions of OXER1 in zebrafish and human cells by protecting against the genotoxic effects of oxidized nucleotides.

## NUDT1 and Nudt15 protect *hcar1–4* mutant guts

To further test this idea in vivo, we used two complementary approaches: First, we knocked down NUDIX hydrolases in wild-type zebrafish with an F_0_ CRISPR approach (Fig. 5e and Extended Data Fig. 11a–f). Second, we overexpressed NUDIX hydrolases in the gut epithelium of *hcar1-*4 mutant larvae using the intestinal epithelium-specific *cldn15la* promoter (Fig. 5h). F_0_ CRISPR of *nudt15*, but not *nudt1*, increased neutrophil counts (Fig. 5f) and apoptosis in the intestine compared with NTC (Extended Data Fig. 11g). F_0_ CRISPR larvae for both *nudt1* and *nudt15* were grown to sexual maturity without overt developmental defects. F_0_ CRISPR of *nudt15* increased intestinal neutrophil counts and intestinal apoptosis in wild-type larvae but not in *hcar1–4* mutants (Fig. 5g and Extended Data Fig. 11h). As the *hcar1–4* and *nudt15* mutant phenotypes were not additive, both proteins are likely to form part of the same genetic pathway. Expression of zebrafish Nudt15 or human NUDT1 in the gut epithelium using the intestinal epithelium-specific *cldn15la* promoter reduced neutrophil counts in *hcar1–4* mutant guts back to control levels (Fig. 5h), underlining the functional redundancy of both enzymes and highlighting the need for a more detailed study of the physiological role of different NUDIX hydrolase enzymes.

## Discussion

Here we uncovered DHRS7 as a conserved, redox-regulated enzyme of the oxoeicosanoid pathway and find that oxoeicosanoid signalling protects intestinal linings from oxidative bystander damage, orchestrating its microbicidal, innate immune functions with epithelial resilience.

Owing to their pleiotropic chemical reactivity, ROS damage a broad range of known and unknown pathogens, along with nearby host cells that are not properly protected. Both the oxidant (NOX proteins) and antioxidant (such as reduced GSH–GSSG glutathione peroxidase–reductase) systems that manage immune-related oxidative stress require NADPH. If metabolism^43^ cannot resupply NADPH sufficiently quickly, the NADP^+^/NADPH and GSSG/GSH ratios increase. The temporary collapse of these antioxidant ‘floodgates’ permits membrane and protein oxidation^44^. Transient thiol oxidation of signalling proteins promotes the recovery of tissue homeostasis, in part through DNA protection, growth factor signalling, organelle remodelling, wound repair and regeneration^6,30^.

DHRS7 epitomizes a different, metabolic redox signalling paradigm in which the formation or degradation of a paracrine redox signal (5-oxoETE) depends directly on redox cofactor mass action, not protein oxidation. Like many other arachidonic acid-derived mediators, including prostaglandins, leukotrienes, lipoxins and specialized pro-resolving mediators, 5-oxoETE exerts its biological functions through a cognate G-protein-coupled receptor^37,45^, allowing changes in its metabolic production to be directly translated into cellular responses. DHRS7 metabolically couples innate immune cell recruitment to the respiratory burst activity of epithelial (Duox) or myeloid (NOX2) NADPH oxidases that often indicate pathogenic threats. Suppression of the sink function of DHRS7 by oxidative stress or by genetic deletion promotes 5-oxoETE accumulation, attracting leukocytes to damaged tail fins or intact intestinal linings. In addition, the OXER1 pathway prevents apoptosis of zebrafish and human intestinal cells by inducing DNA-protective NUDIX hydrolases^42^. Thus, oxoeicosanoid signalling not only triggers an innate immune response, but can also selectively protect the host against the damaging consequences of immune-linked ROS by increasing the redox resilience of mucosal barriers. Along with transient thiol oxidation of tyrosine kinases, this integrated mechanism may coordinate the pro-inflammatory and trophic functions of H_2_O_2_ at epithelial damage sites^2,3,30^.

The lack of clear OXER1 orthologues in rodents has severely impeded preclinical research into this pathway for the past 30 years. Although its causes are unknown, this conservation gap is no evolutionary oddity. Various biomedically relevant genes are conserved in fish and humans, but not in rodents—for example, human adenosine deaminase 2 (ref. 46). For studying specific, disease-relevant traits it is not sufficient to consider an organism’s mean evolutionary distance to humans, because the distance of its individual traits, such as, OXER1-mediated immune regulation, may markedly diverge from this average^47^. Combining alternative models with human cell culture approaches can thus help illuminating the ‘blind spots’ of mouse genetics. This approach led us to the identification of 5-HEDH activity as DHRS7. Although human and mouse and rat DHRS7 are more similar (78% amino acid identity, one-to-many comparison) than the human and zebrafish protein (58% amino acid identity, one-to-one comparison), mouse and rat DHRS7 showed little 5-HEDH activity in our microsome assay, unlike zebrafish, bovine and human DHRS7. Given the limited number of species and the unclear structural cause of this intriguing divergence, one must be cautious of overinterpretation. Nevertheless, the concomitant loss of OXER1 and DHRS7-dependent 5-HEDH-activity from rat and mouse seems to be notable and may point to the taxon-specific disappearance of an entire pathway. Using a systematic mutational approach and by modelling lipid ligand binding, we are currently seeking to identify the residues responsible for 5-HEDH substrate selectivity and activity. Once those are known, the question of whether the loss of OXER1 preceded the loss of the 5-HEDH function of DHRS7 or vice versa will become more approachable.

Because the catalytic direction of DHRS7 depends on cytoplasmic redox state and because DHRS7-independent production routes for 5-oxoETE exist, the inflammatory roles of the enzyme may vary depending on whether it generates or degrades 5-oxoETE. In undamaged tissue, when the NADP^+^/NADPH ratio is low, DHRS7 reductase function may limit the accumulation and diffusion of basal 5-oxoETE, the enzyme may have an anti-inflammatory function, in line with our observation in the zebrafish gut. However, when the NADP^+^/NADPH ratio is high, for example, at wound sites, DHRS7 boosts 5-oxoETE without limiting its diffusion, and the enzyme may have an overall pro-inflammatory function. When target cells, such as macrophages, express DHRS7, they may become their own 5-oxoETE gradient-shaping sinks. By dissipating the very signal that attracts them, DHRS7 expressing cells may both amplify the gradient they are following and contribute to the removal or the enhancement of the inflammatory cue at its source—that is, provide positive or negative feedback depending on whether oxidative stress at the source persists or has been resolved. Furthermore, OXER1 modulates inflammation in opposite ways: by protecting epithelial barriers from oxidative damage, it indirectly prevents barrier disruption and infection; and by recruiting leukocytes, OXER1 directly supports infection defence^11^. Thus, depending on redox state, DHRS7 can have anti-inflammatory or pro-inflammatory functions.

The integrated, pro-inflammatory and pro-resilience functions of 5-oxoETE may contribute to mammalian pathophysiology. Namely, tissue abundance of 5-oxoETE seems to correlate with mucosal health in patients with inflammatory bowel disease^13^, consistent with a mucoprotective role. Pharmacological OXER1 inhibition in primates not only inhibits leukocyte infiltration of bronchial linings, but is also linked to the disappearance of bronchial mucus-producing cells^12^, perhaps through apoptosis. Furthermore, 5-oxoETE was reported to promote the tissue-protective N2 polarization of neutrophils^48^. Pro-migratory function was previously shown for OXER1 in cultured cancer cells in vitro^49^, and deletory OXER1 and DHRS7 frameshift mutations occur in gastrointestinal and endometrial tumours^50^. OXER1 expression also appears to be prognostic for colorectal, pancreatic and renal cancer outcomes^19^. Future studies should determine whether or how these observations are linked to metabolic redox signalling and NUDIX-dependent mucosal protection.

## Online content

Any methods, additional references, Nature Portfolio reporting summaries, source data, extended data, supplementary information, acknowledgements, peer review information; details of author contributions and competing interests; and statements of data and code availability are available at https://doi.org/10.1038/s41586-026-11121-2.

## Methods

### General fish procedures

Adult wild-type and transgenic reporter casper^52^ zebrafish were maintained at the Memorial Sloan Kettering Cancer Center (MSKCC) Zebrafish Core Facility^53^, and all experiments were conducted in accordance with institutional animal healthcare guidelines, with approval from the Institutional Animal Care and Use Committee (IACUC) of MSKCC. Generation and genotyping of *alox5a* and *hcar1–4* mutants used in this study were described previously^54^. Both the leukocyte and apoptosis phenotypes were more robust in the *hcar1–4*^*mk214/mk214*^ line (this line has a large truncation and a mutation at an upstream part of the *hcar1–4* coding region, whereas the *mk213* line is mutated at a more downstream genomic region), so we used this line for the majority of the experiments. For wounding assays, larvae aged 2.5 to 3 days post-fertilization (dpf) were anaesthetized in isotonic E3 medium (145 mM NaCl, 0.17 mM KCl, 0.33 mM CaCl_2_, 0.33 mM MgSO_4_) containing 0.2 mg ml^−1^ ethyl 3-aminobenzoate methanesulfonate (Tricaine/Syncaine, Sigma, E10521). Oxoeicosanoid signalling in the intestines was studied in 6 dpf larvae. At 2.5–6 dpf, the sex of zebrafish larvae cannot be determined, and it is unlikely to influence the biological processes under study. For the adult lipidomics studies, both male and female fish were used (three biological replicates for each sex and genotype).

### Tail fin amputation and regeneration assays

For wounding experiments, the tail fin tips of anaesthetized 2.5–3 dpf larvae were carefully removed with a surgical microblade (Fine Science Tools, 10318) at the boundary of the notochord, ensuring the notochord remained uninjured^55^. For regeneration assays, the regenerated fin length was measured 3 days post-amputation, from the notochord to the distal edge of the regrown tissue^56^.

### Larval intestine experiments

For the chemical colitis experiments, DSS was obtained from Sigma (42867). On the day of the experiment, a 10% w/v stock of DSS was made in embryonic E3 medium as described previously^57,58^. DSS was further diluted to a working concentration of 0.25% w/v in E3. Larvae at 5 dpf (wild-type or mutant) were randomly distributed to E3 with or without DSS for 24 h. The fish were euthanized and fixed using 4% formaldehyde in PBS or PBSTX before further processing. ZVAD-FMK was obtained from ApexBio (A1902) dissolved in DMSO and stored as a 300 mM stock solution at −20 °C. The drug was used at a working concentration of 300 μM in E3 as described^59,60^. Larvae at 5 dpf were randomly distributed to E3 with ZVAD-FMK or 0.1% DMSO for 24 h. The fish were euthanized and fixed using 4% formaldehyde in PBS before further processing. Z-DEVD-FMK was obtained from MedChemExpress (HY−12466) dissolved in DMSO and stored as a 300 mM stock solution at −20 °C. The drug was used at a working concentration of 150 μM in E3 as described^61^. Larvae at 5 dpf were randomly distributed to E3 with Z-DEVD-FMK or 0.05% DMSO for 24 h. The fish were euthanized and fixed using 4% formaldehyde in PBS before further processing. NS3694 was obtained from MedChemExpress (HY-108356) dissolved in DMSO and stored as a 10 mM stock solution at −20 °C. The drug was used at a working concentration of 10 μM in E3 as described^61^. Larvae at 5 dpf were randomly distributed to E3 with NS3694 or 0.1% DMSO for 24 h. The fish were euthanized and fixed using 4% formaldehyde in PBS before further processing. Metronidazole was obtained from Sigma (PHR1052–1G) dissolved in DMSO and stored as a 100 mM stock solution at −20 °C. The drug was used at a working concentration of 150 μM in E3 as described by us and others^54,62^. *hcar1–4*^mk214/mk214^ tg(*lyz*:QF2 × 5×QUAS:NTR2.0) larvae at 5 dpf were randomly distributed to E3 with metronidazole or 0.15% DMSO for 24 h. The fish were euthanized and fixed using 4% formaldehyde in PBS with 0.05% Triton-X (PBSTX) before further processing. Ampicillin (BP1760–25) and kanamycin (BP906–5) were obtained from Fisher, dissolved in distilled water and stored at −20 °C. Antibiotics were used at a working concentration of 50 μg ml^−1^ and 15 μg ml^−1^ in E3 for ampicillin and kanamycin, respectively. Larvae at 4 dpf were randomly distributed to E3 with either ampicillin/kanamycin or 0.1% distilled water. Larvae were transferred to fresh E3 with or without antibiotics after 24 h. Fish were euthanized and fixed using 4% formaldehyde in PBS or PBSTX before further processing. Trolox was obtained from Selleck chemicals (S3665), dissolved in DMSO and stored as a 50 mM stock solution at −20 °C. The drug was used at a working concentration of 50 μM as described^63^. LPX1 (SML1414–5MG) was obtained from Sigma Aldrich dissolved in DMSO and stored as a 100 mM stock solution at −20 °C, and used at a working concentration of 20 μM as described^26^. For experiments with 5-oxoETE, the lipid was obtained as an ethanol-based solution (Cayman Chemical, 34250) and was used at a working concentration of 300 nM. Larvae at 5 dpf were randomly distributed to E3 with 300 nM 5-oxoETE or 0.1% ethanol for 16 h. The fish were euthanized and fixed using 4% formaldehyde in PBS or PBSTX before further processing.

### Whole-mount staining of larval intestines

#### Sudan Black.

Intestinal neutrophils were visualized by Sudan Black staining as described^11,26,64^. In brief, Larvae were fixed in 4% formaldehyde in PBS overnight at 4 °C, washed 3 times with PBS and stained with Sudan Black (Sigma Aldrich, 199664) for 1 h. Larvae were washed 3 times with 70% ethanol and rehydrated in PBS with 0.1% Tween (PBST) for 5 min. Larvae were then treated with depigmentation solution (1% KOH, 1% H_2_O_2_ in water) for 5 min followed by 2 PBST washes before imaging.

#### Active caspase-3 immunostaining.

Active caspase-3 was visualized using the protocol described with minor modifications^65^. Larvae were fixed in 4% formaldehyde in PBSTX overnight at 4 °C. Larvae were then washed with PBSTX and blocked for 2 h at room temperature in a blocking solution (PBSTX supplemented with 2 mg ml^−1^ bovine serum albumin (BSA) and 1% DMSO). Primary antibody (anti-active caspase-3 (BD Biosciences Product: 559565 and 570524)) was diluted 1:100 (for 559565) or 1:500 (for 570524) in blocking solution and larvae were incubated overnight at 4 °C. Larvae were washed with PBSTX and incubated with secondary antibody conjugated with Alexa Fluor 488 or 555 (Abcam, ab150077 or ab150078, respectively) diluted 1:1,000 in blocking solution overnight at 4 °C. Finally, larvae were washed with PBSTX and mounted for imaging.

### Microgavage of larval zebrafish

Microgavage was performed as described^66^. 4 kDa molecular weight FITC-labelled dextran (Sigma Aldrich FD4–100MG) was dissolved in PBS to obtain a 10 mg ml^−1^ solution. This solution was then loaded into a microinjector needle. Before gavage, 6 dpf zebrafish larvae were anaesthetized with 0.2 mg ml^−1^ MS-222 (Fisher Scientific, NC0589195) dissolved in E3. After verifying proper depth of anaesthesia, the larvae were positioned laterally, ventral side towards the vertical wall of an agarose mold. Afterward, the needle was gently manoeuvred down the oesophagus and into the anterior intestine. The dextran was then injected into the lumen (typically, volumes of 5–10 nl were used). The needle was then gently but firmly removed from the fish. Larvae were then transferred to E3 to recover from the anaesthesia and kept in E3 for 60 min and then used for imaging experiments.

### Generation of *dhrs7* mutant zebrafish lines and genotyping

Two independent guide RNAs (gRNAs) (*dhrs7*-sgRNA-1 and *dhrs7*-sgRNA-2) of zebrafish *dhrs7* (ENSDARG00000003444) were designed and ordered from IDT (integrated DNA Technologies). The Cas9–gRNA ribonucleoprotein complex (single *dhrs7*-sgRNA-1 or a combination of *dhrs7*-sgRNA-1 and *dhrs7*-sgRNA-2) was injected into the cytoplasm of 1-cell-stage zebrafish embryos. After the injected F_0_ larvae matured (2–3 months post-fertilization), individual F_0_ adults were crossed with wild-type adults to produce F_1_ progeny. These F_1_ larvae were then grown to sexual maturity, and genomic DNA was isolated from their tail fins for genotyping. Tail fins were partially amputated, suspended in 250 μl of 50 mM NaOH, and incubated at 95 °C for 10 min. Samples were then cooled on ice for 10 min, neutralized with 25 μl of 1 M Tris-HCl (pH 8), and vortexed. The genotyping primers are listed in Supplementary Table 2. PCR products from F_1_ *dhrs7*-sgRNA-1 adult fish were incubated with FastDigest PvuII (Thermo Fisher Scientific; FD0634) enzyme at 37 °C overnight. The reaction mixture was then separated by agarose gel electrophoresis. F_1_ heterozygous adults were identified by the presence of three DNA fragments (404, 340 and 68 bp). The 404 bp product represents a mutant allele where the PvuII site has been disrupted by Cas9-induced mutation. This 404 bp band was isolated from the agarose gel and sequenced via Sanger sequencing. F_1_ heterozygous adult zebrafish with the frameshift mutation of interest (4 bp deletion, *dhrs7*-1; Extended Data Fig. 6) were bred to achieve homozygosity.

PCR products from F_1_ *dhrs7*-sgRNA-1 and *dhrs7*-sgRNA-2 injected heterozygous adult zebrafish were also separated by agarose gel electrophoresis. The PCR products using the *dhrs7*-sgRNA-1 primers, digested with PvuII, revealed an in-frame mutation with a total 12 bp deletion (8 bp deletion and 4 bp deletion). Meanwhile, the PCR products using the *dhrs7*-sgRNA-2 primers, digested with BseLI (Thermo Fisher Scientific; FD1204), showed a frameshift mutation with a 2 bp deletion. The digested PCR products of *dhrs7*-sgRNA-2 were identified by the presence of three DNA fragments (252, 147 and 107 bp). The 252 bp product represents a mutant allele where the BseLI site has been disrupted by Cas9-induced mutation. This 252 bp band was isolated from the agarose gel and sequenced via Sanger sequencing, confirming the 2 bp deletion. F_1_ heterozygous adult zebrafish with the frameshift mutation of interest (14 bp deletion (8 bp deletion, 4 bp deletion and 2 bp deletion), *dhrs7–2*; Extended Data Fig. 6) were bred to achieve homozygosity.

### F_0_ CRISPR gene perturbation approach for *nudt1* and *nudt15*

Potential gRNA target sites were identified using the web program CHOPCHOP (http://chopchop.cbu.no/index.php). All gRNA target sequences are listed in Supplementary Table 2. Two independent gRNAs for *nudt1* (ENSDARG00000030573) and four independent gRNAs for *nudt15* (ENSDARG00000113961) were designed and ordered from IDT (for sequences see Supplementary Table 2). As a negative control, non-targeting guides were ordered from IDT (1072544,1072545 and 1072546). The target-specific or non-targeting Alt-R CRISPR RNA (crRNA) and common Alt-R *trans*-activating CRISPR RNA (tacrRNA, IDT 1072532) were dissolved in nuclease-free duplex buffer (IDT; 11–01-03–01) to obtain a 100 mM stock solution. To prepare the crRNA:tracrRNA duplex, equal volumes of 100 mM Alt-R crRNAs (1:1) and 100 mM Alt-R tracrRNA stock solutions were mixed to obtain a final concentration of 10 mM each. The mixture was heated to 95 °C for 5 min and then cooled down to room temperature. ALT-R *Sp*Cas9 Nuclease (IDT, 1081058) was diluted to 1 mg ml^−1^ with Cas9 working buffer (20 mM HEPES, 150 mM KCl, pH 7.5). The Cas9–gRNA ribonucleoprotein complex was assembled by combining the crRNA:tracrRNA duplex and Cas9 protein solutions at a 1:1 ratio, incubated at 37 °C for 5 min and then placed at room temperature. Cas9–gRNA ribonucleoprotein complex solution (2.3 nl) was injected into the cytoplasm of 1-cell-stage zebrafish embryos. At 2.5–3 dpf, genomic DNA was isolated from individual F_0_ larvae for genotyping. Each specific target sequence of interest was PCR amplified from the genomic DNA samples with the primers listed in Supplementary Table 2. Next, PCR products were incubated with the FastDigest (Thermo) restriction enzymes BccI (NEB, R0704S) for *nudt15-*g1, BseLI (FD1204) for nudt15-g2, MvaI (FD0554) for *nudt15*-g3, Cfr10I (FD0184) for *nudt15*-g4, BstXI (FD1024) for *nudt1*-g1, or in the case of *nudt1*-g2, BtsI-v2 (NEB, R0667S) for at least 2 h at 37 °C. Then, samples were separated by agarose gel electrophoresis (see Supplementary Table 2 for primers) and guide efficiency was evaluated (Extended Data Fig. 11).

### Plasmid construction for transgenesis

Total RNA was isolated from Caco-2 cells or 35–40 6 dpf *hcar1–4*^wt/wt^ larvae using Trizol (ThermoFisher 15596026) and 1 μg was used as a template for complementary DNA (cDNA) synthesis using the Revertaid First Strand cDNA synthesis kit (Thermo Scientific, K1622). The coding sequence of human NUDT1 and zebrafish NUDT15 were subcloned into a pME-MCS backbone between the Bsu15I and XbaI sites using T4 DNA ligase (NEB M0202S). The sequence fidelity of the resulting pME-HsNUDT1 and pME-DrNUDT15 plasmids was confirmed with Sanger sequencing. The *cldn15la*:QF2, *cldn15la*:Hs*NUDT1* and *cldn15la*:Dr*NUDT15* plasmids were created with Gateway cloning (LR Clonase II enzyme, Thermo Fisher 11791020) using the Tol2kit system as previously described^67^, recombining the *cldn15la* promoter, the QF2 transcriptional activator, Hs*NUDT1* or Dr*NUDT15* and a SV40 polyadenylation site into the pDestTol2CG2 (for *cldn15la*:QF2) or the pDestTol2CR destination vector (for *cldn15la*:Dr*NUDT15* or *cldn15la*:hsNUDT1). The pDestTol2CR vector was a gift from B. Enyedi^68^ and the p5E-cldn15la plasmid was a gift from J. Rawls (Addgene plasmid #125026; http://n2t.net/addgene:125026; RRID: Addgene_125026)^69^. A solution containing 25 pg of the *cldn15la*:QF2 and 25 pg Tol2 transposase mRNA was injected into the cytosol of 1-cell-stage wild-type embryos. For the rescue experiments in *hcar1–4*^*wt/wt*^ or *hcar1–4*^*mk214/mk214*^ fish, a similar mixture of *cldn15la*:HsNUDT1 or *cldn15la*:DrNUDT15 plasmid and Tol2 transposase mRNA was injected into the cytosol of 1-cell-stage *hcar1–4*^*wt/wt*^ or *hcar1–4*^*mk214/mk214*^ embryos. Injected larvae with cardiac green fluorescent protein (GFP, for *cldn15la*:QF2) or red fluorescent protein (mKate2, for *cldn15la*:HsNUDT1 or *cldn15la*:DrNUDT15) expression were selected 24 h post-fertilization. Transgenic embryos with NUDIX hydrolase expression were used for experiments in the F_0_ generation, while *cldn15la*:QF2 embryos were grown to adulthood and used to establish a stable transgenic line. The GFP expressing transgenic larvae shown in Fig. 5h were obtained by crossing *cldn15la*:QF2 and QUAS:eGFP^54^ fish and selecting fish showing GFP expression in the intestine.

### shRNA

The website http://splashrna.mskcc.org/show_results was used to design shRNA for all the genes mentioned in this manuscript (including *HSD17B4* (ENSG00000133835), *HSD17B12* (ENSG00000149084), *CBR1* (ENSG00000159228), *HADH* (ENSG00000138796), *DHRS3* (ENSG00000162496), *PYGB* (ENSG00000100994), *DHRS1* (ENSG00000157379), *DCXR* (ENSG00000169738), *RDH14* (ENSG00000240857), *LMNA* (ENSG00000160789), *TM7SF2* (ENSG00000149809), *RDH13* (ENSG00000275474), *PECR* (ENSG00000115425) and *DHRS7* (ENSG00000100612)). The corresponding DNA fragments of these shRNA sequences were ligated into the pSBbi vector using XhoI and EcoRI enzyme restriction sites. Stable cell lines expressing shRNA were obtained from pSBbi+gene transfected A549 cell lines after selection with blasticidin S hydrochloride (Thermo Fisher Scientific J61883.FPL).

### siRNA transfection of Caco-2 cells

Caco-2 cells were obtained from ATCC. Cells were maintained in EMEM (ATCC, 30–2003) supplemented with Pen-Strep (Corning, MT30002CI) and 20% heat-inactivated FBS (Sigma, F2442). A pool of 3–4 independent siRNAs targeting *OXER1* (L-005741–00-0005), *NUDT1* (L-005218–00-0005), *NUDT15* (L-020621–01-0005) or NTCs (D-0018–10-10–05) were ordered from Horizon Discovery, reconstituted in 1× siRNA buffer (5× siRNA buffer B-002000-UB-100, Horizon Discovery diluted to 1× with RNAse free water B-003000-WB-100, Horizon Discovery) at a concentration of 25 μM and stored at −20 °C. Cells were transfected with siRNA using Dharmafect 1 transfection reagent (T-2001–02) according to the manufacturer’s instructions. In brief, 5 μl of siRNA and 2.5 μl of Dharmafect 1 transfection reagent were diluted separately in 100 μl complete EMEM each, incubated at room temperature for 10 min and then mixed and incubated at room temperature for 20 min. Afterwards, 800 μl of complete EMEM was added and the mixture was added to the cells (250 μl per well of an ibidi 8-well dish). Cells were assayed 48 h after transfection. For Western blots in Extended Data Figs. 9g and 10a, Caco-2 cells were grown in 6-well plates and transfected with 1 ml siRNA transfection mixture per well.

### Generation of *DHRS7* mutant cell lines

Two independent gRNAs targeting human *DHRS7* (ENSG00000100612), *DHRS7*-sgRNA-1 and *DHRS7*-sgRNA-2 were designed and ordered from IDT (integrated DNA Technologies). The Cas9–gRNA ribonucleoprotein complex, composed of *DHRS7*-sgRNA-1, *DHRS7*-sgRNA-2, Alt-R CRISPR–Cas9 TracrRNA and Alt-R Cas9 enzyme, was introduced into A549 cells or HEK293 cells using the Neon transfection system following the manufacturer’s instructions. Transfected cells were then diluted to single colonies and plated in 96-well plates. After 2–3 weeks of cultivation, the single colonies were expanded in 6-well plates. Western blotting (for detailed protocol, see later) with a human DHRS7 antibody (abcam, ab156021) was performed to confirm DHRS7 knockout in the single colonies. β-Actin antibody (Sigma, A5441) was used as an internal control. Three independent knockout cell lines were selected for further research.

### Production of lentiviral particles and CRISPR–Cas9 knockdown Caco-2 cells

Lentiviral particles were produced by co-transfecting HEK293T cells with the lentiviral vector lentiCRISPRv2 containing the indicated sgRNAs (non-targeting sgRNA controls; *DHRS7*-sgRNA-1 and −3; *OXER1*-sgRNA1, −2, and −3; *NUDT1*-sgRNA1, −2, −3, and −4), the packing plasmid (psPAX2, Addgene #12260), and the envelope plasmid pCMV-VSV-G (Addgene #8454) using Lipofectamine 3000 reagents (Thermo Fisher Scientific, L3000008). Virus containing supernatants were collected 48 h after transfection, filtered through a 0.45-μm membrane, and used to infect Caco-2 cells in the presence of 10 ng ml^−1^ polybrene (Sigma, TR-1003-G) for 24 h. Infected cells were selected with 5 (for the cells infected with NTC and *OXER1*-targeting viruses) or 10 μg ml^−1^ (for the cells infected with *NUDT1*-targeting viruses) puromycin. All single guide RNA sequences are listed in Supplementary Table 2.

### RT–qPCR analysis of OXER1-knockdown Caco-2 cells

Caco-2 cells expressing the NTCs or *OXER1*-sgRNA1, *OXER1*-sgRNA2 or *OXER1*-sgRNA3 were plated in 6-well plates. For the data in Extended Data Fig. 8a, wild-type Caco-2 cells were transfected with NTC or *OXER1*-targeting siRNA. Total RNA was extracted using TRIzol reagent (Thermo Fisher Scientific, 15596026) according to the manufacturer’s instructions. cDNA was synthesized using the RevertAid First Strand cDNA Synthesis Kit (Thermo Fisher Scientific; K1621). Quantitative PCR with reverse transcription (RT–qPCR) was performed using Maxima SYBR Green/Fluorescein qPCR Master Mix (Thermo Fisher Scientific; K0242) on a Bio-Rad real-time PCR machine. Relative OXER1 expression was normalized to β-actin and calculated using the 2^−ΔΔ*C*t^ method^70^. Primer sequences are listed in Supplementary Table 2.

### DHRS7 overexpression and complementation across species and catalytic dead mutants

DHRS7 orthologues from *Homo sapiens* (ENSG00000100612), *Danio rerio* (ENSDARG00000003444), *M. musculus* (ENSMUSG00 000021094), *R. norvegicus* (ENSRNOG00000005589) and *B. taurus* (ENSBTAG00000020729) were cloned to pSBtet vector containing a C-terminal eGFP–6×His sequence. Human DHRS7 point mutations (K207E, Y203F, S190A, N162L, R82E) were generated and inserted to the pSBtet vector. All the pSBtet-DHRS7 constructs were co-transfected with the pCMV(CAT)-T7-SB-100 transpose vector into A549 cells (for co-localization assays) or HEK293T DHRS7-KO1 cells (for microsome experiments). Stable cell lines were selected for one week with 10 μg ml^−1^ blasticidin S hydrochloride, induced with 1 μg ml^−1^ Dox for 1–2 days, and subsequently screened by flow cytometry for eGFP-positive cells. For DHRS7 overexpression in Caco-2 cells, human DHRS7-eGFP was cloned to the pCW57-tet vector. Lentiviral production and infection were performed as described above. Stable cell lines were selected with 600 μg ml^−1^ G418 (Thermo Fisher Scientific, 10131035) for 7–10 days, induced with 1 μg ml^−1^ Dox for 1–2 days, and subsequently enriched by flow cytometry sorting for eGFP-positive cells.

### Protein expression and purification

DHRS7 from *H. sapiens* (UniProt ID: Q9Y394) and *D. rerio* (UniProt ID: Q0P3U1) was expressed in HEK293S GnTI^−^ cells (ATCC, CRL-3022), using the inducible stable cell line system. Briefly, the *Hs*DHRS7 and *Dr*Dhrs7 cDNA sequences were cloned into the pSBtet vector containing a C-terminal 3C–eGFP–3×Flag–10×His sequence. The stable cell lines were generated by co-transfection of pSBtet-hsDHRS7 (or pSBtet-drDhrs7) and the pCMV(CAT)-T7-SB-100 transpose vector, followed by 1 week of selection with 10 μg ml^−1^ blasticidin S hydrochloride. All plasmids used in this study are listed in Supplementary Table 3.

The stable cells were grown in suspension at 37 °C in FreeStyle 293 Expression Medium (Thermo Fisher Scientific, 12338026) with 2% (v/v) FBS under 8% CO_2_. When the cells reached a density of 2.0–2.5 × 10^6^ cells per ml, 1 μM Dox and 10 mM sodium butyrate were added, respectively, and the growth temperature was lowered to 30 °C to boost protein expression. After 48 h of treating Dox and sodium butyrate, the cells were collected by centrifugation at 550*g* and resuspended in lysis buffer containing 20 mM Tris-HCl, pH 8.0, 150 mM NaCl, 10% (v/v) glycerol supplemented with cOmplete protease Inhibitor Cocktail tablet EDTA-Free (Roche, 04693132001). The cells were flash-frozen in liquid nitrogen and stored in −80 °C.

All protein purification steps were performed at 4 °C. Thawed cell pellets were processed for microsome extraction as described above, then solubilized with 1% (w/v) *n*-dodecyl-β-d-maltoside (DDM) for 1 h. Insoluble materials was removed by centrifugation at 33,745*g* for 1 h. The supernatant was subsequently incubated with prewashed anti-Flag M2 resin (Sigma Aldrich, A2220) for 1 h and washed with 10 column volumes of washing buffer (microsome purification buffer containing 0.1% (w/v) DDM). The *Hs*DHRS7 or *Dr*Dhrs7 proteins were eluted using 5 column volumes of elution buffer (washing buffer containing 0.2 g l^−1^ 3×Flag peptide (Sigma Aldrich, F4799)).

To remove the affinity tags, 3C proteases were then added to the eluted proteins to remove the eGFP and 3×Flag–10×His tag before the elution was concentrated by a 50-kDa MWCO spin concentrator. The sample was then purified by size-exclusion chromatography on a Superdex 200 increase 10/300 GL column (Cytiva, 28–9909-44) equilibrated with washing buffer containing 0.003% (w/v) DDM. Peak fractions were pooled and used for the further activity assays.

### Flow cytometry and cell sorting

Stable cell lines were established by fluorescence-activated cell sorting of eGFP-positive cells at the MSKCC Flow Cytometry Core on BD FACSAria (Aria5/Aria6), FACSymphony S6 and FACSDiscover S8 instruments. Cells were gated sequentially: intact cells on FSC-A/SSC-A (P1); single cells by two successive doublet-discrimination gates (P2, P3); and eGFP-positive cells on the eGFP channel versus a second fluorescence channel to exclude autofluorescence (P4), with the positive gate set relative to a GFP-negative control (0% in P4). The gating and sorting strategy is shown in Supplementary Fig. 2. Flow cytometry was used solely to enrich fluorescent protein-expressing cells during line generation; no flow-cytometric data are presented as experimental results.

### NADP^+^/NADPH ratio experiments

HEK293T DHRS7-knockout and DHRS7-overexpression cells were plated in 96-well plates, 1 mM H_2_O_2_ was added to the cells for 20 min. Then the NADP^+^/NADPH ratio was detected using the NADP/NADPH-Glo Assay (Promega, G9082). In brief, each well of cells was washed with 50 μl PBS, followed by homogenization with 50 μl of base solution containing 1% DTAB. Next, 50 μl of each sample was transferred to an empty well and mixed with 25 μl of 0.4 N HCl. The plate was covered and incubated at 60°C for 15 min, then cooled down to room temperature. To neutralize the acid, 25 μl of 0.5 M Trizma base was added to each well containing acid-treated cells and add 50 μl of HCl–Trizma solution was added to each well containing base-treated samples. Finally, an equal volume of NADP/NADPH-Glo Detection Reagent was added to each well, and the plate was gently shaken and incubated for 30 min at room temperature. Luminescence was recorded using Gen5.

### Sample collection and lipid extraction

For the A549 cell culture LC–MS lipid detection, 1,000,000 cells were plated to 6-well plate and incubated at 37 °C overnight. The next day, cells were washed three times by PBS. Then, 1 μM 5-oxoETE or 1 μM 5(*S*)-HETE was added to 1.5 ml of Leibovitz’s L15 medium (Thermo fisher Scientific; 21083027) and introduced to each well. The cells were incubated at 37 °C for 20 min. The supernatant was collected for the LC–MS analysis.

For the LC–MS lipid detection in *Hs*DHRS7-knockout A549 cells under different treatments, 500,000 cells were seeded into 6-well plates and incubated at 37 °C overnight. The next day, 0.2 μg ml^−1^ Dox was added to induce DHRS7–eGFP expression for 24 h. On the third day, cells were treated with either 1 μM RSL3, 1 μM or 5 μM IKE, 20 μM LPX1 or 50 μM Trolox overnight (18 h). After RSL3 or IKE treatment, 1 μM 5(*S*)-HETE was added for 20 min. For H_2_O_2_ treatment, 1 mM H_2_O_2_ and 1 μM 5(*S*)-HETE were added to the medium, with or without 20 μM LPX1 or 50 μM Trolox for 20 min.

Before lipid extraction, 10 μl of a standard solution (0.5 μg ml^−1^ 5(*S*)-HETE-d8 and 0.5 μg ml^−1^ 5-oxoETE-d7) was added to each sample. The eicosanoid extraction was performed with some modifications of an earlier method^71^. Samples were extracted using Sola Solid Phase Extraction Plates (Thermo Fisher Scientific; A00707). In brief, the columns were conditioned with 3 ml of 100% methanol and then equilibrated with 3 ml of double-distilled H_2_O. Samples were loaded onto the columns, and the sample flow throughs were reloaded to ensure efficient binding. The columns were washed twice with 1 ml of H_2_O: Methanol (90:10 by v/v) to remove impurities. Metabolites and lipids were eluted with twice 500 μl of 100% methanol. Prior to LC–MS analysis, samples were evaporated using a SpeedVac and reconstituted in 50 μl of H_2_O: acetonitrile:acetic acid (60:40:0.02 v/v/v).

For LC–MS lipid detection in HEK293T microsomes, cells from 10 plates of each genotype were collected by centrifugation at 3,000*g* for 10 min at 4 °C. The pellets were resuspended in 50 ml NTE buffer (150 mM NaCl, 15 mM Tris-HCl, 1 mM EDTA, pH 7.5), and centrifuged again at 3,000*g* for 10 min at 4 °C. The resulting pellets were resuspended in cold cell lysate buffer (0.1 M potassium phosphate buffer pH 7.5, 250 mM sucrose, 50 mM KCl, 1.1 mM EDTA, 0.1 mM DTT, 1:1000 protease inhibitor cocktail (Millipore Sigma, 11873580001)). The cell suspension was lysed using a Dounce homogenizer with 50–60 strokes. To remove unbroken cells, cellular debris, nuclei and mitochondria, the lysate was centrifuged at 12,000*g* for 20 min at 4 °C. Microsomes were isolated by ultracentrifugation at 186,000*g* for 1–2 h and resuspended in cold resuspension buffer (0.1 M potassium phosphate buffer pH 7.5, 50 mM KCl, 1.1 mM EDTA, 0.1 mM DTT, 20% (v/v) Glycerol, 1:1000 protease inhibitor cocktail). Protein concentration was determined by the Pierce BCA Protein Assay Kits (Thermo Fisher Scientific, PI23227). The concentration of *Hs*DHRS7–eGFP and *Dr*Dhrs7–eGFP was quantified using eGFP standard.

The enzyme assay was performed as follows: 1 ml reaction buffer (90 mM potassium phosphate, 40 mM KCl, 0.1 mM DTT, pH 7.5) containing microsomes from *Hs*DHRS7-KO1 (20 μg) and *Hs*DHRS7-overexpression (20 μg, containing 4.39 pmol *Hs*DHRS7–eGFP), or *Hs*DHRS7-knockout (16 μg) and *Dr*Dhrs7-overexpression (16 μg, containing 4.39 pmol *Dr*Dhrs7–eGFP), 10 μg microsomes (HEK293T, DHRS7-KO1, DHRS7-KO2, DHRS7-KO3), 100 μM NADP^+^ or 100 μM NADPH, 1 μM 5(*S*)-HETE or 1 μM 5-oxoETE was incubated at 37 °C for 20 min. The reaction was stopped by placing the tubes on ice and adding 10 μl of a reference standard solution (0.5 μg ml^−1^ 5(*S*)-HETE-d8 and 0.5 μg ml^−1^ 5-oxoETE-d7), followed by 4 ml hexane. The aqueous phase was acidified to approximately pH 3.0 by adding 20 μl of 4 N HCl to facilitate the extraction of carboxylic acids. Samples were vortexed and centrifuged at 3,000*g* for 10 min at 4 °C to ensure phase separation and remove precipitated materials. The organic phases were collected, evaporated using a SpeedVac, and reconstituted in 150 μl buffer A (water/acetonitrile/acetic acid, 60/40/0.02, v/v/v).

Whereas absolute 5-oxoETE/5-HETE baseline levels often varied between individual lipid extractions (for example, Fig. 1b versus Fig. 1c), within the same extraction–LC–MS session, the relative differences were consistent (for example, Fig. 1b, − versus + 5(*S*)-HETE or 5-oxoETE, or Fig. 1c, WT versus KO). To account for these technical variations, each analytical batch was processed with its corresponding wild-type controls, and absolute lipid concentrations were not directly compared between batches.

### LC–MS/MS analysis

All samples were analysed using a Vanquish UHPLC system (Thermo Scientific 8362543) coupled to an ID-X Tribrid mass spectrometer (Thermo Fisher Scientific FSN30160) equipped with a heated electrospray ionization (HESI) probe. For eicosanoid analysis, reversed-phase separation was performed on an Acquity UPLC BEH Shield RP18 column (2.1 × 100 mm, 1.7 μm; Waters) as previously described^72^. The mobile phase consisted of (A) acetonitrile/water/acetic acid (60/40/0.02, v/v) and (B) acetonitrile /IPA (50/50, v/v). The stepwise gradient conditions were carried out for 10 min as follows: 0–5.0 min, 1–55% of solvent B; 5.0–5.5 min, 55–99% of solvent B, and finally 5.5–6.0 min, 99% of solvent B. The flow rate was maintained at 0.5 ml min^−1^, with an injection volume of 10 μl. Samples were kept at 4 °C during analysis, and the column temperature was set to 50 °C. Mass spectrometer parameters were as follow: ion transfer tube temperature, 300 °C; vaporizer temperature, 275 °C; Orbitrap resolution MS1, 120,000, MS2, 30,000; RF lens, 60%; maximum injection time MS1, 50 ms, MS2, Auto; AGC target MS1 and MS2 were Auto. Samples for analysis were run in both negative and 2,500 V used for negative ion voltage; Aux gas, 10 units; sheath gas, 40 units; sweep gas, 1 unit. HCD fragmentation was performed using stepped collision energies of 15%, 25%, and 35%. Full-scan mode with data-dependent MS2 (ddMS2) was applied across a mass range of *m*/*z* 200–600. Internal calibration was performed using EASY-IC. A scheduled targeted inclusion mass list was used for the analysis, including 5-HETE, 5-oxoETE, 5-HETE-d8 and 5-oxoETE-d7. Peak identifications were performed using Excalibur 3.1 (Thermo Scientific, OPTON-30965). The retention time, precursor ion mass/charge, and fragmentation patterns of 5-oxoETE, 5-HETE, 5-oxoETE-d7, and 5-HETE-d8 were confirmed using purchased standards. The following transitions were monitored: 5-oxoETE: 317.2122 > 203.1806, 5-HETE: 319.2280 > 115.0402, 5-oxoETE-d7: 324.2563 > 210.2242, 5-HETE-d8: 327.2783 > 116.0463. For quantification, the area under the curve (AUC) for 5-HETE and 5-oxoETE was normalized to the AUC of the corresponding internal standards (5-HETE-d8 and 5-oxoETE-d7, respectively).

For the adult tail fin lipidomic analysis, 0.1 g of tail fin tissue from 3–6-month-old of *dhrs7*^*wt/wt*^ and *dhrs7*^*mk224/mk224*^ zebrafish were collected. The samples were sent to the University of California, San Diego Lipid Maps facility for LC–MS analysis. The tail fin tissues were homogenized in 1 ml 10% methanol in water using a Bead Mill 24 (Fisher Scientific, 15–340-163). Internal standards were added to 100 μl homogenate. Eicosanoids were extracted using solid-phase extraction with Phenomenex Strata-X polymeric reversed-phase columns. The samples were dried and reconstituted in buffer A (water/acetonitrile/acetic acid, 60/40/0.02, v/v/v). Analysis was performed with a Water Acquity UPLC interfaced with an AB Sciex 6500 QTrap instrument. Chromatographic separation was achieved with a step gradient from 100% buffer A to 100% buffer B (acetonitrile/isopropanol, 50/50, v/v) over 5 min. Standard curves were generated under identical conditions. Data analysis was conducted using Analyst and MultiQuant software packages^73^. The raw lipidomic data output are presented in Supplementary Table 1. Sex-associated differences were assessed separately for each lipid after log_2_ transformation with a lipid-specific pseudocount equal to one-half of the smallest positive concentration, using an exact balanced permutation test that preserved genotype and the allocation of 3 females and 3 males within each genotype (20 assignments per genotype; 400 joint permutations); the overall sex effect was defined as the average within-genotype male-minus-female contrast, genotype-by-sex interaction as the difference between mutant and wild-type contrasts, and Benjamini–Hochberg correction was applied separately to each family of *P* values, with *q* < 0.05 considered significant^74–78^.

For each lipid species, wild-type and *dhrs7* mutant groups were compared using six biological replicates per genotype, comprising three females and three males. Fold change was calculated from the untransformed concentrations as the mutant group mean divided by the wild-type group mean, such that values greater than 1 indicate higher concentrations in mutants and values below 1 indicate lower concentrations. For heat map visualization, concentrations were standardized separately for each lipid across the 12 samples by subtracting the row mean and dividing by the row standard deviation. Consequently, heat map colours represent relative within-lipid abundance across individual fish and do not indicate absolute concentration differences between different lipid species. Sex effects and genotype-by-sex interactions were evaluated separately using the exact balanced permutation analysis described above and were not used to select the lipids displayed in the genotype heat map (Fig. 3a).

### Expression pattern of DHRS7 in different cell components

HEK293T cells from 10 plates were collected and lysed as previously described. The nuclear pellet was collected by centrifugation at 1,000*g* for 10 min at 4 °C. The supernatant was then transferred to a new tube, and the granule pellet was collected by centrifugation at 15,000*g* for 10 min at 4 °C. The supernatant was transferred again to a new tube, and the microsome pellet was collected by high-speed centrifugation at 186,000*g* for 1–2 h at 4 °C. The supernatant was used as the cytosol.

### NADPH production assay

For the NADPH production assay using different cell components, 10 μg of nuclear, granule, microsome and cytosol fraction were incubated with 1 μM 5(*S*)-HETE and 100 μM NADP^+^.

For the microsomal NADPH detection extraction experiments, *Hs*DHRS7 (human), *Dr*Dhrs7 (zebrafish), DHRS7B, DHRS7C, DHRS9, RDH11 and 15-PGDH were cloned and ligated to the pSBtet-eGFP vector. These constructs were transfected into HEK293T cells and selected using 10 μg ml^−1^ blasticidin S hydrochloride. Blasticidin-resistant cells were then treated with 1 μg ml^−1^ Dox for 24 h, GFP-positive cells were sorted via flow cytometry to establish stable cell lines.

The enzyme assay was performed in a total volume of 100 μl containing reaction buffer (90 mM potassium phosphate, 40 mM KCl, 0.1 mM DTT, pH 7.5), the indicated amount of microsomes, 100 μM NADP^+^ or 100 μM NAD^+^, and 1 μM 5(*S*)-HETE. NADPH (NADH) fluorescence was detected using Gen 5 plate reader with excitation at 340 nm and emission at 445 nm. The assay was conducted at 37 °C with readings taken every minute for 60–90 min.

Reactions were initiated by the addition of substrate immediately before placing the plate into the gen5 reader. Consequently, the first fluorescence measurement was acquired after an unavoidable delay between reaction initiation and data acquisition.

### Determination of Michaelis–Menten parameters

The first 20 time points of the NADPH kinetics were fitted with a line to approximate initial velocities (*v*_0_). The *R*^2^ values of each of these fits were averaged to yield an overall *R*^2^ value. Next the *v*_0_(NADP+) and *v*_0_(5(*S*)-HETE) isotherms were fitted with a Michaelis–Menten model *v* = *V*_max_ × [S]/(*K*_m_ + [S]), with [S] being the substrate concentration, *V*_max_ being the maximal enzyme velocity, and *K*_m_ the Michaelis constant (indicating half-maximal enzyme saturation). Fitting and figure plotting was performed in MATLAB 2024b (Update 4,24.2.0.2833386, 64-bit, win64, MathWorks) using the base functions and the optimization and statistics and machine learning toolboxes (v24.1; lsqcurvefit). For better readability, the corresponding live script MMcalculation.mlx was edited/annotated using ChatGPTo1 (OpenAI). This script obtained all numerical NADPH(*t*) data from a formatted Excel file (MichaelisMenten.xlsx), in which the NADPH concentration data was provided (in μM) in replicates as columns for each substrate concentration. For more details, please refer to the annotated MATLAB script.

### Western blotting

Cells were grown in six-well plates to confluence, washed once with ice-cold PBS and collected in RIPA buffer (Cell Signaling, 9806) containing protease inhibitors (Roche, 11836153001). Lysates were centrifuged at 18,000 *g* for 10 min at 4 °C and protein content was determined using the BCA assay (Thermo Scientific, A65453) according to the manufacturer’s instructions. Lysates containing 10 μg protein were treated with reducing agent (50 mM DTT, Thermo, NP0009) and sample loading buffer (NP0007, Thermo), boiled for 10 min and subjected to SDS–PAGE on precast gradient gels (NP0322, Thermo) at 120 V for 120 min. Proteins were transferred to nitrocellulose membranes using standard procedures. Membranes were blocked in 5% skimmed milk dissolved in TBS containing 0.1% Tween-20 (TBS-T) for 2 h at room temperature. Primary antibodies were diluted in TBS-T containing 3% BSA and rocked at 4 °C overnight (anti-NUDT1 (abcam, ab200832), 1:5,000; anti-β-actin (Sigma, A5441), 1:5,000, anti-DHRS7 (Abcam, ab156021), 1:1,000, anti-eGFP (Abcam, ab184601), 1:1,000). Afterwards, blots were washed 5 times with TBS-T at room temperature and incubated with secondary antibodies conjugated to HRP (anti-mouse (Cell Signaling, 7076S), 1:4,000, anti-rabbit (Cell Signaling, 7074S), 1:2,000) diluted in TBS-T containing 2.5% skimmed milk for 1–2 h at room temperature. Blots were washed 5 times in TBS-T and signals were detected using an enhanced chemiluminescent detection reagent according to manufacturer’s instructions (Cytiva, RPN2235). For quantification, images were background subtracted and the respective band intensities were quantified using the straight line tool and mean grey value function in ImageJ. Full-length, uncropped images of all western blots and gels are provided in Supplementary Fig. 1.

### Drug treatments of Caco-2 cells

For experiments, cells were trypsinized and seeded into 8-well precoated imaging dishes (ibidi, 80826) at a density of 8,000 cells per well and grown to confluence for experiments. Trolox was used at a working concentration of 50 μM and LPX1 was used at a working concentration of 20 μM in serum-free EMEM. For experiments with 5-oxoETE, the lipid was obtained as an ethanol-based solution (Cayman Chemical, 34250) and was used at a working concentration of 1 μM in serum-free EMEM. Cells were treated with the compounds or the appropriate vehicle control overnight. For H_2_O_2_ treatment experiments, a 1 M stock solution was diluted from 30% H_2_O_2_ on the day of the experiment. Cells were treated with 1 mM H_2_O_2_ or vehicle control diluted in serum-free EMEM for 2 h.

### Immunofluorescent staining of Caco-2 cells

Immunofluorescent staining of fixed Caco-2 cells was performed as described previously with some modifications^79^. In brief, cells were fixed with 4% formaldehyde in PBS at room temperature for 15 min, washed 3 times with PBS, permeabilized with PBSTX for 15 min and blocked with 5% goat serum (Jackson Immunoresearch, 005–000-121) in PBS at room temperature for 1 h. Cells were incubated with primary antibodies (anti-active caspase-3 1:200 BD Biosciences Product: 559565 and 570524, anti-NUDT1, (Abcam, ab200832) 1:100) diluted in blocking solution at room temperature for 1.5 h. Cells were washed 3 times with PBS and then incubated with secondary antibodies (1:500) diluted in blocking solution at room temperature for 30 min. Cells were washed twice with PBS and stained with DAPI (1:5000) in PBS for 15 min. Cells were washed 2 more times with PBS before imaging.

### Immunofluorescent staining of A549 cells with endoplasmic reticulum and mitochondrial antibodies

A549 cells were stained as detailed above, with the exception that the A549 cells were incubated at room temperature for 1.5 h with primary antibodies diluted in blocking solution: anti-Calnexin (endoplasmic reticulum marker, 1:100, Cell Signaling Technology, 2679), anti-Cox4 (Mitochondria marker, 1:400, Cell Signaling Technology, 4850S) and anti-lamin A/C (Nucleus marker, 1:100, Cell Signaling Technology, 4777).

### Spinning disc confocal imaging

For live imaging^80^, 2.5–3 dpf anaesthetized Tg(*lyz*:pm2-mKate2) and Tg(*mpeg1*: eGFP) larvae were amputated in Isotonic E3 tricaine, and immobilized in ~200 μl of 1% low-melting agarose (Gold Biotechnology, A-204–100) dissolved in Isotonic E3, placed in glass-bottom dishes (MatTek Corporation, P35G-1.5–20-C). After solidification of the agarose, the larvae were covered with 2–3 ml Isotonic E3 Tricaine to prevent and maintain anaesthesia during imaging. After 5 min of imaging, the buffer was replaced with the indicated medium for 1 h imaging sessions. The medium used included Isotonic E3 tricaine with 7.5 μM arachidonic acid (Sigma, A3611), 5 μM 5(*S*)-HETE (Cayman, 34230) or 1 μM 5-oxoETE (Cayman, 34250). Neutrophils are counted once they had exited the blood vessels.

Imaging was performed at 28 °C in a heated imaging chamber (TOKAI HIT, WPI inc) using an inverted Nikon Eclipse Ti microscope. The microscope was equipped with a CFI Plan-Apochromat Lambda D 10× objective lens (NA 0.45), a motorized stage, a Yokogawa CSU-W1 Spinning Disk unit, a Photometrics Prime BSI Scientific CMOS camera (2 × 2 binning), and NIS imaging software (Nikon, 5.11.01). Fluorescence emission was excited using 488 or 561 nm diode lasers (Nikon). Channel acquisition intensities/exposure times were set at 50%/100 ms for the 488 nm laser power or 80%/100 ms for the 561 nm laser power to detect either macrophages or neutrophils, respectively. Emission was collected using 525/36 (green) or 605/52 (red) bandpass filters with a high-sensitive sCMOS camera using bandpass dichroic mirrors (Chroma Technology Corp., 89100bs) for filtering two separate fluorescence emission spectra (525/36 and 605/52, Chroma Technology) placed in front of the detector for isolated detection of green and red fluorescence, respectively.

### Super-resolution fluorescence microscopy

To determine the subcellular localization of *Hs*DHRS7–eGFP (green), we quantified its spatial overlap in A549 cells with organelle-specific immunofluorescence signals, including mitochondria (Cox4), endoplasmic reticulum (calnexin) or nuclear (lamin A/C). For pixel-wise co-localization analysis, SIM-phase (phase = 5–9, 27.5 μm G5) images were acquired using a 63× objective (Plan-Apochromat 63× NA = 1.4∞/0.17 WD = 0.19 mm Oil, 420782–9900-000 Zeiss) oil immersion lens and the Zeiss Elyra 7 microscope and reconstructed in SIM^2^ for 60 nm super-resolution microscopy. The samples were excited with OPSL lasers (488, 561 nm), red fluorophores were excited by 561 nm (4% power) and eGFP by 488 nm (4% power) laser lines, respectively. The light path is equipped with reflector turret mounted with FW1:LBF (405/488/561/642-nm laser blocking filter) beam splitters and filters for isolated 2x pco.edge sCMOS (v4.2 CL HS, innovate LCS-BU) isolated camera detection for red (TV1: LP570) or green (TV2:BP 495–550) emission. The acquisition of individual *z*-stacks takes about 12–20 s and 50–80 ms exposure time for both colours. The multiphase SIM data were recorded in apotome- or lattice- 3D leap modes with an FOV of 80.14 μm × 80.14 μm (1,280 × 1,280 pixels), 0.063 μm pixel size, 0.110–0.329 μm *z*-step, stack 5–10 μm and collected at 16-bit resolution (pco.edge sCMOS, Sensor Temperature, Air 7 °C).

SIM and Lattice-SIM reconstruction is performed with the SIM processing Tool of the ZEN 3.0 SR FP2 (64-bit, 16.0.20.306 black) software for applying SIM^2^ Image reconstruction. The advanced filter is set with best fit and Gauss (sectioning = 100), no detrending is used for either channel or scale to raw image. The set regularization weight is set to 0.015, with proc.Sampling and Out.Sampling set to ×2 or ×4 and ran the algorithm for at least 20 iterations. After image reconstruction, the voxel size decreased to 0.016 × 0.016 × 0.110–0.329 μm (Out.Sampling). The reconstructed SIM^2^ images were subsequently loaded into custom Fiji java macro for obtaining maximum-intensity-projected images that were automatically saved as.TIFF output and analysed in a custom Python script (https://github.com/zazadovv/Redox-DHRS7_subcellular.git). The obtained files were then analysed for region of interest with image segmentation on intensity thresholds and Pearson’s correlation coefficients as well as Manders M1/M2 coefficients. Co-localization analysis was performed using custom Python (v3.9.23) scripts built on NumPy (v1.25.2^81^), SciPy (v1.9.3^82^), scikit-image (v0.24.0^83^), tifffile (v2023.2.28^84^), matplotlib (v3.7.5^85^) and the napari image viewer (v0.4.19^86^) with magicgui (v0.10.1^87^) extensions and QtPy (v2.4.3). GPU acceleration (when available) was provided by CuPy (v13.6)^88^.

### Epifluorescent microscopy

#### Imaging of larval intestines.

Larvae were embedded in 200 μl 1% low-melting agarose in a glass-bottom dish (MatTek Corporation; P35G-1.5–20C). Larvae were oriented as shown in the schematic in Figs. 3g, 4b, 5e and Extended Data Fig. 7a (Larval zebrafish graphic was sourced from Scidraw.io (courtesy of L. Petrucco^51^)). For the experiments with live fish, E3 medium with tricaine was added to the top of the agarose after it solidified to prevent desiccation of the sample and maintain anaesthesia over the course of the imaging. Transmitted light images were acquired at room temperature (~25 °C) using NIS imaging software (Nikon, 5.21.03-build 1489). on a Nikon Eclipse Ti microscope equipped with a 10×, Plan-Apochromat NA 0.45 air objective lens, and an Andor Clara charge-coupled device (CCD) camera. Alexa 488 and FITC fluorescence was excited using an LED light source (Lumencor 7; Light Engine) with an internal 475/28 bandpass filter, and Alexa 555 fluorescence was excited using an internal 549/15 bandpass filter. The emission of Alexa 488 and FITC was sampled using a 525/50 bandpass filter (Chroma; 525/50 m), while the emission of Alexa555 was detected using a 632/60 bandpass filter (Chroma; ET632/60 m). *z*-stacks were acquired with a 5 μm step size with a total imaging depth of 80 μm.

#### Imaging Caco-2 cells.

Caco-2 cells were imaged on the same setup detailed previously for imaging active caspase-3 immunostained zebrafish larvae. DAPI fluorescence was excited using an LED light source (Lumencor 7; Light Engine) with an internal 390/18 bandpass filter and detected using a 460/50 bandpass filter (Chroma; ET460/50 m). Images were acquired in a single *z*-plane.

### AlphaFold2 predictions of DHRS7 and hypothetical membrane positioning

AlphaFold2.PDB files of DHRS7 orthologues were retrieved from the AlphaFold Protein Structure Database from the listed species in Extended Data Fig. 2a. Mammalian models comprised *H. sapiens* (AF-Q9Y394-F1-v6), *Pan troglodytes* (AF-A0A6D2X0S6-F1-v6), *Macaca mulatta* (AF-A0A5F8AVE4-F1-v6), *Callithrix jacchus* (AF-F7IQ82-F1-v6), *M. musculus* (AF-Q9CXR1-F1-v6), *R. norvegicus* (AF-D4A0T8-F1-v6), *Mustela putorius furo* (AF-M3Y4J4-F1-v6), *Equus caballus* (AF-F6ZYE1-F1-v6), *Sus scrofa* (AF-A0A4X1W7G7-F1-v6), *B. taurus* (AF-Q24K14-F1-v6), *Bos indicus* (AF-A0A6P5CGV6-F1-v6), *Bos mutus* (AF-L8IVK0-F1-v6) and *Myotis brandtii* (AF-S7N8T4-F1-v6); avian models comprised *Meleagris gallopavo* (AF-G1NLG0-F1-v6) and *Columba livia* (AF-A0A2I0MNT2-F1-v6); the amphibian model was *Xenopus tropicalis* (AF-Q5FW40-F1-v6); and teleost models comprised *D. rerio* (AF-Q0P3U1-F1-v6), *Oryzias latipes* (AF-H2MM26-F1-v6), *Oryzias melastigma* (AF-A0A3B3D589-F1-v6), *Nothobranchius furzeri* (AF-A0A1A8UQ61-F1-v6) and *Nothobranchius pienaari* (AF-A0A1A8MMP9-F1-v6). Models ranged in size from 262 to 340 residues; *C. livia* (262 amino acids) and *M. brandtii* (289 amino acids) are partial gene models lacking the complete N-terminal region, whereas all remaining orthologues were full-length (334–340 amino acids). The PDB files are superimposed in UCSF chimera (1.18)^89^ using MatchMaker structural comparison tool with default settings^90^; simultaneously fetching the structures and running Needleman-Wunsch alignment and homology matching algorithm with best-aligning pair of chains^91,92^. The parameters for secondary-structure scoring included at 30% weight (secondary structure computed where absent; helix, strand and coil gap-opening penalties of 18, 18 and 6, gap-extension penalty 1), and iterative pruning of atom pairs until none exceeded 2.0 Å, fitting one α-carbon per residue with the human DHRS7 (AF-Q9Y394-F1-v6) as reference. The resulting DHRS7 superimposed model (Extended Data Fig. 2c) was visually processed in UCSF Chimera (1.18).

The predicted structure of *Hs*DHRS7 was obtained from AlphaFold2 (ID:AF-Q9Y394-F1-v6), and the PDB file was used for membrane protein system assembly, and six steps were followed to assemble the hypothetical model using CHARMM36m and CHARMM-GUI Membrane Builder (v3.7)^93^. The AF2 pdb structure was first converted to CHARMM topology with standard charged termini and the default protonation states for physiological pH (~7.4), giving a net protein charge of +4. The protein orientation within the bilayer is determined using PPM 2.0^94^. The system size for lipid bilayer was projected to heterogeneous lipid composition resembling that of endoplasmic reticulum membranes^95^. The bilayer was symmetrical, with an identical lipid complement in each leaflet (154 lipids per leaflet, 308 in total). Phosphatidylcholine was the dominant class (59.1 mol %), comprising 1-stearoyl-2-oleoyl-PC (SOPC, 18:0/18:1; 40 lipids per leaflet, 26.0 mol %), 1-palmitoyl-2-linoleoyl-PC (PLPC, 16:0/18:2; 27 lipids, 17.5 mol %) and 1-palmitoyl-2-oleoyl-PC (POPC, 16:0/18:1; 24 lipids, 15.6 mol %). Phosphatidylethanolamine accounted for a further 22.1 mol %, as 1-palmitoyl-2-stearoyl-PE (PSPE, 16:0/18:0; 12 lipids, 7.8 mol %), 1-stearoyl-2-oleoyl-PE (SOPE, 18:0/18:1; 10 lipids, 6.5 mol %), 1-palmitoyl-2-oleoyl-PE (POPE, 16:0/18:1; 8 lipids, 5.2 mol %) and 1-stearoyl-2-linoleoyl-PE (SLPE, 18:0/18:2; 4 lipids, 2.6 mol %). The remaining lipids were cholesterol (7 lipids, 4.5 mol %), *N*-palmitoyl-sphingomyelin (PSM, 16:0; 6 lipids, 3.9 mol %), 1-oleoyl-2-linoleoyl-phosphatidylserine (OLPS, 18:1/18:2; 5 lipids, 3.2 mol %), 1-stearoyl-2-arachidonoyl-phosphatidylinositol (SAPI, 18:0/20:4; 5 lipids, 3.2 mol %), 1-stearoyl-2-linoleoyl-phosphatidylinositol (SLPI, 18:0/18:2; 4 lipids, 2.6 mol %) and 1-palmitoyl-2-oleoyl-phosphatidic acid (POPA, 16:0/18:1; 2 lipids, 1.3 mol %), giving 5.8 mol % total phosphatidylinositol. In brief, grouped by lipid headgroup, each leaflet contained 59.1 mol % phosphatidylcholine (PC), 22.1 mol % phosphatidylethanolamine (PE), 5.8 mol % phosphatidylinositol (PI), 4.5 mol % cholesterol, 3.9 mol % sphingomyelin (SM), 3.2 mol% phosphatidylserine (PS) and 1.3 mol % phosphatidic acid (PA). The environment was hydrated with TIP3P water molecules, added as a 22.5 Å water slab beyond the protein on each side of the bilayer. Counter-ions were added to neutralize the system and to reproduce a physiological ionic strength of 0.15 M KCl, with ion positions generated by a Monte Carlo procedure using a 20 Å ion-exclusion zone centred on the membrane to prevent ion insertion into the hydrophobic core. The final system contained 92 K^+^ and 64 Cl^−^ ions (156 ions in total). The bilayer lateral dimensions were determined from the number of lipids per leaflet after correcting for the protein cross-sectional area (59 × 50 × 77 Å), comprising of rectangular unit cell 101.04 × 101.04 × 124.79 Å. The assembled model (Extended Data Fig. 2d) is visually processed in UCSF Chimera (1.18) and rendered in UCSF ChimeraX (1.10.1).

### Protein sequence alignment of DHRS7 orthologues and secondary structure distribution

The AlphaFold-predicted structures of DHRS7 orthologues from *M. musculus* (AlphaFold DB:Q9CXR1, AF-Q9CXR1-F1-v6), *R. norvegicus* (AlphaFold DB:D4A0T8, AF-D4A0T8-F1-v6), *B. taurus* (AlphaFold DB:Q24K14, AF-Q24K14-F1-v6), *D. rerio* (AlphaFold DB: Q0P3U1, AF-Q0P3U1-F1-v6) and *H. sapiens* (AlphaFold DB: Q9Y394, AF-Q9Y394-F1-v6) were retrieved from a pipeline implemented in Python (v3.10.20). The DHRS7 orthologues were retrieved from Ensembl (release 116)^96^ through its REST interface using the requests library (v2.33.1). The one2one and one2many 253 orthologues were sampled across vertebrates. 187 orthologues within ±30 amino acids of the human reference length were aligned with MUSCLE (v5.3. build d9725ac)^97,98^. The AlphaFold-predicted structure of human DHRS7 (UniProt Q9Y394; model AF-Q9Y394-F1) was used to generate molecular surface from the atomic coordinates using a 1.4 Å probe radius, corresponding to a water-sized solvent probe. residues of interest were highlighted in yellow with 4.0 Å mapping radius, with the remaining DHRS7 surface rendered in grey molecular surface and molecular surface slice (Extended Data Fig. 5a) in UCSF Chimera (v1.18). Residues were selected from the cross-species MUSCLE alignment described above as positions at which *H. sapiens*, *D. rerio* and *B. taurus* DHRS7 share an identical residue that is substituted in both rodents (*M. musculus* and *R. norvegicus*). The human reference positions map directly onto the model. These 15 positions (human numbering)—L18, C147, D149, E160, P198, R218, S230, I232, C233, F291, W306, N310, G323, V324 and A326—were coloured yellow on the molecular surface of *Hs*DHRS7. Per-species the secondary structure was assigned from the AlphaFold predicted structure models and overlayed on MUSCLE alignment, the secondary structure relies on backbone *φ*–*ψ* torsion angles of the model coordinates from AlphaFold. Conservation followed the Clustal convention and columns were scored by exact residue identity across the select species (Extended Data Fig. 5b).

DHRS7 orthologue protein sequence analysis and multiple-sequence alignment was performed with Python (v3.10.20). Sequence alignment and input/output and pairwise sequence-identity calculations were performed with Biopython (v1.87)^99^, tabular data were handled with pandas (v2.3.3.)^100^, numerical operations with NumPy (v1.24.4)^81^ and auxiliary panels were rendered with Matplotlib (v3.10.8)^85^. Sequences were aligned under default parameters (standard affine gap penalties) and pairwise identities to the human reference were derived from global (Needleman-Wunsch), BLOSUM62 alignments (gap-opening, −10; gap-extension, −0.5). The environment (Python v3.10.20) is provided as bundle.yml and multiple species sequence alignment output with protein.pdb model and the Extended Data Fig. 5b reproduction pipeline is available on GitHub (https://github.com/zazadovv/Redox-DHRS7_subcellular.git).

### Image analysis

#### Zebrafish experiments. Sudan Black.

Image stacks were imported into ImageJ and used to generate minimum intensity projections. Neutrophils were counted manually in the intestine area.

#### Dextran gavage.

Image stacks were imported into ImageJ and used to generate maximum-intensity projections. The projections were background subtracted and rectangular regions of interests were applied ~30–50 μm from the intestine with the longer side of the rectangle parallel to the axis of the intestine. Results are given as mean fluorescence intensity in the region of interest as quantified in Fiji.

#### Active caspase-3 immunostaining.

Transmitted light image stacks were imported into Fiji and used to generate minimum intensity projections, which were used to label the intestine as a region of interest for each fish. Image stacks of the active caspase-3 signal (Alexa 488 or Alexa555) were imported into ImageJ and used to generate maximum-intensity projections. The projected images were then background subtracted. Images were thresholded using the MinError method in Image J, converted to binary images and segmented using the watershed algorithm in Fiji. Particles smaller than 5 μm^2^ were omitted from the analysis. Results were given as percentage positive area of the total intestinal area.

#### Caco-2 cell experiments.

##### NUDT1.

The abundance of the fluorescent signal was determined as the mean fluorescence intensity of the whole background subtracted image. Background was determined using images of cells which were not stained with the primary antibody. Results are given as a percentage of mean fluorescence intensity compared to the mean of respective experimental control (Vehicle treated NTC or siControl cells in Fig. 5c and Extended Data Fig. 10g–i, siControl or NTC cells in Extended Data Fig. 10e,f).

##### DAPI.

Images were background subtracted, thresholded using the ‘Huang dark’ method, converted to binary images and segmented using the Watershed algorithm in Fiji. Particles smaller than 25 μm^2^ were omitted from the analysis.

##### Active caspase-3.

Images were background subtracted, thresholded using the MinError method, converted to binary images and segmented using the watershed algorithm in Fiji. Particles smaller than 5 μm^2^ were omitted from the analysis. Results are given as the number of active caspase-3^+^ particles normalized to DAPI^+^ particles (similar results were obtained using the number of active caspase-3^+^ particles or percentage of total active caspase-3^+^ area).

### Statistics

No statistical methods were used to determine the sample size, which aligns with those reported in previous publications^10,11^. Statistical analyses were performed by using GraphPad Prism 8 (v8.3.0) or MATLAB 2024b (update 4,24.2.0.2833386, 64-bit, win64, MathWorks). Outliers were identified and removed using the Rout method in Prism. Shapiro–Wilk test was used for measuring normal distribution for each dataset. Only if the data were normally distributed, a parametric unpaired two-tailed Welch’s *t*-test was applied to the dataset. If the data were not normally distributed, nonparametric unpaired two-tailed Mann–Whitney *U* test was used. For datasets with small sizes (*n* = 3 or 4), Welch’s *t*-test was used even when the Shapiro–Wilk test indicates a departure from normality (Fig. 1c, right and Fig. 2c, right), because such small sample sizes are insufficient for reliable assessment of the underlying distribution. For multiple comparisons testing of normally distributed data ordinary one-way ANOVA followed by post hoc testing (uncorrected Fisher’s least significant difference post hoc test or Tukey’s post hoc test for comparing all experimental groups and Dunnett’s multiple comparisons test for comparing experimental groups to common control groups) were used. For normally distributed data with unequal variances, the Brown–Forsythe ANOVA was used followed by Dunnett’s T3 multiple comparisons test. For multiple comparison of non-normally distributed data, the Kruskal–Wallis ANOVA followed by Dunn’s post hoc test was used. *P* values < 0.05 were considered statistically significant. Exact *P* values for Welch’s *t*-test and Mann–Whitney *U* test reported by prism as *P* < 0.0001 were independently calculated using the Real Statistics Resource Pack for Microsoft Excel (v9.9, C. Zaiontz). For ANOVA based multiple-comparison tests, *P* values reported by Prism as *P* < 0.0001 are presented as such because exact numerical *P* values were not provided by the software. For adult zebrafish tissue lipidomics, sex-associated effects were assessed for each lipid in 12 independent biological samples (*n* = 3 females and *n* = 3 males per genotype) using an exact, two-sided balanced permutation test. Concentrations were log_2_-transformed after addition of a lipid-specific pseudocount equal to one-half of the smallest positive concentration. Sex labels were permuted within each genotype while retaining three females and three males, producing 20 possible assignments per genotype and 400 combined permutations. The overall sex effect was defined as the average within-genotype male-minus-female contrast, and the genotype-by-sex interaction as the difference between the mutant and wild-type sex contrasts. Benjamini–Hochberg correction was applied separately across the 84 lipid-specific *P* values for the overall sex effect and genotype-by-sex interaction analyses; an adjusted *q* value < 0.05 was considered significant. For genotype comparisons, six independent biological samples per genotype (three females and three males) were analysed. Normality was assessed separately within each genotype using the Shapiro–Wilk test. When both groups were consistent with normality (*P* > 0.05), a two-sided Welch’s *t*-test was used; otherwise, a two-sided Mann–Whitney *U* test was applied. Fold change was calculated from untransformed concentrations as the mutant group mean divided by the wild-type group mean. Lipids with a nominal genotype-comparison *P* < 0.05 were included in the heat map and ordered by decreasing fold change. All error bars represent the s.d. Detailed sample sizes are listed in the figure legends. Animal experiments conducted on different days were aggregated. Experiments were not randomized, and investigators were not blinded to allocation during experiments or outcome assessment. For each treatment, both treated and control embryos were sourced from the same egg spawn. Animals were not reused across experiments. Larvae were selected based on normal morphology, a beating heart, and circulating red blood cells. All images in the figures represent typical phenotypes and expression patterns for their respective conditions. All detailed statistical tests are presented in the Source Data.

## Extended Data

**Extended Data Fig. 1 |:**
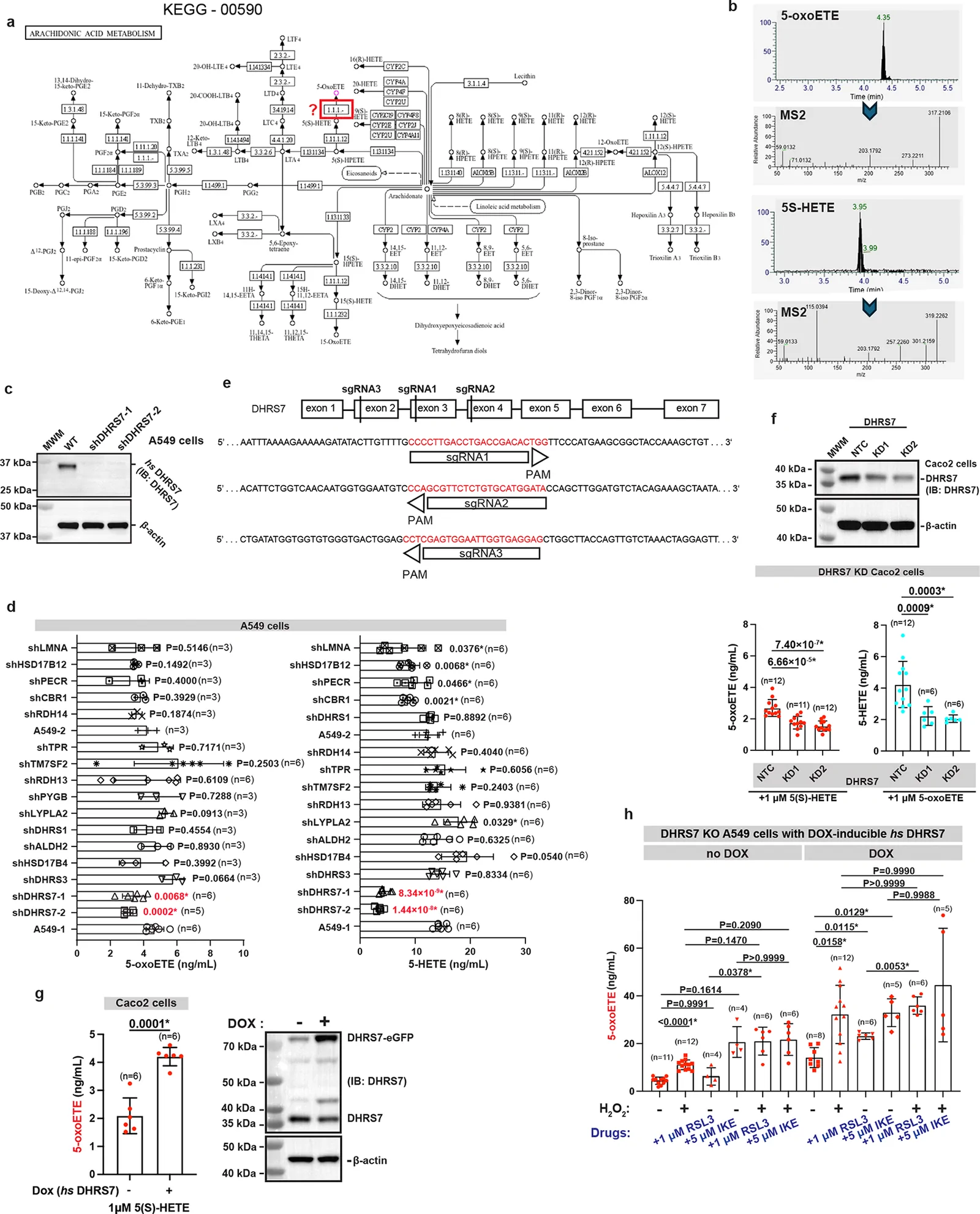
5-HEDH candidate screen, related to Fig. 1. (**a**) KEGG arachidonic acid pathway showing the position of 5-HEDH (red). Reproduced from https://www.kegg.jp/pathway/map00590 (ref. 102) CC BY 4.0. (**b**) LC chromatograms and MS/MS spectra of 5-oxoETE and 5-HETE. (**c**) Western blot confirming DHRS7 knockdown by the two shRNAs (shDHRS7–1 and shDHRS7–2) used in the screen. β-actin, loading control. MWM, molecular weight marker. (**d**) Limited candidate screen for 5-HEDH using shRNA interference and LC-MS in A549 cells. Left, 5-oxoETE production: shDHRS7–1, shDHRS7–2, shDHRS3, shHSD17B4, shALDH2, shDHRS1, shLYPLA2, shPYGB, shRDH13, shTM7SF2, shTPR were compared to A549–1; shRDH14, shCBR1, shPECR, shHSD17B12, shLMNA were compared to A549–2. Right, 5-HETE production: shDHRS7–1, shDHRS7–2, shDHRS3, shHSD17B4, shALDH2, shLYPLA2, shRDH13, shTM7SF2, shTPR, shRDH14 were compared to A549–1; shDHRS1, shCBR1, shPECR, shHSD17B12, shLMNA were compared to A549–2. Two tailed welch’s t test or Mann-Whitney U test was used based on data normality by the Shapiro-Wilk test. (**e**) DHRS7 CRISPR design with three sgRNAs targeting exons 2, 3 and 4. (**f**) Caco-2 DHRS7 knockdown (KD) bulk populations generated with sgRNA1 (KD1) or sgRNA3 (KD2). Top, western blot confirming DHRS7 knockdown. MWM, molecular weight marker. Bottom, LC-MS of Caco-2 DHRS7-KD cells treated with 1 μM 5(S)-HETE (left), or 1 μM 5-oxoETE (right). P values: left, unpaired, nonparametric, two tailed Mann-Whitney Test; right, unpaired, parametric, two tailed Welch’s t test. (**g**) Left, LC-MS of Caco-2 cells ± *hs* DHRS7 overexpression (OE) treated with 1 μM 5(S)-HETE. P values, unpaired, parametric, two tailed Welch’s t test. Right, western blot of endogenous DHRS7 and *hs* DHRS7-eGFP in *wt* and DHRS7 OE cells following induction with 1 μg/mL doxycycline (DOX). β-actin, loading control. MWM, molecular weight marker. (**h**) LC-MS measurement of 5-oxoETE under the indicated conditions. P values, one-way ANOVA (Brown-Forsythe and Welch ANOVA tests). Bars indicate mean ±SD. IB, immunoblot. Parentheses indicate the number of biological replicates, each derived from an independent petri dish culture. Asterisks indicate significant differences versus respective controls (p < 0.05). LC-MS measurements were performed once with the indicated number of biologically independent replicates, each from an independent petri dish culture; absolute lipid concentrations were not compared across extraction batches; each analytical batch was processed together with its matched wild-type controls (see Methods).

**Extended Data Fig. 2 |:**
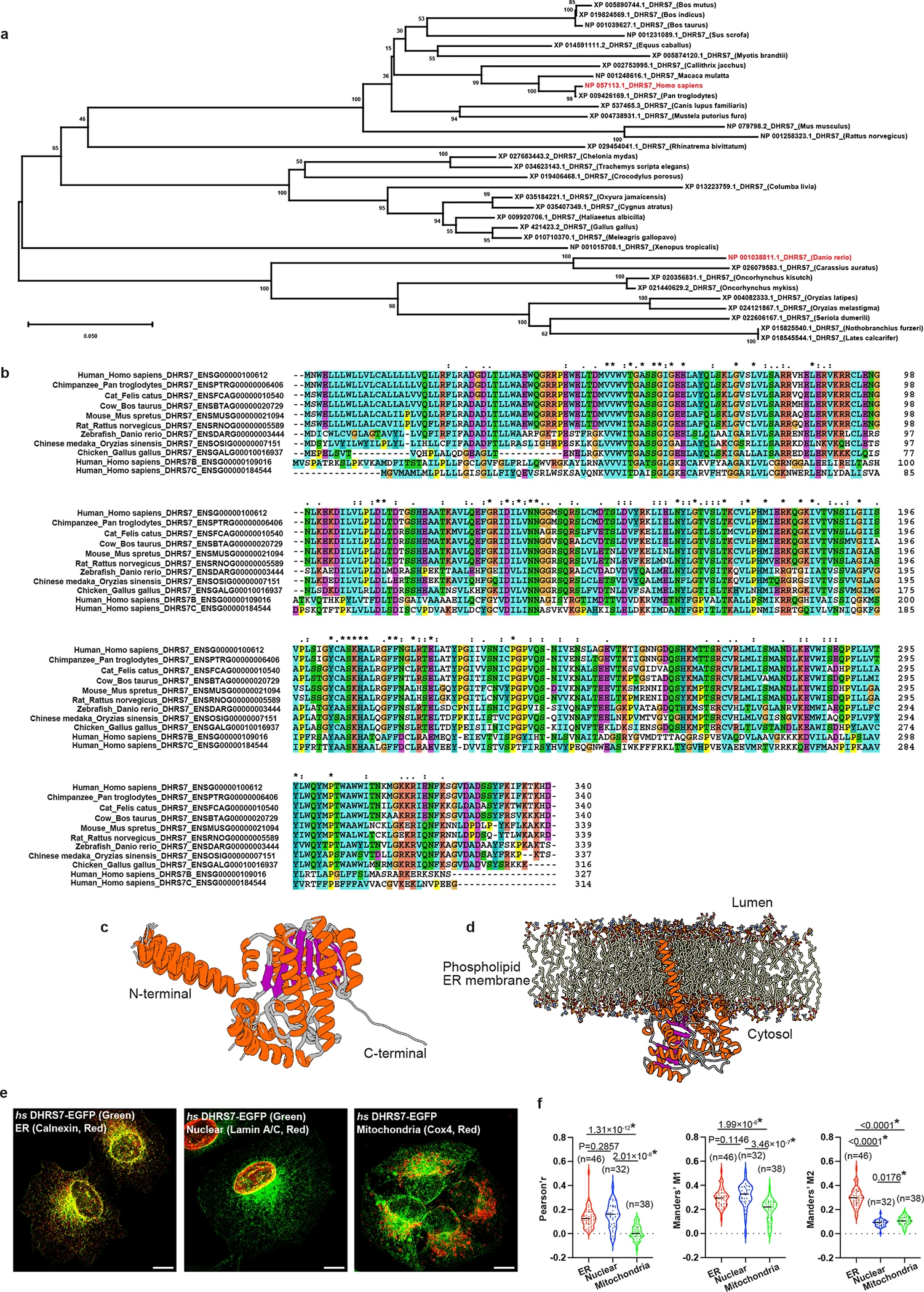
Phylogenetic analysis of DHRS7, related to Fig. 1. (**a**) Phylogenetic tree analysis of DHRS7 including human (*homo sapiens*), zebrafish (*danio rerio*) and 32 other species. (**b**) Protein sequence alignment of human DHRS7, DHRS7B, DHRS7C, zebrafish DHRS7 and orthologs from 7 other species. Asterisks (*), fully conserved residues. Colons (:) strongly similar conserved residues. Periods (.), weakly similar conserved residues. (**c**) Superimposed AlphaFold2 structures of 22 selected DHRS7 orthologs from the 34 species shown in Extended Data Fig. 2a. The colour key corresponds to alpha-helices (orange), beta-sheets (purple) and loops or unstructured (grey). (**d**) Cartoon model depicting the predicted structure of DHRS7 (ID:AF-Q9Y394-F1-v4), anchored in simulated ER phospholipid membrane leaflets. (**e**) Representative images of co-localization of DHRS7 with the Endoplasmic Reticulum (ER), Nucleus and Mitochondria via immunofluorescence staining. Green, DHRS7 overexpression protein. Red in left panel, ER staining using an ER-specific antibody, anti-Calnexin. Red in middle panel, nuclear staining with a nuclear-specific antibody, anti-Lamin A/C. Red in right panel, mitochondria staining with a mitochondria specific antibody, anti-Cox4. Scale bar, 10 μm. (**f**) Quantification of co-localization parameters from (e). Pearson’s r indicates the overall correlation of fluorescence intensity between two channels. Manders’ M1 represents the fraction of the *hs* DHRS7-eGFP signal overlapping with Calnexin, Lamin A/C, or COX4. Manders’ M2 denotes the fraction of the Calnexin, Lamin A/C, or COX4 signal overlapping with *hs* DHRS7-eGFP. P values were calculated using two tailed Welch’s t-test or Mann-Whitney U test, depending on data normality by Shapiro-Wilk test. Parentheses indicate the number of fields of view analyzed from four different wells per condition across two independent experiments (FOVs = 46 for ER, FOVs = 32 for Nuclear, FOVs = 38 for Mitochondria). FOVs, fields of views.

**Extended Data Fig. 3 |:**
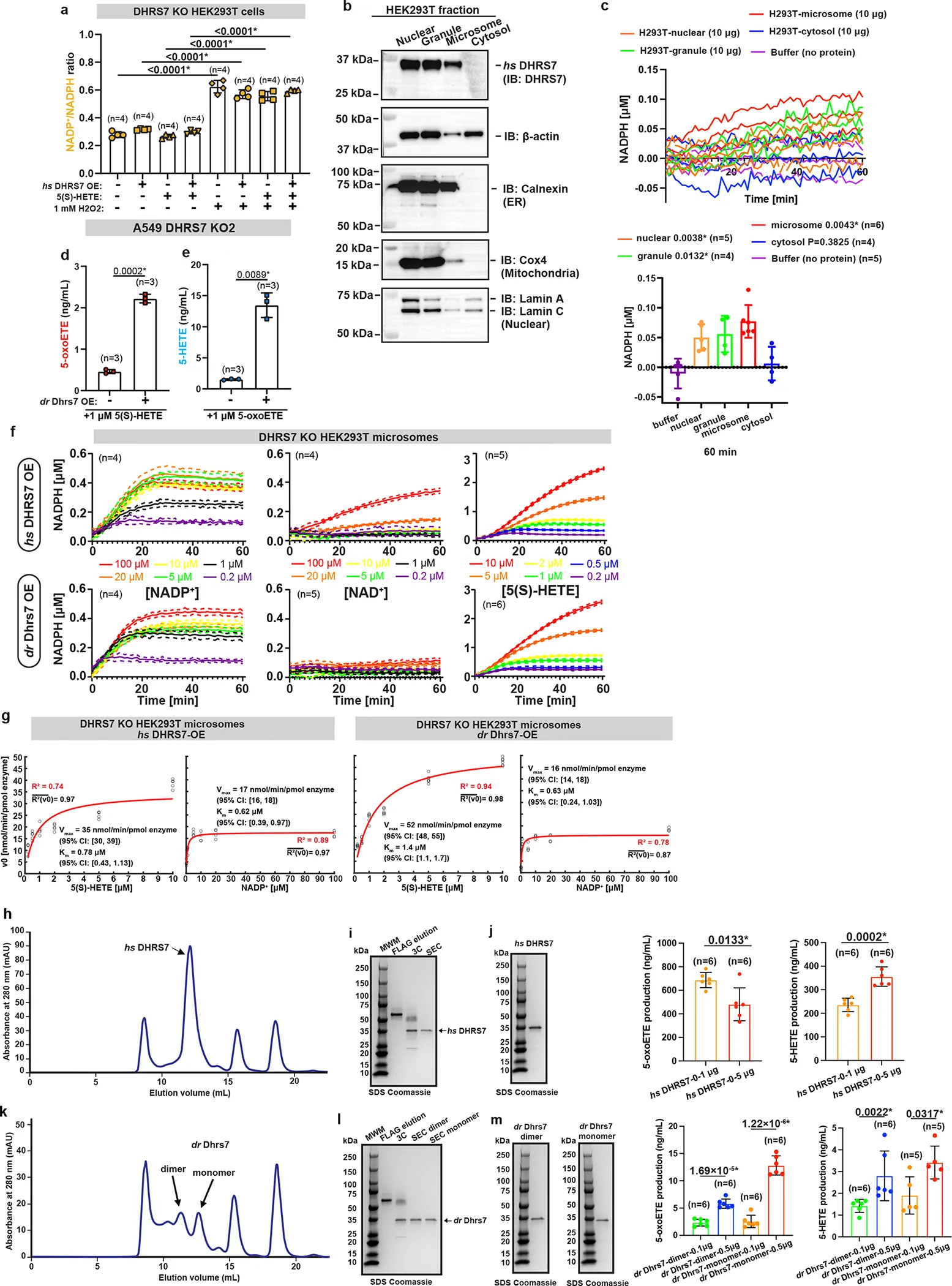
Characterization of DHRS7 as 5-HEDH, related to Figs. 1–3. (**a**) NADP^+^/NADPH ratios in HEK293T DHRS7 KO1 cells ± *hs* DHRS7 OE, 1 μM 5(S)-HETE, or 1 mM H_2_O_2_ (20 min). P values, one-way ANOVA (uncorrected Fisher’s LSD test, two-tailed). Parentheses indicate the number of biological replicates from independent wells. (**b**) Western blot of endogenous DHRS7 in nuclear, granule, microsome, cytosolic fractions of HEK293T cells. β-actin, loading control. Calnexin, Cox4 and Lamin A/C were as markers for the ER, mitochondria and nuclear, respectively. (**c**) NADPH production assays for 5-HEDH activity in nuclear, granule, microsome and cytosolic fractions with 1 μM 5(S)-HETE and 100 μM NADP^+^ (top). NADPH levels at 60 min are shown below. P values at 60 min: microsomes, unpaired, nonparametric two tailed Mann-Whitney test; other fractions, unpaired, parametric, two tailed, Welch’s t-test. Border plot regions, SD. Parentheses indicate the number of biological replicates, each derived from an independent well containing the indicated cell fractions. (**d-e**) LC-MS assays of 5-oxoETE or 5-HETE production in DHRS7-KO2 A549 cells ± *dr* Dhrs7-OE and lipid substrate incubation as indicated. Dhrs7 overexpression was induced with 1 μg/mL doxycycline. P values, unpaired, parametric, two tailed Welch’s t test. Error bars, SD. Parentheses indicate the number of biological replicates from independent petri dish cultures. (**f**) NADPH (or NADH) kinetics with 1.0975 pmol microsomal *hs* DHRS7 (top) or *dr* Dhrs7 (bottom) at the indicated, variable concentrations of NADP^+^ (left), NAD^+^ (middle), or 5(S)-HETE (right), with 1 μM 5(S)-HETE (left-middle), or 100 μM NADP^+^ (right), respectively. The v_0_ approximation of Extended Data Fig. 3g is based on the first 20 timepoints of the data in the left and right panels. Solid lines, mean values of data; dotted lines, SD. Parentheses indicate the number of biological replicates, each derived from an independent well containing microsomes. (**g**) The pseudo-first order Michaelis-Menten reaction parameters were determined by first approximating the initial reaction velocities (v_0_) from the experimentally derived NADPH(5(S)-HETE, t) and NADPH(NADP^+^, t) isotherms by linear fitting their first 20 timepoints. To determine the NADPH(t) isotherms, either one of the substrates of the 5-HEDH dehydrogenase reaction (5(S)-HETE, NADP^+^) was provided in excess while the concentration of the other was altered. [NADPH] was measured using a plate reader assay as a function of time (t). Next, a standard Michaelis-Menten model (red line) was fitted to the derived v_0_(5(S)-HETE) *(subpanel-left*) and v_0_(NADP^+^) (*subpanel-right*) isotherms to approximate the pseudo-first order kinetic parameters of the 5-HEDH dehydrogenase reaction. K_m_, Michaelis constant. V_max_, maximum velocity. R^2^(v_0_), average goodness of linear fit for the v_0_ approximation. R^2^, goodness of fit Michaelis-Menten model fit. Brackets, 95% confidence interval (CI). Left subpanel, *hs* DHRS7. Right subpanel, *dr* Dhrs7. Error bars, SD. See Methods for further details. (**h**,**k**) Size-exclusion chromatography elution profiles of *hs* DHRS7 and *dr* Dhrs7. (**i**,**l**) Coomassie-stained SDS-PAGE gel showing the protein purification steps, including samples collected after FLAG elution, 3 C protease cleavage, and size-exclusion chromatography. (**j**,**m**) Left, Coomassie-stained SDS-PAGE gel showing *hs* DHRS7 and *dr* Dhrs7 proteins. Right, LC-MS assays of 5-oxoETE or 5-HETE production by purified *hs* DHRS7 and *dr* Dhrs7 proteins incubated with either 1 μM 5(S)-HETE or 1 μM 5-oxoETE in the presence of with 100 μM NADP^+^ or 100 μM NADPH. Parentheses indicate biological replicates from independent tubes. P values: j and m (left), unpaired, parametric, two tailed Welch’s t test; m (right), unpaired, nonparametric, two tailed Mann-Whitney Test. IB, immunoblot. Asterisks indicate significant differences versus respective controls (p < 0.05). Enzymatic assays were performed once with the indicated number of biologically independent replicates, each derived from an independent well or tube of microsomes.

**Extended Data Fig. 4 |:**
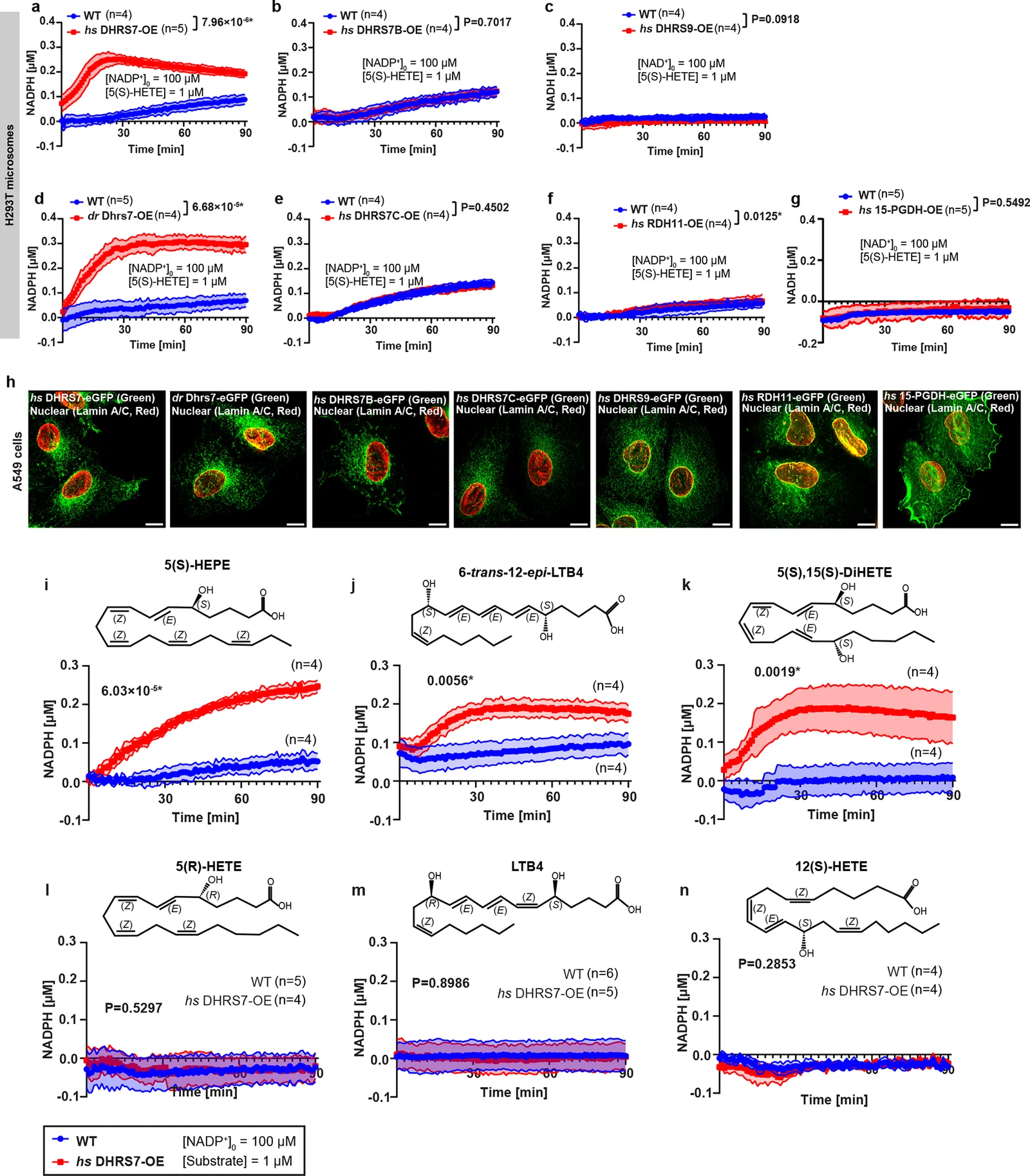
Characterization of DHRS7 as 5-HEDH, related to Figs. 1–3. (**a-g**) NADPH or NADH production assays with *wt* HEK293T microsomes overexpressing (**a**) *hs* DHRS7, (**b**) *hs* DHRS7B, (**c**) *hs* DHRS9, (**d**) *dr* Dhrs7, (**e**) *hs* DHRS7C, (**f**) *hs* RDH11, or (**g**) *hs* 15-PGDH. 10 μg microsomes were incubated with 1 μM 5(S)-HETE and 100 μM NADP^+^, except for DHRS9 and 15-PGDH expressing microsomes, which were incubated with 1 μM 5(S)-HETE and 100 μM NAD^+^. NADPH or NADH levels are background corrected (using DMSO as vehicle control without substrate). P values, unpaired, parametric, two-tailed, Welch’s t test at the 20 min timepoint. Shaded plot regions, SD. Parentheses indicate the number of biological replicates, each derived from an independent well containing microsomes. (**h**) Representative images of the sub-cellular localization for the indicated eGFP fusion proteins in A549 cells. Scale bar, 10 μm. Green, fusion protein. Red, immunofluorescence staining of Lamin A/C marking the nuclei. FOVs=11 for *hs* DHRS7B, FOVs=10 for *hs* DHRS7C, FOVs=10 for *hs* DHRS9, FOVs=14 for *hs* RDH11, FOVs=11 for *hs* 15-PGDH, from two different wells. (**i-n**) NADPH production assays were conducted with 100 μM NADP^+^ and 1 μM 5(S)-HEPE (**i**), 6-*trans*-12-*epi* LTB_4_ (**j**), 5(S),15(S)-DiHETE (**k**), 5(R)-HETE (**l**), LTB_4_ (**m**), and 12(S)-HETE (**n**), FOVs, fields of view. NADPH levels are background corrected (using DMSO as vehicle control without substrate). P values, unpaired, parametric, two tailed, Welch’s t test at the 20 min timepoint. Shaded plot regions, SD. Asterisks, highlight significant changes as compared to respective control (p < 0.05). Parentheses indicate the number of biological replicates, each derived from an independent well containing microsomes. Enzymatic assays were performed once with the indicated number of biologically independent replicates, each derived from an independent well or tube of microsomes.

**Extended Data Fig. 5 |:**
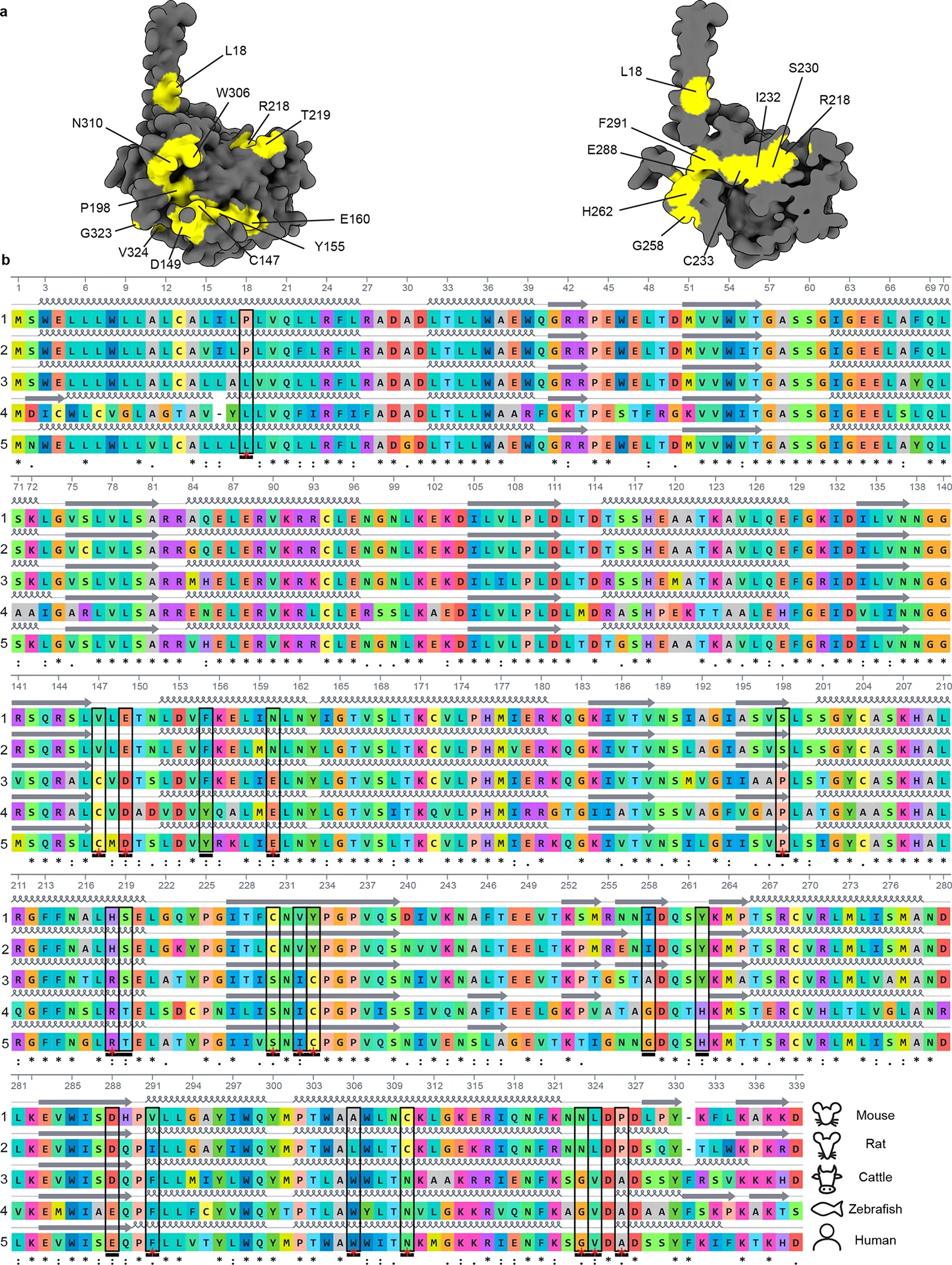
Structural comparison and sequence alignment of DHRS7 orthologs from *Mus musculus*, *Rattus norvegicus*, *Bos taurus*, *Danio rerio* and *Homo sapiens*, related to Fig. 2. (**a**) AlphaFold2-predicted human DHRS7 structure shown as a molecular surface. The yellow surface patches indicate mapped residues on the molecular surface of *hs* DHRS7 (AlphaFold ID: AF-Q9Y394-F1; UniProt: Q9Y394) where sites with conservation in human and zebrafish and divergence in other species are highlighted. The image on the left shows the intact surface rendering, while the right image is the same surface after slicing/cutaway to reveal additional highlighted residues. (**b**) Protein sequences alignment of DHRS7 across five species: mouse (*Mus musculus*, row 1), rat (*Rattus norvegicus, AF-*D4A0T8-*F1*, row 2), cattle (*Bos taurus*, AF-Q24K14-F1, row 3), zebrafish (*Danio rerio*, AF-Q0P3U1-F1, row 4) and human (*Homo sapiens*, AF-Q9Y394-F1 row 5) indexes along the human reference sequence position (Residue 1–339). Residues are colored by amino acid. Grey glyphs above each row show the secondary structure of that species AlphaFold model (α-helices as coils, β-strands as arrows, loops as lines). Beneath each block, the conservation line marks positions that are identical (*), strongly similar (:) or weakly similar (.) across the five species. Black frames outline columns in which human and zebrafish carry the same residue while both rodent orthologs, mouse and rat, differ. Red asterisks mark the subset of these positions that are also conserved in cattle, indicating conservation identity of residues across human, zebrafish, and cattle with divergence in both rodents. This figure presents computational structural predictions and sequence alignments; no experimental replicates apply.

**Extended Data Fig. 6 |:**
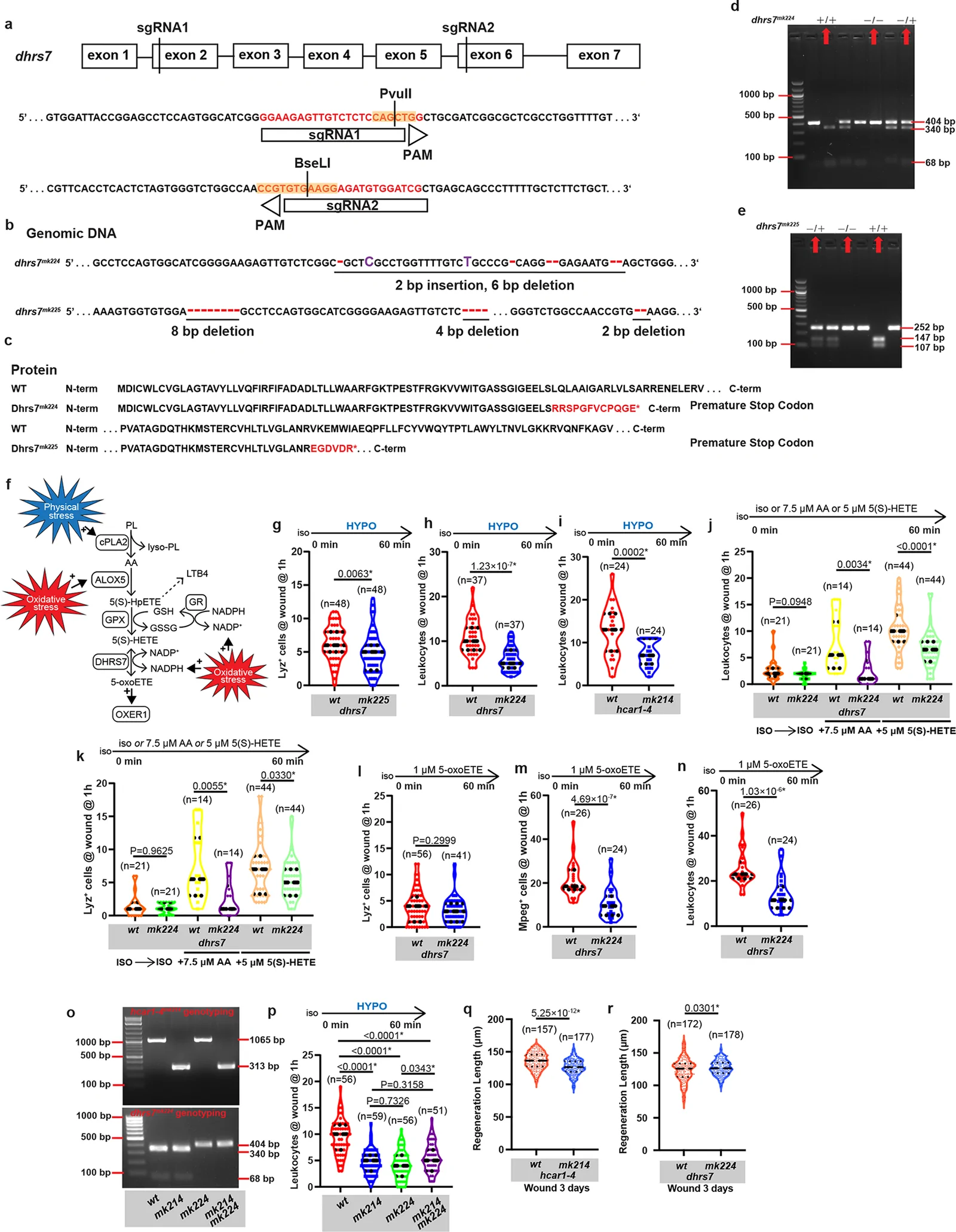
Generation and functional characterization of *dhrs7* mutant zebrafish, related to Fig. 3. (**a**) CRISPR design for *dhrs7* mutant zebrafish using single guide RNAs (sgRNAs). For *dhrs7*^*mk224/mk224*^, sgRNA1 was designed to target exon 2. Target disruption is predicted to occur at a PvuII restriction site (CAGĈTG) (highlighted by orange text) in the genomic DNA (deoxyribonucleic acid) sequence. For *dhrs7*^*mk225/mk225*^, two different sgRNAs were used together. Specifically, sgRNA2 was designed to target exon 6. Target disruption is predicted to occur at a BseLI restriction site (CCGTGTGÂAGG) (highlighted by orange text) in the genomic DNA sequence. (**b**) *dhrs7* mutant alleles. The mutant *dhrs7*^*mk224/mk224*^ allele was generated by sgRNA1 and has a 4-base pair (bp) deletion resulting in a frameshift and the destruction of PvuII restriction site. The mutant *dhrs7*^*mk225/mk225*^ allele was generated by co-injection of sgRNA1 and sgRNA2 and has a 12 bp deletion leading to the destruction of a PvuII restriction site, as well as a 2 bp deletion and the destruction of a BseLI restriction site, resulting in a frame shift. Purple text, mutant sequence (insertion) that differs from the *wt* sequence. Red text, deletion sequence that differs from the *wt* sequence. (**c**) The mutant *dhrs7*^*mk224*^ and *dhrs7*^*mk225*^ alleles both have premature stop codons in the coding sequence. Red text, mutant *dhrs7*^*mk224*^ and *dhrs7*^*mk225*^ sequences that differs from the *wt* sequence. (**d-e**) Genotyping of progeny from heterozygous *dhrs7*^*mk224*^ or *dhrs7*^*mk225*^ zebrafish crosses by PCR, restriction digestion and agarose gel electrophoresis. For *dhrs7*^*mk224/mk224*^ (**d**), PvuII-digestion yields 340 bp and 68 bp products for WT, a 404-bp undigested product for homozygous mutants, and all three products for heterozygotes. For *dhrs7*^*mk225/mk225*^ (**e**), BseLI digestion yields 147- and 107-bp products for WT, a 252-bp undigested product for homozygous mutants, and all three products for heterozygotes. (**f**) Oxoeicosanoid pathway. cPla2 is activated by physical stress (specifically, osmotic nuclear deformation) to release arachidonic acid (AA) from nuclear membrane phospholipids (PL). ALOX5 (5-lipoxygenase) metabolizes AA into 5-hydroperoxy-eicosatetraenoic acid (5(S)-HpETE). The catalytic site of ALOX5 is primed by free lipid peroxides (i.e., oxidative stress). 5(S)-HpETE may be either used for LTB_4_ production or reduced by a glutathione peroxidase (GPX) into 5-hydroxyeicosatetraenoic acid (5(S)-HETE). The respective oxidized glutathione (GSSG) is converted back to reduced glutathione (GSH) by glutathione reductase (GR) under consumption of NADPH and generation of NADP^+^. Under oxidative (=high NADP^+^) and physical stress (=high 5(S)-HETE), 5-oxoETE is generated from 5(S)-HETE. Under unstressed conditions, the reverse reaction is preferred. 5-oxoETE exerts its physiological effects through the OXER1 receptor (or its zebrafish ortholog Hcar1–4). (**g**) Quantification of Lyz^+^ neutrophils at the wound margins of *dhrs7*^*mk225/mk225*^ or *wt* control siblings at 1 h. P values: unpaired, nonparametric, two tailed Mann-Whitney Test. Numbers in parentheses indicate the number of zebrafish larvae analyzed, pooled from two independent experiments. (**h-i**) Quantification of “generic” leukocytes (identified by transmitted light contrast and motility) at the wound margins of (**h**) *dhrs7*^*mk224/mk224*^ (**i**) *hcar1–4*^*mk214/mk214*^ zebrafish compared to their respective control siblings (same experiment as in Fig. 3c,d). P values: unpaired, nonparametric, two tailed Mann-Whitney Test. Numbers in parentheses indicate the number of zebrafish larvae analyzed, pooled from two independent experiments in h and i. (**j**) Quantification of “generic” leukocytes or (**k**) Lyz^+^ neutrophils at wound margin of *dhrs7*^*mk224/mk224*^ mutant and sibling control larvae treated with the indicated lipids and isotonic bathing solution (Tg(*lyz*:pm2-mKate2) background). Quantification of (**l**) Lyz+ neutrophils, (**m**) Mpeg+ macrophages, or (**n**) “generic” leukocytes at the wound margins of *dhrs7*^*mk224/mk224*^ mutant or *wt* sibling larvae after 1 h of incubation with 1 μM 5-oxoETE in isotonic bathing solution (Tg(*lyz*:pm2-mk2) or Tg(*mpeg1*:eGFP) backgrounds). Note, (m-n) refer to the same experiment. P values were determined using an unpaired, nonparametric, two tailed Mann-Whitney Test. Numbers in parentheses indicate the number of zebrafish larvae analyzed, pooled from two to three independent experiments. (**o**) Genotyping validation of *dhrs7* and *hcar1–4* single or double mutants using agarose gel electrophoresis. (**p**) Quantification of “generic” leukocytes at wound margin of *dhrs7*^*mk224*/*mk224*^ *hcar1–4*^*mk214*/*mk214*^ double mutant, *dhrs7*^*mk224*/*mk224*^ and *hcar1–4*^*mk214*/*mk214*^ single mutants and their *wt* control siblings. Parentheses, total number of wounded larvae. P values, ordinary one-way ANOVA. (**q-r**) Regeneration length of *dhrs7*^*mk224/mk224*^ and *hcar1–4*^*mk214/mk214*^ zebrafish compared to their respective control siblings. P values, unpaired, parametric, two tailed Welch’s t test. Numbers in parentheses indicate the number of zebrafish larvae analyzed, pooled from two independent experiments. Asterisks, highlight significant changes as compared to respective control (p < 0.05). Violin plots show the data distribution; dashed lines indicate the third quartiles (top line), medians (middle line), first quartiles (bottom line).

**Extended Data Fig. 7 |:**
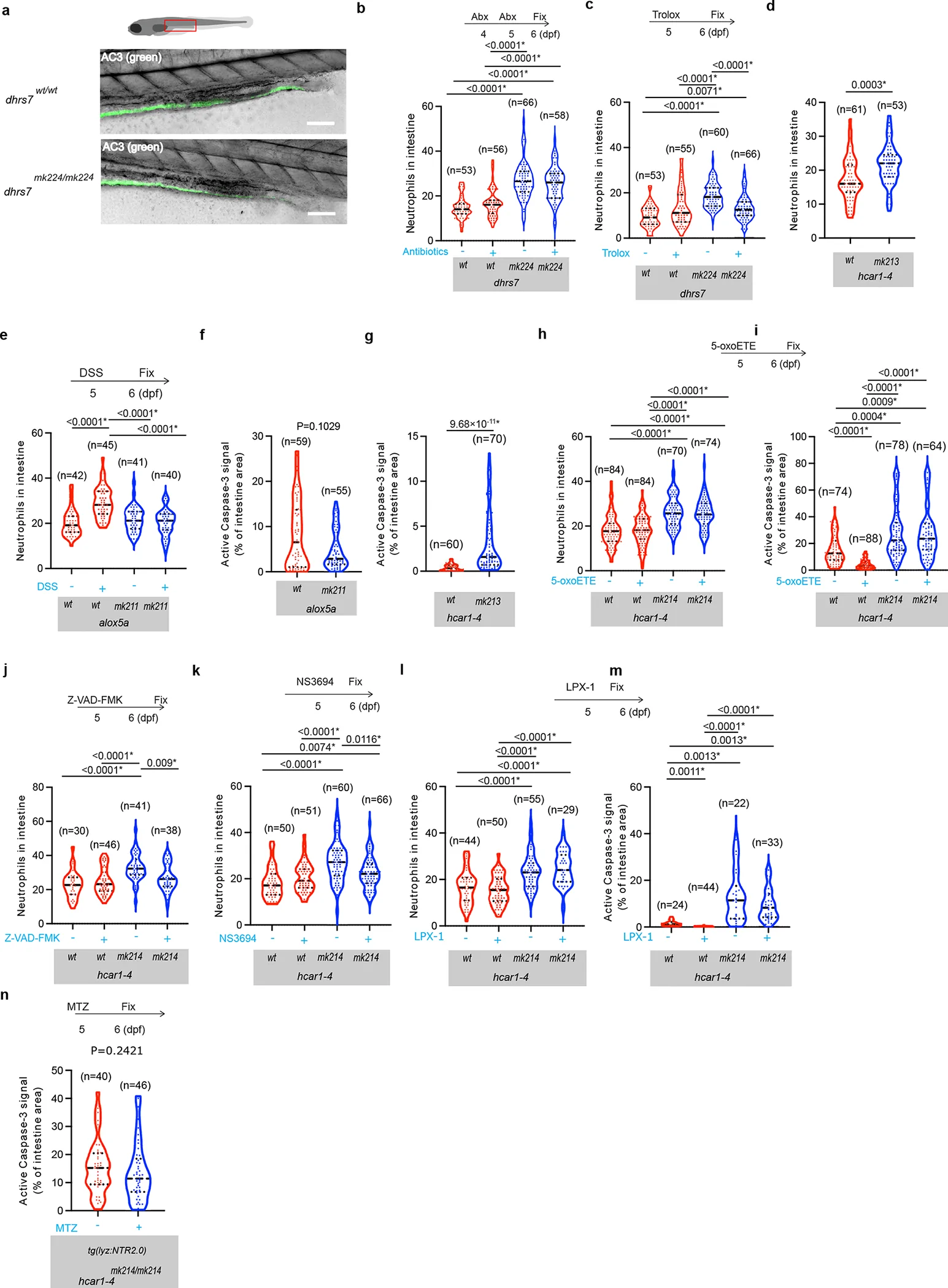
Characterization of Dhrs7 and Hcar1–4 function in live zebrafish, related to Figs. 3 and 4. (**a**) Representative images of active caspase-3 immunostaining (green) of *dhrs7*^*wt*/*wt*^ and *dhrs7*^*mk224*/*mk224*^ larvae. Scale bars, 100 μm. Zebrafish graphic from ref. 51 CC BY 4.0. (**b**) Top: Scheme for antibiotics treatment. Larvae were treated with ampicillin and kanamycin for 48 h. Embryo water was switched to fresh water with new antibiotics after 24 h of treatment. Bottom: Quantification of intestinal neutrophil counts in control and antibiotics treated larvae. (**c**) Top: Experimental scheme for Trolox treatment. Larvae were treated with Trolox or DMSO control for 24 h. Bottom: Quantification of intestinal neutrophil counts in control and Trolox treated *wt* and *dhrs7*^*mk224/mk224*^ larvae. Asterisks, highlight significant changes as compared to respective control (p < 0.05). (**d**) Quantification of intestinal neutrophil counts in 6 dpf *hcar1–4*^*wt*/*wt*^ or *hcar1–4*^mk213/mk213^ larvae. (**e**) Quantification of intestinal neutrophil counts in 6 dpf *alox5a*^wt/wt^ or *alox5a*^mk211/mk211^ larvae after overnight DSS treatment. (**f**) Quantification of intestinal anti-active caspase-3 immunostaining in 6 dpf *alox5a*^wt/wt^ or *alox5a*^mk211/mk211^ larvae. (**g**) Quantification of intestinal anti-active caspase-3 immunostaining in 6 dpf *hcar1–4*^wt/wt^ or *hcar1–4*^mk213/mk213^ larvae. (**h-i**) Top: Experimental scheme for 5-oxoETE treatment. Larvae were treated with 300 nM 5-oxoETE or ethanol control overnight for 16 h. (**h**) Quantification of neutrophil counts in control and 5-oxoETE treated larvae. (**i**) Quantification of intestinal anti-active caspase-3 immunostaining in control and 5-oxoETE treated larvae. (**j**) Top: Experimental scheme for ZVAD-FMK treatment. Animals were treated for 24 h with 300 μM ZVAD-FMK or vehicle control. Bottom: Quantification of intestinal neutrophil counts. (**k**) Top: Experimental scheme for NS3694 treatment. Animals were treated for 24 h with 10 μM NS3694 or vehicle control. Bottom: Quantification of intestinal neutrophil counts. (**l-m**) Top: Experimental scheme for Liproxstatin-1 (LPX-1) treatment. Larvae were treated with 20 μM LPX-1 or DMSO control for 24 h. (**l**) Quantification of neutrophil counts in control and LPX-1 treated larvae. (**m**) Quantification of intestinal anti-active caspase-3 immunostaining in control and LPX-1 treated larvae. (**n**) Top: Experimental scheme for neutrophil ablation. *Hcar1–4*^mk214/mk214^ larvae expressing the NTR2.0 system in neutrophils [Tg(*lyz*:QF2; 5xQUAS: YFP-NTR2.0)] were treated with 150 μM MTZ or vehicle control for 24 h. Bottom: Quantification of intestinal apoptosis in control or neutrophil depleted larvae. Data are plotted as Violin plot error bars, dashed lines denote third quartiles (top line), medians (middle line), first quartiles (bottom line). Parentheses, total number of larvae per condition. P values, unpaired parametric, two-tailed Welch’s *t*-test was used in d, unpaired, nonparametric, two tailed Mann-Whitney Test was used in f, g and n, Kruskal-Wallis ANOVA followed by Dunn’s *post hoc* test was used in b-c,e,h-k and m. One-way ANOVA followed by Tukey’s *post hoc* test was used in l. Asterisks, highlight significant changes as compared to respective control (p < 0.05). All data are from two independent experiments, except panels j and m, which are each from one independent experiment. The caspase-inhibition phenotype in j was independently reproduced with two additional inhibitors (Fig. 4d and Extended Data Fig. 7k), and the lack of effect of LPX-1 on apoptosis in m was independently reproduced in human Caco-2 cells (Extended Data Fig. 9a,b; N = 3 independent experiments).

**Extended Data Fig. 8 |:**
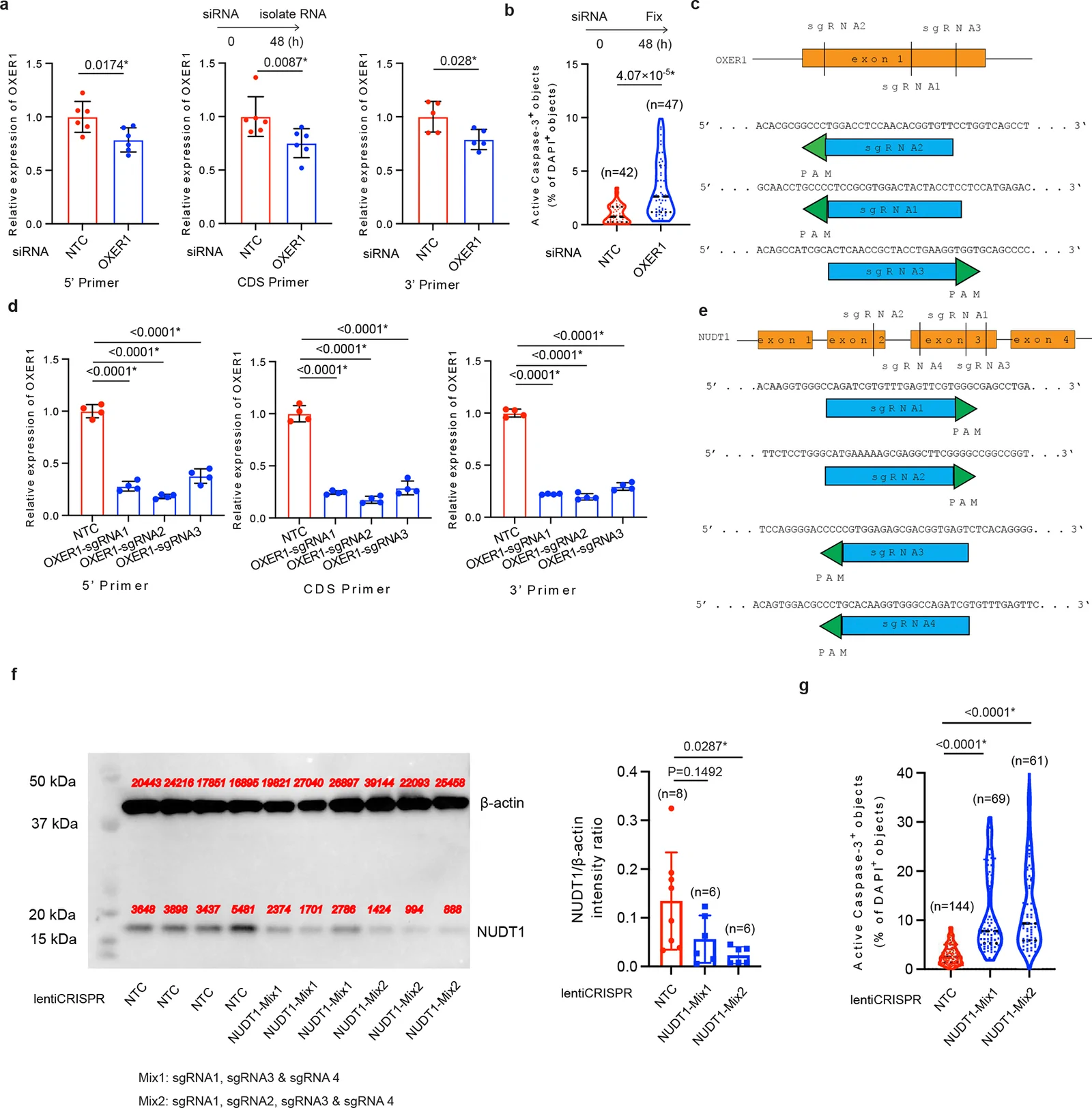
Extended data on oxoeicosanoid signaling in Caco-2 cells, related to Fig. 5. (**a**) Top: Experimental scheme. Caco-2 monolayers were transfected with OXER1 targeting or non-targeting control (NTC) siRNA. Total RNA was isolated 48 h after transfection and used for cDNA synthesis. Quantitative PCR was performed using primers specific for the 5′ UTR (left) the coding sequence (middle) and the 3′ UTR (right) of OXER1. (**b**) Top: Experimental scheme. Caco-2 monolayers were transfected with OXER1 targeting or non-targeting control siRNA. Cells were fixed and used for anti-active caspase-3 immunostaining 48 h after transfection. Bottom: Quantification of apoptosis. (**c**) Schematic diagram showing the design of three different single guide RNAs (sgRNAs) to generate OXER1 CRISPR knockdown cell lines. (**d**) Total RNA was extracted from Caco-2 cells infected with non-targeting control (NTC) or OXER1 targeting guides and used for qPCR as in **a**. (**e**) Schematic diagram showing the design of four different single guide RNAs (sgRNAs) to generate NUDT1 CRISPR knockdown cell lines. (**f**) Western blot of non-targeting control or NUDT1 knockdown Caco-2 cell lines obtained by infecting Caco-2 cells with a mixture of NTC or NUDT1-targeting guides as labelled below the Western blot. Blots were simultaneously probed with anti-β-actin and anti-NUDT1 antibodies. Labels mark the expected molecular weight of β-actin or NUDT1. Quantification of NUDT1 and β-actin band intensities are labelled with red. Right: Graph shows quantification of NUDT1 band intensity normalized to the β-actin band intensity. (**g**) Quantification of apoptosis in two different NUDT1 knockdown cell lines. Data shown are from N = 2 (a, b, d and f) or N = 3 (g) independent experiments. Data are plotted as mean ± SD (a,d,f) or in the case of b and g, as Violin plot error bars, dashed lines denote third quartiles (top line), medians (middle line), first quartiles (bottom line). Parentheses indicate total biological replicates (RNA for a and c, or in the case of d, protein isolated from a single well) or fields of vision (b and g) per condition. P values (middle panel of a and b), unpaired, nonparametric, two tailed Mann-Whitney Test. P values (left and right panels of a), non-paired, parametric, two tailed Welch’s t test. P values (d), One-Way ANOVA followed by Dunnett’s *post hoc* test. P values (f), Brown-Forsythe ANOVA followed by Dunnett’s *post hoc* test. P values (g), Kruskal-Wallis ANOVA followed by Dunn’s *post hoc* test. Asterisks, highlight significant changes as compared to respective control (p < 0.05).

**Extended Data Fig. 9 |:**
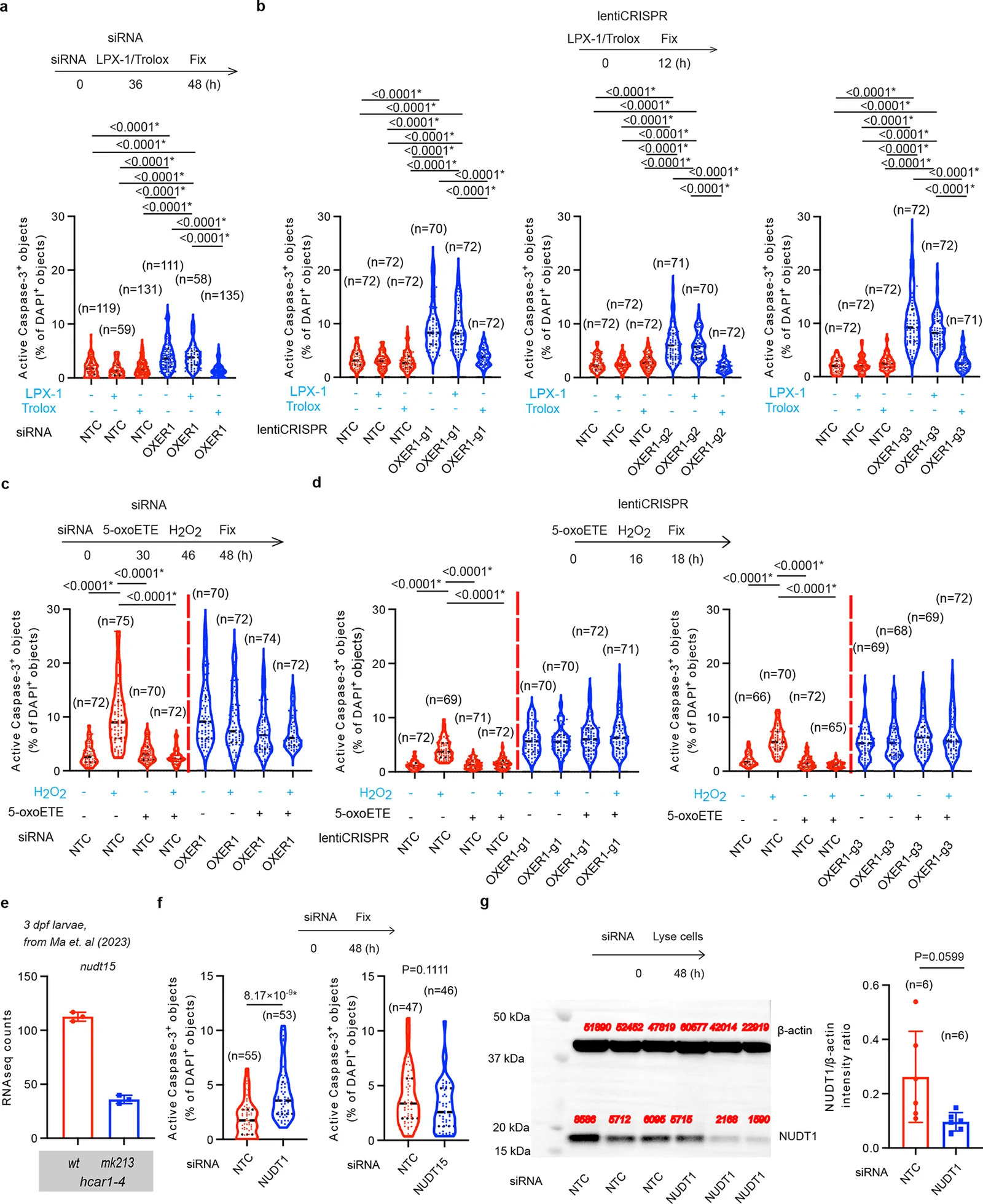
Extended data on oxoeicosanoid signaling in Caco-2 cells, related to Fig. 5. (**a**) Top: Experimental scheme. Caco-2 monolayers were transfected with OXER1 targeting or non-targeting control siRNA. Cells were treated with 20 μM LPX-1, 50 μM Trolox or vehicle control 36 h after transfection (overnight treatment). Cells were fixed and used for anti-active caspase-3 immunostaining 48 h after transfection. Bottom: Quantification of apoptosis. (**b**) Top: Experimental scheme. Non-targeting control (NTC) or OXER1 knockdown Caco-2 cells were treated with 20 μM LPX-1, 50 μM Trolox or vehicle control overnight. Cells were then fixed and used for anti-active caspase-3 immunostaining. Bottom: Quantification of apoptosis in three different OXER1 knockdown cell lines. (**c**) Top: Experimental scheme. Caco-2 monolayers were transfected with OXER1 targeting or non-targeting control siRNA. 36 h after transfection, cells were treated with 1 μM 5-oxoETE or vehicle control overnight. Cells were then challenged with 1 mM H_2_O_2_ or vehicle control for 2 h. Cells were fixed and used for anti-active caspase-3 immunostaining 48 h after transfection. Bottom: Quantification of apoptosis. All OXER1 silenced groups were significantly different from the siCtl EtOH, Vehicle; 5-oxoETE, Vehicle; 5-oxoETE, H_2_O_2_ groups. The siCtl, EtOH, H_2_O_2_ group was not significantly different from any of the OXER1 silenced groups. (**d**) Non-targeting control (NTC) or OXER1 knockdown Caco-2 cells were treated with 1 μM 5-oxoETE or vehicle control overnight. Cells were then challenged with 1 mM H_2_O_2_ or vehicle control for 2 h. Cells were fixed and used for anti-active caspase-3 immunostaining. Bottom: Quantification of apoptosis in two different OXER1 knockdown cell lines. (**e**) mRNASeq counts for *nudt15* from 3dpf *hcar1–4*^wt/wt^ and *hcar1–4*^mk213/mk213^ larvae. Replotted from^11^, source data deposited in GEO under number GSE201604. (**f**) Top: Experimental scheme. Caco-2 monolayers were transfected with either non-targeting control siRNA or siRNA targeting NUDT1 (left) or NUDT15 (right). Cells were fixed and used for anti-active caspase-3 immunostaining 48 h after transfection. Bottom: quantification of apoptosis. (**g**) Top: Experimental scheme. Caco-2 monolayers were transfected with either non-targeting control siRNA or siRNA targeting NUDT1. Cells were lysed for Western Blot analysis 48 h after transfection. Left: Western blot of Caco-2 cells transfected with either control siRNA or NUDT1 siRNA. Blots were simultaneously probed with anti-β-actin and anti-NUDT1 antibodies. Labels mark the expected molecular weight of β-actin or NUDT1. Quantification of NUDT1 and β-actin band intensities are labelled with red. Right: Graph shows quantification of NUDT1 band intensity normalized to the β-actin band intensity. Data shown are from N = 2 (f-g), or N = 3 (a-d) independent experiments. Data are plotted as Violin plot error bars, dashed lines denote third quartiles (top line), medians (middle line), first quartiles (bottom line) or in g, as mean ± SD. Parentheses, total number of fields of vision per condition or independent wells in the case of g. P values, unpaired, nonparametric, two tailed Mann-Whitney Test was used in f, unpaired, parametric, two tailed Welch’s t-test was used in g and Kruskal-Wallis ANOVA followed by Dunn’s *post hoc* test was used in a-d. Asterisks, highlight significant changes as compared to respective control (p < 0.05).

**Extended Data Fig. 10 |:**
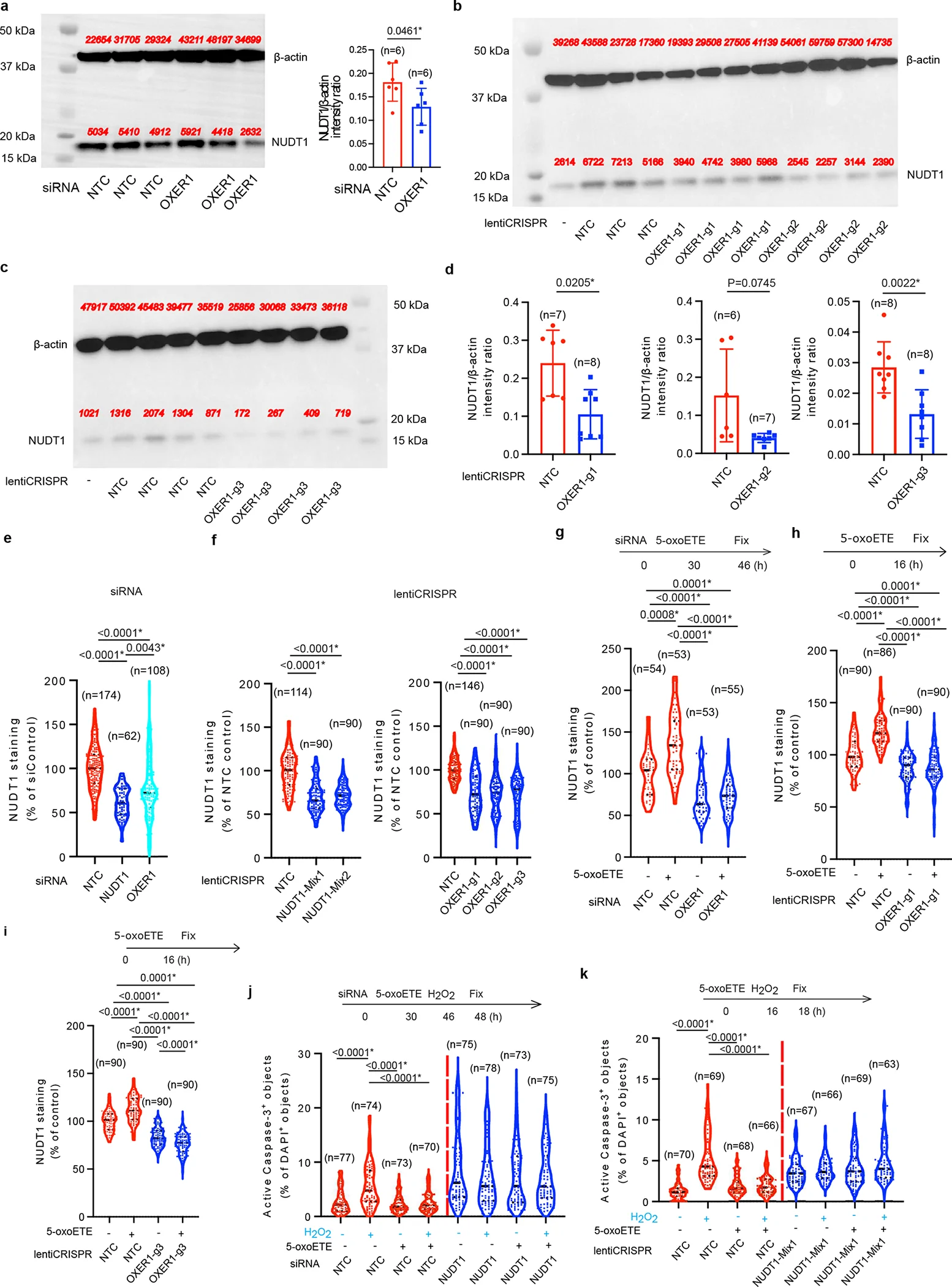
Extended data on oxoeicosanoid signaling in Caco-2 cells, related to Fig. 5. (**a**) Western blot of Caco-2 cells transfected with either control siRNA or OXER1 siRNA. Blots were simultaneously probed with anti-β-actin and anti-NUDT1 antibodies. Quantification of NUDT1 and β-actin band intensities are labelled with red. Graph shows quantification of NUDT1 band intensity normalized to the β-actin band intensity. (**b-c**) Western blot of non-targeting control or OXER1 knockdown Caco-2 cells. Blots were simultaneously probed with anti-β-actin and anti-NUDT1 antibodies. Labels mark the expected molecular weight of β-actin or NUDT1. Quantification of NUDT1 and β-actin band intensities are labelled with red. Note the increased NUDT1 band intensity in cells infected with NTC viruses compared to non-infected control cells. (**d**) Graphs show quantification of NUDT1 band intensity normalized to the β-actin band intensity. (**e**) Quantification of NUDT1 immunostaining in Caco-2 monolayers transfected with either non-targeting control siRNA, siRNA targeting NUDT1 or siRNA targeting OXER1. Cells were fixed and used for anti-NUDT1 immunostaining 48 h after transfection. (**f**) Quantification of NUDT1 immunostaining in NUDT1 (left) or OXER1 knockdown (right) Caco-2 cells. (**g**) Top: Experimental scheme. Caco-2 cells were transfected with non-targeting control siRNA or siRNA targeting OXER1. Cells were treated with 1 μM 5-oxoETE or vehicle control overnight. Cells were then fixed and used for anti-NUDT1 immunostaining 48 h after transfection. Bottom: Quantification of anti-NUDT1 immunofluorescence. (**h-i**) Top: Experimental scheme. Non-targeting control or OXER1 knockdown Caco-2 cells were treated with 1 μM 5-oxoETE or vehicle control overnight. Cells were then fixed and used for anti-NUDT1 immunostaining. Bottom: Quantification of anti-NUDT1 immunofluorescence. (**j**) Top: Experimental scheme. Caco-2 monolayers were transfected with NUDT1 targeting or non-targeting control siRNA. 36 h after transfection, cells were treated with 1 μM 5-oxoETE or vehicle control overnight. Cells were then challenged with 1 mM H_2_O_2_ or vehicle control for 2 h. Cells were fixed and used for anti-active caspase-3 immunostaining 48 h after transfection. Bottom: Quantification of apoptosis. All NUDT1 silenced groups were significantly different from the siCtl EtOH, Vehicle; 5-oxoETE, Vehicle; 5-oxoETE, H_2_O_2_ groups. The siCtl, EtOH, H_2_O_2_ group was not significantly different from any of the NUDT1 silenced groups. (**k**) Top: Experimental scheme. Non-targeting control (NTC) or NUDT1 knockdown Caco-2 cells were treated with 1 μM 5-oxoETE or vehicle control overnight. Cells were then challenged with 1 mM H_2_O_2_ or vehicle control for 2 h. Cells were fixed and used for anti-active caspase-3 immunostaining. Bottom: Quantification of apoptosis in two different NUDT1 knockdown cell lines. All NUDT1 knockdown groups were significantly different from the NTC, Ethanol, Vehicle; NTC, 5-oxoETE, Vehicle and NTC, 5-oxoETE, H_2_O_2_ groups. Data are plotted as Violin plot error bars, dashed lines denote third quartiles (top line), medians (middle line), first quartiles (bottom line) or in the case of a and d, as mean ± SD. Parentheses, total number of cell lysates (a and d) or fields of vision (e-k) per condition. Data shown are from N = 2 (a and d), or N = 3 (e-k) independent experiments. P values, unpaired parametric, two-tailed Welch’s *t*-test was used in a and d (middle and right panels), unpaired, nonparametric, two tailed Mann-Whitney Test was used in d (left panel) and Kruskal-Wallis ANOVA followed by Dunn’s *post hoc* test was used in e,f,g,h,j and k and Brown-Forsythe ANOVA followed by Dunnett’s *post hoc* test was used in i. Asterisks, highlight significant changes as compared to respective control (p < 0.05).

**Extended Data Fig. 11 |:**
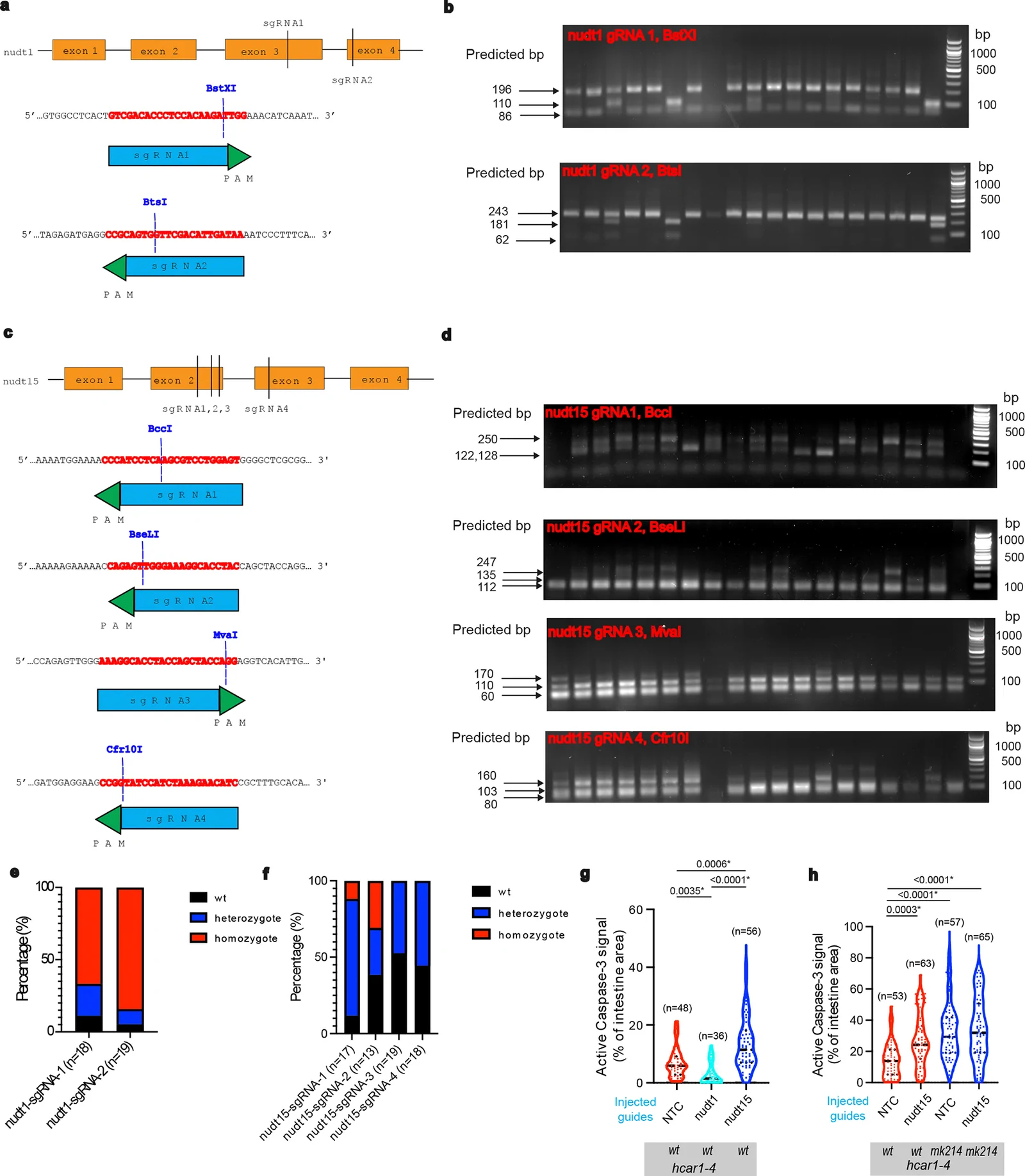
F0 CRISPR approach for *nudt1* and *nudt15*, related to Fig. 5. (**a**,**c**) Schematic representation of zebrafish *nudt1* and *nudt15*. 2 guide RNAs (red text) were designed to disrupt exons 3 and 4 (*nudt1)* and 4 guide RNAs were designed to disrupt exons 2 and 3 (*nudt15*) and to disrupt restriction enzyme recognition sites (cleavage site marked with a blue dash) in these genes. (**b**,**d**) *nudt1* and *nudt15* F0 CRISPR larvae (18 or 19 per mutant) were genotyped by PCR amplification, restriction analysis, and agarose gel electrophoresis. For *nudt1* guide RNA-1, the digest of *wt* samples yields two lower bands of 110 and 86 bp, digested heterozygotes have an additional higher band of 196 bp and homozygotes are uncleaved and result in only the 196 bp band. For *nudt1* guide RNA-2, the digest of *wt* samples results in two bands of 181 and 62 bp, heterozygotes have an additional higher band of 243 bp, while PCR products from homozygotes are uncleaved and only have the 243 bp band. For *nudt15* guide RNA-1, digested PCR products from *wt* samples yield two bands of 122 and 128 bp, heterozygotes have an additional 250 bp band, while in the case of the homozygotes, only the 250 bp band is visible. For *nudt15* guide RNA-2, digested PCR products from *wt* samples yield two bands of 112 and 135 bp, heterozygotes have an additional 247 bp band, while in the case of the homozygotes, only the 247 bp band is visible. For *nudt15* guide RNA-3, digested PCR products from *wt* samples yield two bands of 110 and 60 bp, heterozygotes have an additional 170 bp band, while in the case of the homozygotes, only the 170 bp band is visible (all digests contain additional 42 and 34 bp fragments that can’t be visualized properly). For *nudt15* guide RNA-4, digested PCR products from *wt* samples yield two bands of 103 and 80 bp (with an additional 57 bp fragment that can’t be visualized properly), heterozygotes have an additional 160 bp band, while in the case of the homozygotes, only the 160 bp and 80 bp band is visible. (**e**,**f**) Plots showing the fraction of F0 CRISPR homozygotes, heterozygotes, or *wt* animals among the 18 or 19 tested larvae in b and d. (**g**) Quantification of intestinal apoptosis of *hcar1–4*^wt/wt^ larvae injected with Cas9 and NTC, *nudt1* or *nudt15* guides. Larvae were fixed at 6dpf and used for anti-active caspase-3 immunostaining. (**h**) Quantification of intestinal apoptosis counts of *hcar1–4*^wt/wt^ and *hcar1–4*^mk214/mk214^ larvae injected with Cas9 and NTC or *nudt15* guides. Data are plotted as Violin plot error bars, dashed lines denote third quartiles (top line), medians (middle line), first quartiles (bottom line) or in the case of C, as mean ± SD. Parentheses, total number of larvae per condition. P values, Kruskal-Wallis ANOVA followed by Dunn’s *post hoc* test was used in g and h. Asterisks, highlight significant changes as compared to respective control (p < 0.05). All data shown are from two independent experiments.

## Supplementary Material

Supplementary_Table_1

SI_file

Supplementary_Video1

Supplementary_Video2

Supplementary_Video3

Supplementary_Video4

**Supplementary information** The online version contains supplementary material available at https://doi.org/10.1038/s41586-026-11121-2.

## Figures and Tables

**Fig. 1 |:**
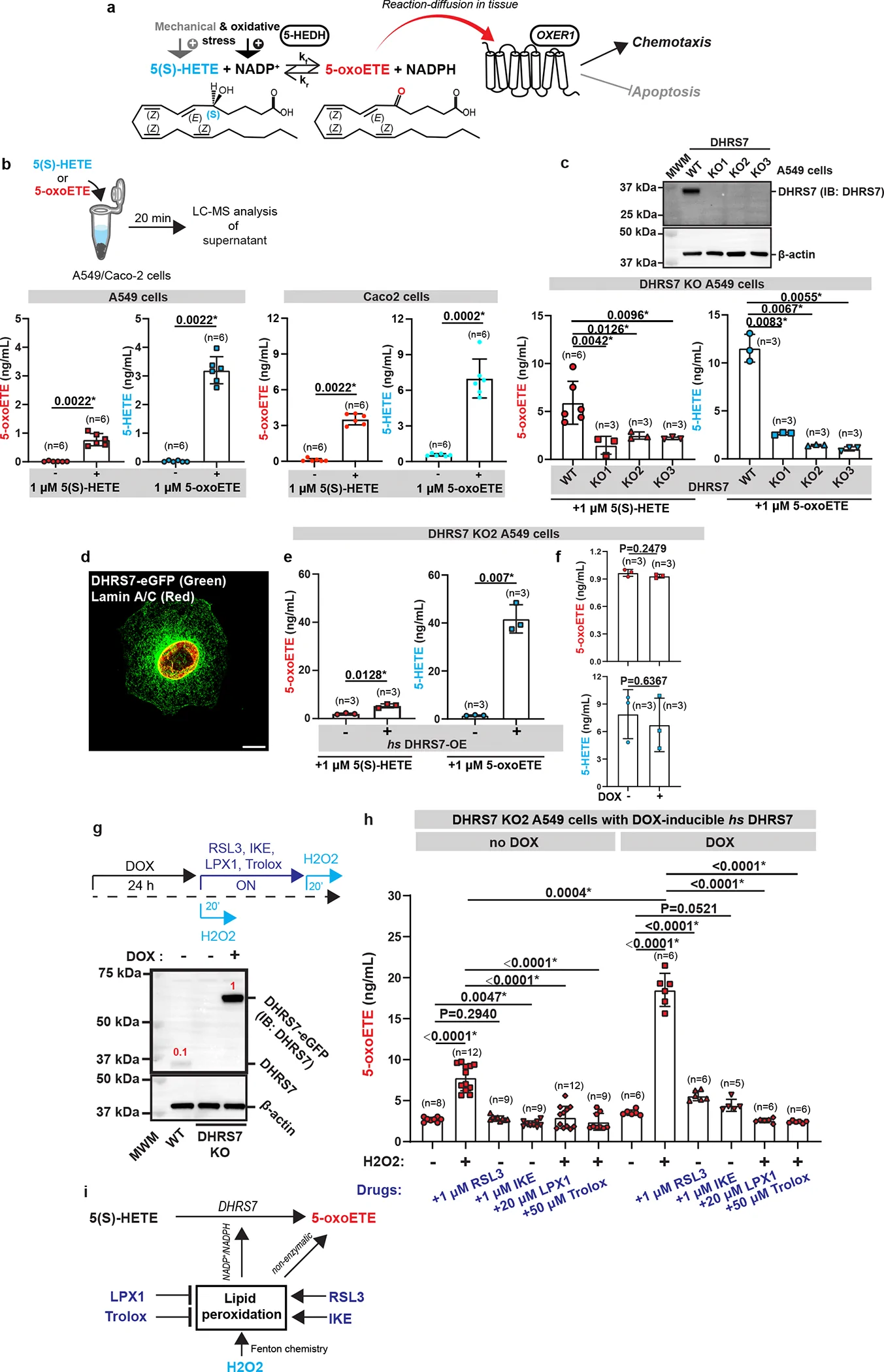
Identification of DHRS7 as 5-HEDH and its role in lipid peroxidation-induced 5-oxoETE production. **a**, Scheme of the bidirectional 5-HEDH reaction and physiological functions of 5-oxoETE. Physical and oxidative stress provide 5(*S*)-HETE and NADP^+^, respectively, promoting 5-oxoETE production and NADPH generation. 5-oxoETE signals through OXER1 to mediate chemotactic and anti-apoptotic effects. k_f_, forward rate constant; k_r_, reverse rate constant. **b**, Top, LC–MS workflow for A549 or Caco-2 cells. Bottom, LC–MS measurements after incubation with 1 μM 5(*S*)-HETE or 5-oxoETE. **c**, Top, western blot of three A549 DHRS7-knockout (KO) lines (KO1–KO3). Bottom, LC–MS measurements following treatment with 1 μM 5(*S*)-HETE or 1 μM 5-oxoETE. IB, immunoblot. **d**, Representative subcellular localization of *Hs*DHRS7–eGFP in A549 cells. Scale bar, 10 μm. *n* = 32 fields of view (FOVs) from 4 wells across 2 independent experiments. This dataset also contributed to Fig. 2i,j and Extended Data Figs. 2e and 4h. **e**, LC–MS of DHRS7-KO2 cells with or without *Hs*DHRS7 overexpression (OE) after treatment with 1 μM 5(*S*)-HETE or 1 μM 5-oxoETE. **f**, 5-oxoETE or 5-HETE production in DHRS7-KO2 cells with or without 1 μg ml^−1^ Dox (without DHRS7 induction). **g**, Top, experimental scheme. Bottom, western blot of endogenous DHRS7 and *Hs*DHRS7–eGFP in wild-type (WT) and DHRS7-KO2 cells after induction with 0.2 μg ml^−1^ Dox. β-Actin, loading control; red numbers represent β-actin-normalized band intensities. **h**, LC–MS measurement of 5-oxoETE under indicated conditions. **i**, Scheme of differential effects of H_2_O_2_, LPX1, Trolox, RSL3 and IKE on 5-oxoETE production. H_2_O_2_ promotes oxidative stress by increasing the NADP^+^/NADPH ratio and may additionally stimulate non-enzymatic lipid oxidation; LPX1 and Trolox counteract ROS, whereas RSL3 and IKE promote phospholipid peroxidation by inhibiting GPX4 and SLC7A11 pathways, respectively. Two-tailed Mann–Whitney tests (**b**, A549 and Caco-2, left); two-tailed Welch’s *t*-tests (**b**, Caco-2, right; and **c**,**e**,**f**); Brown–Forsythe and Welch ANOVA tests (**h**). Bars indicate mean ± s.d.; parentheses indicate the number of biological replicates from independent cultures in **b**,**c**,**e**,**f**,**h**. Pictogram in **b** from NIH BioArt; https://bioart.niaid.nih.gov/bioart/143 (2024).

**Fig. 2 |:**
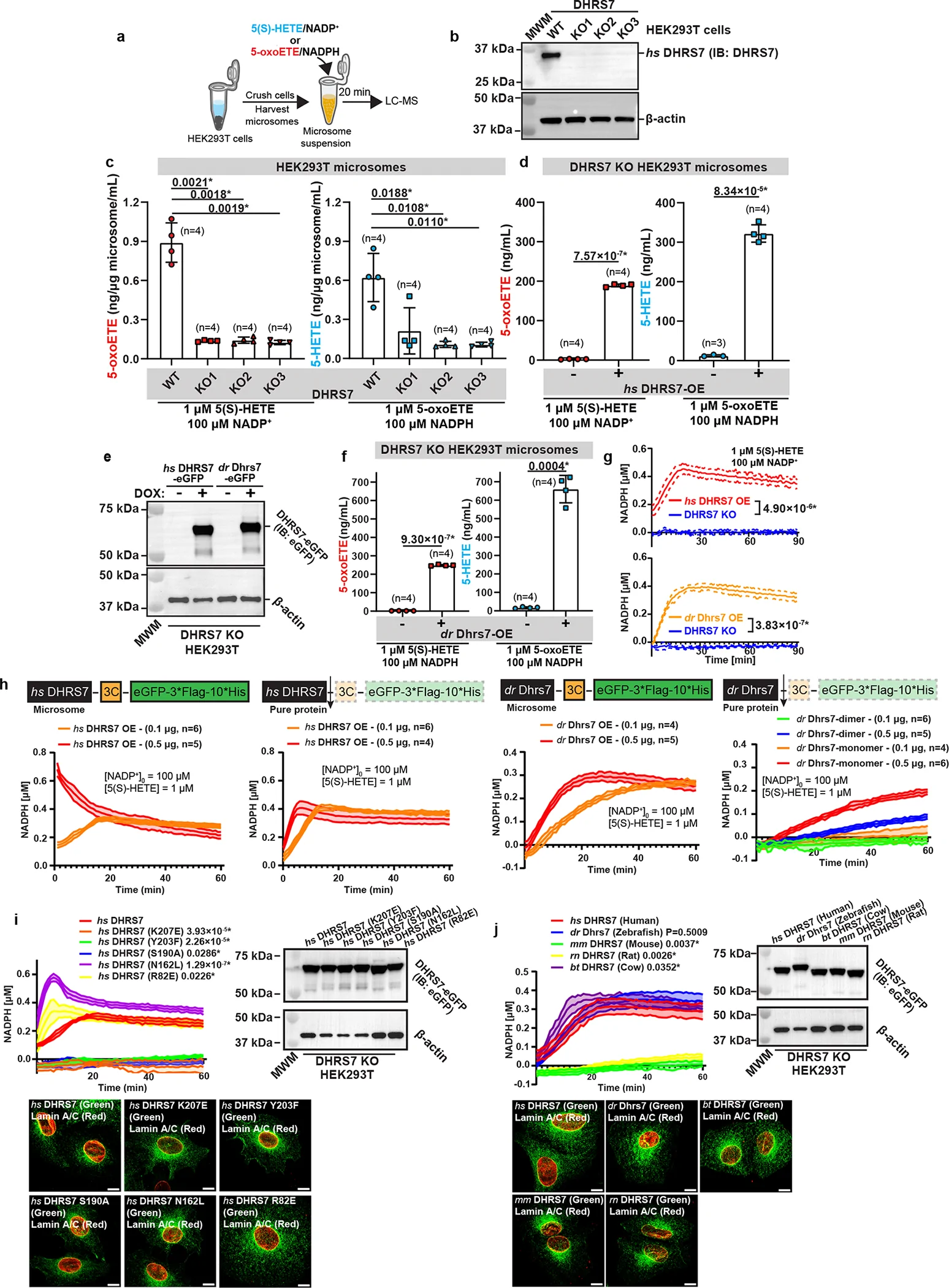
5-HEDH activity of DHRS7 is phylogenetically conserved. **a**, Scheme for LC–MS measurement of microsomal DHRS7 activity. **b**, Western blot of three HEK293T DHRS7-KO lines. **c**, LC–MS assays with microsomes from HEK293T wild-type and *Hs*DHRS7-KO cells at the indicated substrate concentrations. **d**, LC–MS assays using microsomes from *Hs*DHRS7-KO1 (20 μg) and *Hs*DHRS7-OE HEK293T cells (20 μg, 4.39 pmol *Hs*DHRS7–eGFP). **e**, Western blot confirming equal amounts of *Hs*DHRS7–eGFP and *Dr*Dhrs7–eGFP in microsomes used in **d**–**g**. **f**, As in **d**, using 16 μg microsomes containing 4.39 pmol *Dr*Dhrs7–eGFP. MWM, molecular weight marker. **g**, NADPH production by *Hs*DHRS7-KO1 versus *Hs*DHRS7-OE (top; 10 μg) or *Dr*Dhrs7-OE (bottom; 8 μg) microsomes. *P* values reflect data at 20 min; *n* = 5 biological replicates from independent wells. **h**, NADPH production using microsomes or purified *Hs*DHRS7 or *Dr*Dhrs7 (0.1 or 0.5 μg) from HEK293S GnTI^−^ cells. *n* represents the number of biological replicates from independent wells. **i**, NADPH production by *Hs*DHRS7 point mutants (top left), corresponding western blot (top right) and representative localization in A549 cells (bottom; DHRS7-eGFP, green; lamin A/C, red). *n* = 4 biological replicates from different wells. K207E, 11 FOVs; Y203F, 10 FOVs; S190A, 12 FOVs; N162L, 15 FOVs; R82E, 16 FOVs; from two different wells. *P* values reflect data at 5 min. **j**, NADPH production by human, zebrafish, mouse (*Mus musculus*, *Mm*DHRS7), rat (*Rattus norvegicus*, *Rn*DHRS7) and bovine (*Bos taurus*, *Bt*DHRS7) DHRS7 orthologues (top left), corresponding western blot (top right) and representative localization in A549 cells (bottom; DHRS7/Dhrs7-eGFP, green; lamin A/C, red). Biological replicates were from independent wells; *Hs*DHRS7, *n* = 4; *Dr*Dhrs7, *n* = 4; *Mm*DHRS7, *n* = 5; *Rn*DHRS7, *n* = 5; *Bt*DHRS7, *n* = 4); *Dr*Dhrs7, 17 FOVs; *Mm*DHRS7, 15 FOVs; *Rn*DHRS7, 15 FOVs; *Bt*DHRS7, 15 FOVs; from two different wells. **i**,**j**, Scale bars, 10 μm. Two-tailed Welch’s *t*-tests were used in **c**,**d**,**f**,**g**,**i**,**j**, except for S190A in **i** (two-tailed Mann–Whitney test). Bars or lines show mean ± s.d. All microsome and LC–MS experiments were performed once with the indicated independent biological replicates from separate transfections. Pictogram in **a** from NIH BioArt; https://bioart.niaid.nih.gov/bioart/143 (2024).

**Fig. 3 |:**
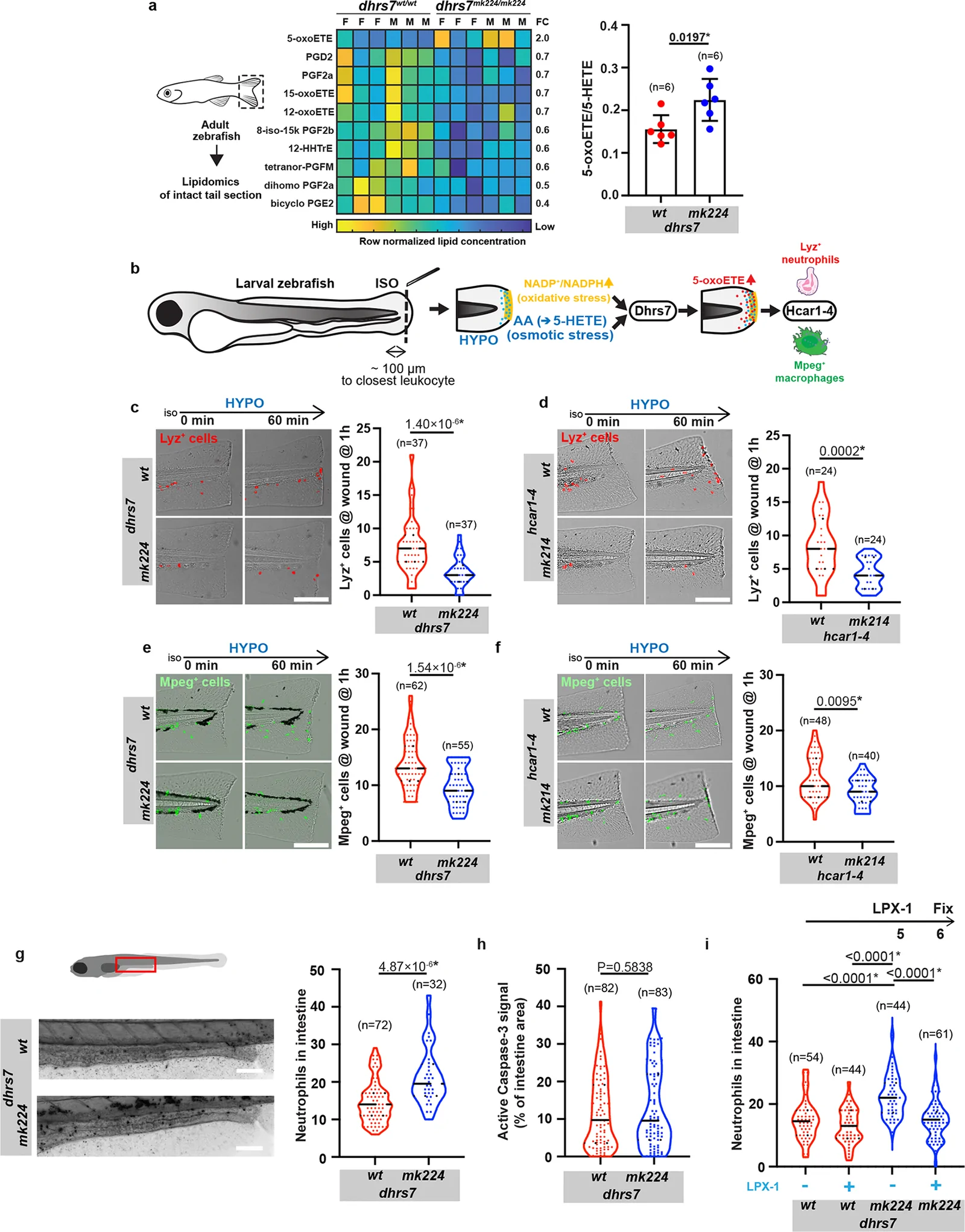
Dhrs7–5-HEDH mediates rapid wound detection in live zebrafish. **a**, Left, scheme of adult zebrafish tail fin lipidomics. Middle, heat map of significantly altered lipids in *dhrs7*^*wt*/*wt*^ versus *dhrs7*^*mk224*/*mk224*^ fish from the University of California, San Diego LIPIDMAPS eicosanoid panel (Supplementary Table 1). Concentrations are normalized by row. Right, 5-oxoETE/5-HETE ratios from the same experiment. Six biological replicates, each pooling around 0.1 g tail fin tissue. Two-tailed Welch’s *t*-test or Mann–Whitney test was used, according to Shapiro–Wilk normality testing (irrespective of sex); 5-oxoETE/5-HETE ratios used two-tailed Welch’s *t-*test. 12-HHTrE, 12-hydroxyheptadecatrienoic acid; F, female; FC, fold change; M, male; PGD_2_, prostaglandin D_2_; PGF_2α_, prostaglandin F_2α_; PGF_2β_, prostaglandin F_2β_; PGFM, 13,14-dihydro-15-keto-prostaglandin F_2α_. **b**, Experimental workflow and hypothesis. Isotonic bathing solution (iso) inhibits cPla_2_-dependent arachidonic acid (AA) release and 5(*S*)-HETE generation^10,36^; hypotonic, ‘freshwater-like’ bathing solution (hypo) produces osmotic stress to induces these processes^10,36^. Duox-mediated H_2_O_2_ generation (oxidative stress) at the wound margin consumes NADPH^2,7^. Dhrs7 may integrate 5(*S*)-HETE and NADP^+^ changes to promote neutrophils (*lyz*^+^) and macrophages (*mpeg*^+^) chemotaxis via the OXER1 orthologue Hcar1–4. **c**,**d**, Live imaging of neutrophil recruitment to wounds in wild-type siblings and *dhrs7*^*mk224*/*mk224*^ (**c**) or *hcar1–4*^*mk214*/*mk214*^ (**d**) mutant larvae following shift from Iso to Hypo conditions (Tg(*lyz*:pm2-mk2) background). Left, representative images; right, quantification. Red, mKate2-expressing neutrophils. **e**,**f**, Live imaging of macrophage recruitment to wounds in wild-type siblings and *dhrs7*^*mk224*/*mk224*^ (**e**) or *hcar1–4*^*mk214*/*mk214*^ (**f**) mutant larvae (Tg(*mpeg1*:eGFP) background) following shift from Iso to Hypo conditions. Left, representative images; right, quantification. Green, eGFP-expressing macrophages. **c**–**f**, Scale bars, 200 μm. **g**, Representative Sudan Black staining (left) and intestinal neutrophil counts (right) in wild-type and *dhrs7*^*mk224/mk224*^ larvae. Scale bars, 100 μm. **h**, Quantification of intestinal active caspase-3 immunostaining in wild-type and *dhrs7*^*mk224/mk224*^ larvae. **i**, Top, LPX1 treatment scheme. Bottom, intestinal neutrophil counts after 24 h of LPX1 or DMSO treatment in wild-type and *dhrs7*^*mk224/mk224*^ larvae. Violin plots show the data distribution; dashed lines indicate the third quartiles (top line), medians (middle line) and first quartiles (bottom line). *n* represents total number of larvae. Two-tailed Mann–Whitney tests (**c**–**h**); Kruskal–Wallis ANOVA followed by Dunn’s post hoc test (**i**). Two (**c**,**d**,**f**–**i**) or three (**e**) independent experiments. Bars denote mean ± s.d. Pictograms in **b** from NIH BioArt; https://bioart.niaid.nih.gov/bioart/378 (2024); https://bioart.niaid.nih.gov/bioart/307 (2024). Graphic in **g** from ref. 51, CC BY 4.0.

**Fig. 4 |:**
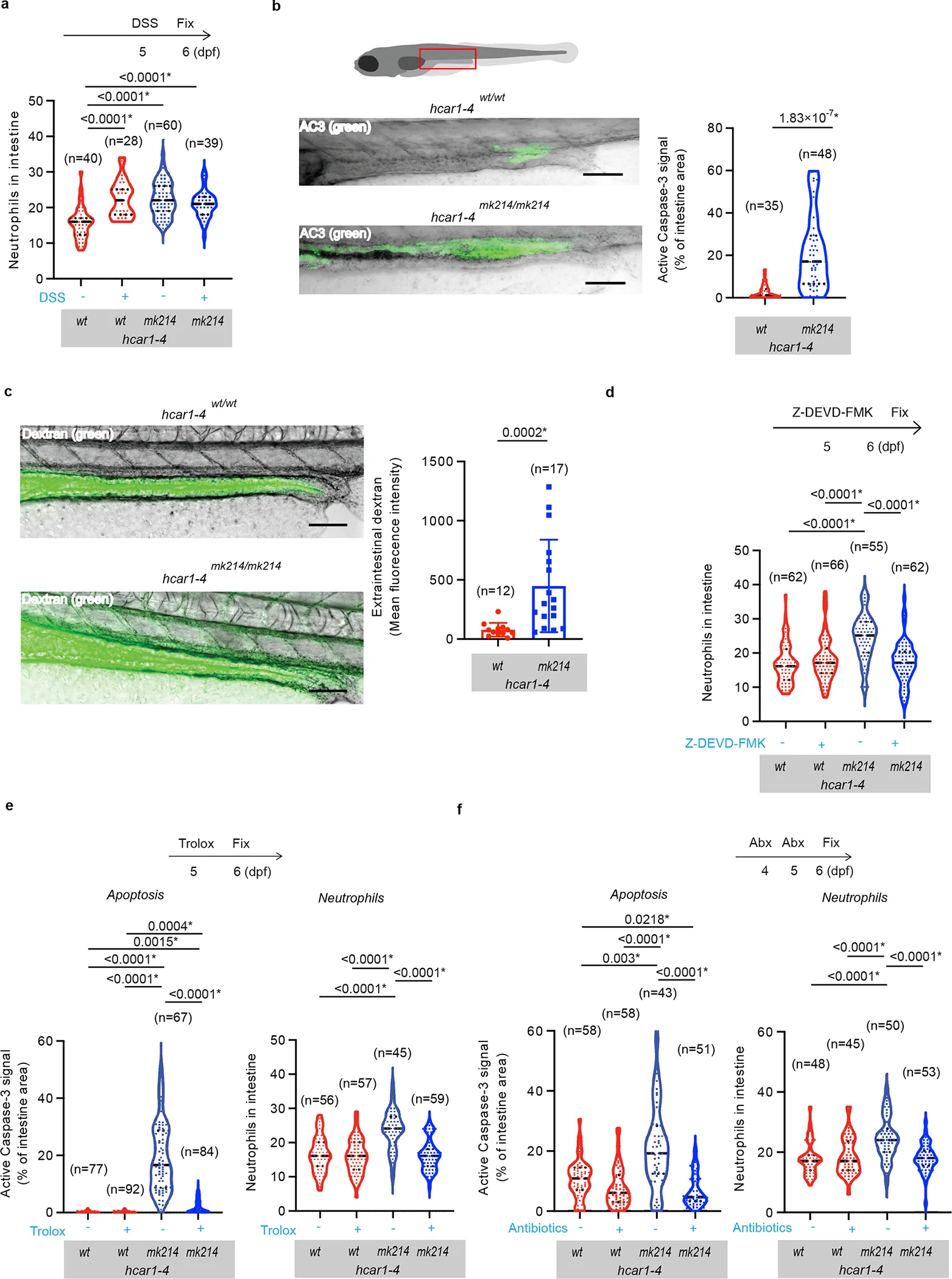
Spontaneous intestinal inflammation in *hcar1–4*-deficient zebrafish. **a**, Top, experimental scheme for the DSS colitis model. Bottom, quantification of intestinal neutrophil counts. dpf, days post-fertilization. **b**, Left, representative images of active caspase-3 (AC3) immunostaining of *hcar1–4*^wt/wt^ and *hcar1–4*^mk214/mk214^ larvae. Scale bars, 100 μm. Right, quantification of intestinal active caspase-3 immunostaining. **c**, Left, representative images of fluorescein isothiocyanate (FITC)–dextran-gavaged larvae 60 min after gavage. Scale bars, 100 μm. Right, quantification of extraintestinal dextran fluorescence intensity. **d**, Top, experimental scheme for Z-DEVD-FMK treatment. Fish were treated with 150 μM Z-DEVD-FMK or DMSO control. Bottom, quantification of intestinal neutrophil counts. **e**, Top, experimental scheme for Trolox treatment. Larvae were treated with 50 μM Trolox or DMSO control. Bottom, quantification of apoptosis (left) and neutrophil counts (right) in control and Trolox-treated larvae. **f**, Top, scheme for antibiotics (Abx) treatment. Larvae were treated with ampicillin and kanamycin for 48 h. Embryo water was switched to fresh water with new antibiotics after 24 h of treatment. Bottom, quantification of apoptosis (left) and neutrophil counts (right) in control and antibiotics-treated larvae. Data are plotted as violin plots, dashed lines denote third quartiles (top line), medians (middle line) and first quartiles (bottom line) (**a**,**b**,**d**–**f**), or mean ± s.d. (**c**). *n* represents total number of larvae per condition. Unpaired, nonparametric, two-tailed Mann–Whitney test (**b**,**c**); one-way ANOVA followed by Tukey’s post hoc test (**a**); or Kruskal–Wallis ANOVA followed by Dunn’s post hoc test (**d**–**f**). Data are from two independent experiments (separate clutches of zebrafish larvae; results were similar between different clutches), except in **b**, which represents one independent experiment; the difference in apoptosis between *hcar1–4*^*wt/wt*^ and *hcar1–4*^*mk214/mk214*^ intestines was independently reproduced in **e**,**f**, each of which is from two independent experiments. Graphic in **b** from ref. 51, CC BY 4.0.

**Fig. 5 |:**
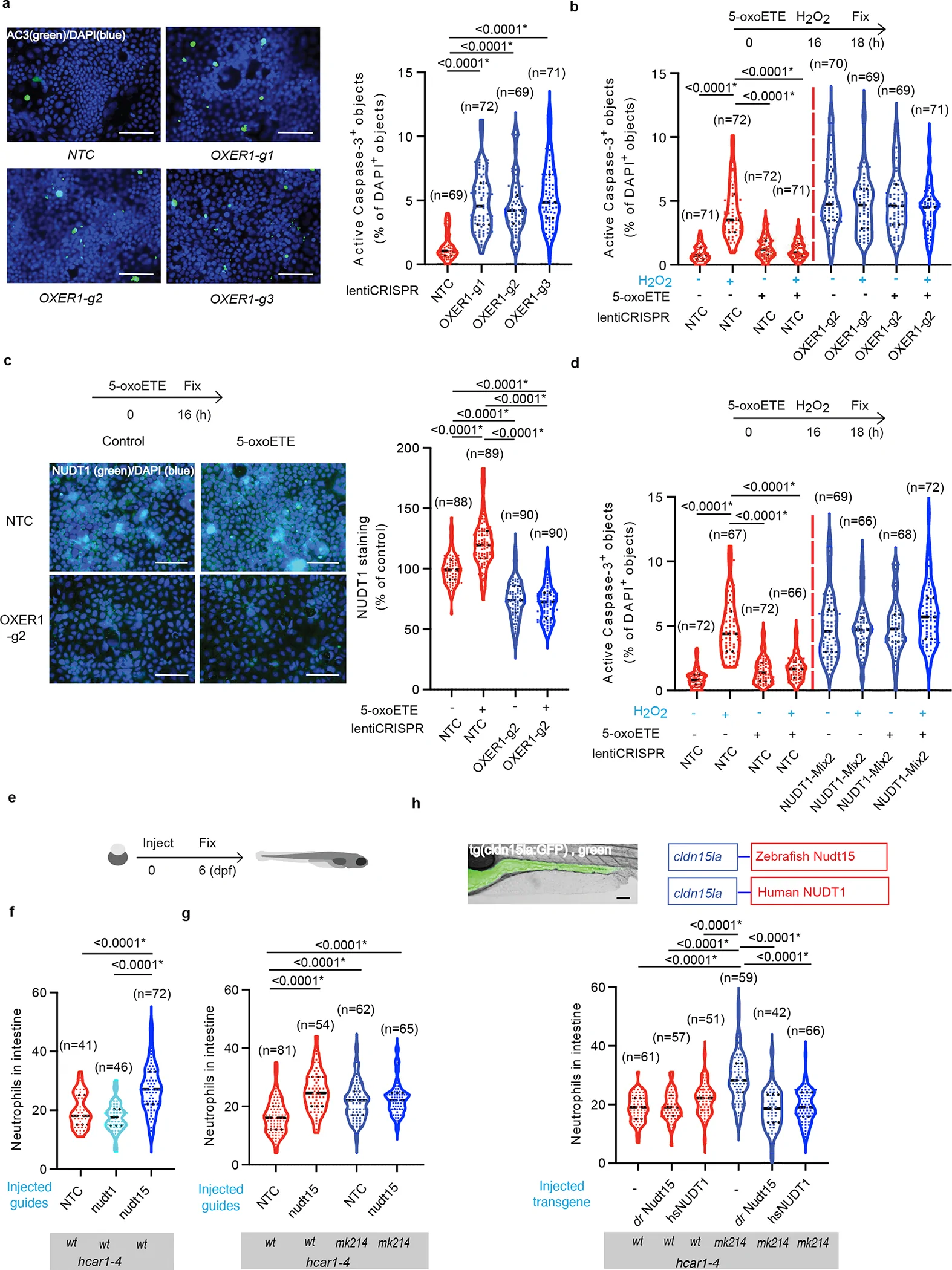
The protective effects of 5-oxoETE are mediated by NUDIX hydrolases in human cells and zebrafish. **a**, Anti-active caspase-3 immunostaining of NTC-treated or OXER1-knockdown Caco-2 cells. Left, representative microscopic images of active caspase-3 immunostaining. Scale bars, 100 μm. Right, quantification of apoptosis. **b**, Top, NTC-treated or OXER1-knockdown cells were treated overnight with 1 μM 5-oxoETE or vehicle, challenged with 1 mM H_2_O_2_ or vehicle for 2 h and used for anti-active caspase-3 immunostaining. Bottom, quantification of apoptosis. **c**, NTC-treated or OXER1-knockdown cells were treated overnight with 1 μM 5-oxoETE or vehicle, fixed and used for anti-NUDT1 immunostaining. Bottom left, representative microscopic images of anti-NUDT1-immunostained cells. Scale bars, 100 μm; Bottom right, quantification of anti-NUDT1 immunofluorescence. **d**, NTC-treated or NUDT1-knockdown cells were treated overnight with 1 μM 5-oxoETE or vehicle, challenged with 1 mM H_2_O_2_ or vehicle for 2 h, and used for anti-active caspase-3 immunostaining. **e**–**h**, Schematic (**e**) showing injection of zebrafish embryos at the 1-cell stage with Cas9 and guide RNAs (NTC, *nudt1* or *nudt15*) (**f**,**g**) or Tol2 transposase mRNA and plasmids driving intestinal epithelial expression of zebrafish Nudt15 or human NUDT1 (**h**). **f**,**g**, Intestinal neutrophil counts in *hcar1–4*^*wt/wt*^ or *hcar1–4*^mk214/mk214^ larvae injected with Cas9 and NTC, *nudt1* or *nudt15* guide RNAs. **h**, Top left, representative intestinal epithelial GFP expression in Tg(*cldn15la*:QF2×QUAS:eGFP) fish (100 μm; representative of 15 larvae from 3 clutches). The *cldn15la:*QF2 transgenic line has been used to drive the expression of other reporters resulting in a similar expression pattern. Top right, transgenic construct scheme. Bottom, intestinal neutrophil counts in *hcar1–4*^*wt/wt*^ and *hcar1–4*^*mk214/mk214*^ larvae expressing zebrafish Nudt15 or human NUDT1. Data are plotted as violin plots, dashed lines denote third quartiles (top line), medians (middle line) and first quartiles (bottom line). *n* represents total number of FOVs (**a**–**d**) or larvae per condition (**e**–**h**). Kruskal–Wallis ANOVA followed by Dunn’s post hoc test (**a**–**g**) and Brown–Forsythe ANOVA followed by Dunnett’s post hoc test (**h**). Data are from two (**e**–**h**) or three (**a**–**d**) independent experiments (separate clutches of zebrafish larvae or batches of cells. Results were similar between independent experiments). Graphic in **e** from ref. 51, CC BY 4.0.

## Data Availability

Data supporting the findings of this study are available within the Article, Supplementary Information and Source Data files. Additional datasets underlying the analyses, including the lipidomics dataset, are publicly available in the accompanying GitHub repository (https://github.com/zazadovv/Redox-DHRS7_subcellular) and its permanently archived Zenodo record (https://doi.org/10.5281/zenodo.22147049 (ref. 101)). Oligonucleotide sequences used in this paper are detailed in Supplementary Table 2. Plasmids used in this study are detailed in Supplementary Table 3. RNA-sequencing data that are re-analysed in Extended Data Fig. 9e were previously published^11^ and are available in GEO under accession GSE201604.

## Code availability

Analysis scripts and image- and data-processing pipelines used in this study are publicly available in the GitHub repository (https://github.com/zazadovv/Redox-DHRS7_subcellular). The repository includes Python code for quantitative microscopy and co-localization analysis, Fiji macros for image preprocessing, MATLAB code for lipidomics statistical analysis and Michaelis–Menten kinetic analysis, and Python-based tools for comparative DHRS7 protein sequence and structural analyses. The version of the code associated with this publication has been permanently archived in Zenodo (https://zenodo.org/records/22147049 (ref. 101)).
