# Supplementary material for: 5-oxoETE links redox control of epithelial damage detection and resilience
Source: Nature. Author manuscript; Available in PMC 2026 Nov 13. (PMC13766681; doi:10.1038/s41586-026-11121-2)
Supplement: Supplementary file 1 [file NIHMS2218767-supplement-SI_file.pdf]

nature portfolio

https://doi.org/10.1038/s41586-026-11121-2

Supplementary information

5-oxoETE links redox control of epithelial damage detection and resilience

In the format provided by the authors and unedited

Table S2. Oligonucleotides used in this paper.

REAGENT or RESOURCE	SOURCE	IDENTIFIER
Oligonucleotides	This paper	N/A
DHRS7-sgRNA-1: CCCCTTGACCTGACCGACAC TGG	This paper	N/A
DHRS7-sgRNA-2: TATCCATGCACAGAGAACGC TGG	This paper	N/A
dhrs7-sgRNA-1: GGAAGAGTTGTCTCTCCAGC TGG	This paper	N/A
dhrs7-sgRNA-2: CGATCCACATCTCCTTCACA CGG	This paper	N/A
dhrs7-sgRNA1-genotyping-F: CGTTTAGAGGTAAAGTGGTGTGG	This paper	N/A
dhrs7-sgRNA1-genotyping-R: ATGCGCGGTCCATTAAATCC	This paper	N/A
dhrs7-sgRNA2-genotyping-F: CGCATAAGATGTCTACAGAGCG	This paper	N/A
dhrs7-sgRNA2-genotyping-R: ACCACTCCAGCTTTGAAGTTCT	This paper	N/A
psbtet-*hsDHRS7*-eGFP-F: ACCCTCGAAAGGCCTCTGAGGCCACCATGAACTGGGAGCTGCTGCT	This paper	N/A
psbtet-*hsDHRS7*-eGFP-R: ATGGAAGCTTGGCCTGACAGGCCTCAGTGATGATGATGATGAT	This paper	N/A
psbtet-*drDhrs7*-eGFP-F: ACCCTCGAAAGGCCTCTGAGGCCACCATGGATATATGCTGGCTGTG	This paper	N/A
psbtet-*drDhrs7*-eGFP-R: ATGGAAGCTTGGCCTGACAGGCCTCAGTGATGATGATGATGAT (same with hsDHRS7-eGFP)	This paper	N/A
psbtet-*hsDHRSB*-eGFP-F: ACCCTCGAAAGGCCTCTGAGGCCACCATGGTCTCTCCGGCTACC	This paper	N/A
psbtet-*hsDHRSB*-eGFP-R: ATGGAAGCTTGGCCTGACAGGCCTCAGTGATGATGATGATGAT (same with hsDHRS7-eGFP)	This paper	N/A
psbtet-*hsDHRSC*-eGFP-F: ACCCTCGAAAGGCCTCTGAGGCCACCATGGGAGTCATGGCCATG	This paper	N/A
psbtet-*hsDHRSC*-eGFP-R: ATGGAAGCTTGGCCTGACAGGCCTCAGTGATGATGATGATGAT (same with hsDHRS7-eGFP)	This paper	N/A
psbtet-*hsDHRS9*-eGFP-F: ACCCTCGAAAGGCCTCTGAGGCCACCATGCTCTTTTGGGTGCTAGG	This paper	N/A
psbtet-*hsDHRS9*-eGFP-R: ATGGAAGCTTGGCCTGACAGGCCTCAGTGATGATGATGATGAT (same with hsDHRS7-eGFP)	This paper	N/A
psbtet-*hsRDH11*-eGFP-F: ACCCTCGAAAGGCCTCTGAGGCCACCATGGTTGAGCTCATGTTC	This paper	N/A
psbtet-*hsRDH11*-eGFP-R: ATGGAAGCTTGGCCTGACAGGCCTCAGTGATGATGATGATGAT (same with hsDHRS7-eGFP)	This paper	N/A
shLMNA: TCACTGTAGATGTTCTTCTGGA	This paper	N/A
shHSD17B12: TTGGTTGCAGAATAGATGGTCA	This paper	N/A
shPECR: TGATATTGACGATAGATCCTCCT	This paper	N/A
shCBR1: TTTGTTTTCATCGTCACTTCAG	This paper	N/A
shRDH14: TTCTTAAACTGAGTTTCATCCA	This paper	N/A
shTPR: TTCTATTTCAAAATACTGTTGT	This paper	N/A
shTM7SF2: TTCATGTAGAGAAAGAGGCTGA	This paper	N/A
shRDH13: TTGATTAGAATGTCCACTCGCT	This paper	N/A

shPYGB: TCTTATTCTGGAACTTCTCTGG	This paper	N/A
shLYPLA2: TTCTCAAGAAATTCCTTCACAG	This paper	N/A
shDHRS1: TTGAACATATACTGCAGGCTTC	This paper	N/A
shALDH2: TTCTTAAACTGAGTTTCATCCA	This paper	N/A
shHSD17B4: TTGGGTGATTCTTTTGTCTTAC	This paper	N/A
shDHRS3: TTGATGTGTTGGGACTTGAGGA	This paper	N/A
shDHRS7_1: TTCACAGTAACAATCTTTCCTT	This paper	N/A
shDHRS7_2: TTAGCTTTCTGTAGACATCCAA	This paper	N/A
DHRS7-sgRNA-3: CTCCTCACCAATTCCACTCG AGG	This paper	N/A
pCW57tet-hsDHRS7-F: ctggagaattggctagcgaattcgccaccATGAACTGGGAGCTGCTGCT	This paper	N/A
pCW57tet-hsDHRS7-R: caaccccaaccccggatccTCAGTGATGATGATGATGAT	This paper	N/A
OXER1-sgRNA-1: GAGGTAGTAGTCCACGCGGA GGG	This paper	N/A
OXER1-sgRNA-2: AACACCGTGTTGGAGGTCCA GGG	This paper	N/A
OXER1-sgRNA-3: ACTCAACCGCTACCTGAAGG TGG	This paper	N/A
OXER1-QPCR-F1 (5' Primer): GGTGTTTCTCAGTGGCTGC	This paper	N/A
OXER1-QPCR-R1 (5' Primer): TTATGAAGTTCCATGGGCTGCT	This paper	N/A
OXER1-QPCR-F2 (CDS): CACTGGCGCTCATCCTCTTT	This paper	N/A
OXER1-QPCR-R2 (CDS): GTTGGTAGGAGCTCTCGTCG	This paper	N/A
OXER1-QPCR-F3 (3' Primer): TTCCATGGTGGCTTTCTGGC	This paper	N/A
OXER1-QPCR-R3 (3' Primer): GTCCAGTTGGTTGAGGCACC	This paper	N/A
ACTB-QPCR-F: CATGTACGTTGCTATCCAGGC	This paper	N/A
ACTB-QPCR-R: CTCCTTAATGTCACGCACGAT	This paper	N/A
Non-targeting-control-sgRNA-1: Gccccgccgccctcccctcc	PMID:26780180	Addgene#80263
Non-targeting-control-sgRNA-2: Tcagcaaaggacgaaacaaa	PMID:26780180	Addgene#80261
Non-targeting-control-sgRNA-3: Ccagttgctctgggggaaca	PMID:26780180	Addgene#80262
nudt1-gRNA-1: GTCGACACCCTCCACAAGAT TGG	This paper	N/A
nudt1-gRNA-2: TTATCAATGTCGAACCACTG CGG	This paper	N/A
nudt15-gRNA-1: ACTCCAGGACGCTTGAGGAT GGG	This paper	N/A
nudt15-gRNA-2: GTAGGTGCCTTTCCCAACTC TGG	This paper	N/A
nudt15-gRNA-3: AAAGGCACCTACCAGCTACC AGG	This paper	N/A
nudt15-gRNA-4: GATGTTCTTTAGATGGATAC CGG	This paper	N/A
nudt1-gRNA-1-genotyping-F: GTCAATCTTCCCGCTTTTATTG	This paper	N/A
nudt1-gRNA-1-genotyping-R: TGGCTCTCCTTCATAGTTGTCA	This paper	N/A
nudt1-gRNA-2-genotyping-F: TATTGGGGAAACCGAACTAATG	This paper	N/A
nudt1-gRNA-2-genotyping-R: CAGAGGAAACCACAAGATGTCA	This paper	N/A
nudt15-gRNA-1-genotyping-F: GGTGGGTCATTCCTGAATTAGA	This paper	N/A
nudt15-gRNA-1-genotyping-R: AAATTCAATGTGACCTCCTGGT	This paper	N/A
nudt15-gRNA-2,3-genotyping-F: CAGTTGAAAATGGAAAACCCAT	This paper	N/A
nudt15-gRNA-2,3-genotyping-R: TGCTCTAGCGCATGTGTAAACT	This paper	N/A
nudt15-gRNA-4-genotyping-F: TCGACATCATCAGAGAGAGCTG	This paper	N/A
nudt15-gRNA-4-genotyping-R: ACAGACTACGCAGTCAACGAAA	This paper	N/A
NUDT1-sgRNA1: CAGATCGTGTTTGAGTTCGT GGG	This paper	N/A

NUDT1-sgRNA2: CATGAAAAAGCGAGGCTTCG GGG	This paper	N/A
NUDT1-sgRNA3: ACTCACCGTCGCTCTCCACG GGG	This paper	N/A
NUDT1-sgRNA4: CGATCTGGCCCACCTTGTGC AGG	This paper	N/A

Supplementary Table 3: Plasmids used in this study.

DNA	Source	Sequence confirmed	Use
psbtet-hsDHRS7-eGFP	This paper	Yes	Co-localization, microsome NADPH assay and LC-MS
psbtet-drDHRS7-eGFP	This paper	Yes	Co-localization, microsome NADPH assay and LC-MS
psbtet-hsDHRS7-3C-eGFP-3*Flag-10*His	This paper	Yes	Protein purification
psbtet-drDHRS7-3C-eGFP-3*Flag-10*His	This paper	Yes	Protein purification
psbtet-hsDHRS7-eGFP-K207E	This paper	Yes	Microsome NADPH assay
psbtet-hsDHRS7-eGFP-Y203F	This paper	Yes	Microsome NADPH assay
psbtet-hsDHRS7-eGFP-S190A	This paper	Yes	Microsome NADPH assay
psbtet-hsDHRS7-eGFP-N162L	This paper	Yes	Microsome NADPH assay
psbtet-hsDHRS7-eGFP-R82E	This paper	Yes	Microsome NADPH assay
psbtet-mmDHRS7-eGFP	This paper	Yes	Microsome NADPH assay
psbtet-rnDHRS7-eGFP	This paper	Yes	Microsome NADPH assay
psbtet-btDHRS7-eGFP	This paper	Yes	Microsome NADPH assay
lentiCRISPRv2-puro-DHRS7-sgRNA1	This paper	Yes	Caco-2 cells knockdown, LC-MS
lentiCRISPRv2-puro-DHRS7-sgRNA3	This paper	Yes	Caco-2 cells knockdown, LC-MS
lentiCRISPRv2-puro-non-targeting-sgRNA1	This paper	Yes	Caco-2 cells knockdown, LC-MS
lentiCRISPRv2-puro-non-targeting-sgRNA2	This paper	Yes	Caco-2 cells knockdown, LC-MS
lentiCRISPRv2-puro-non-targeting-sgRNA3	This paper	Yes	Caco-2 cells knockdown, LC-MS
lentiCRISPRv2-puro-OXER1-sgRNA1	This paper	Yes	Caco-2 cells knockdown, Apoptosis

lentiCRISPRv2-puro-OXER1-sgRNA2	This paper	Yes	Caco-2 cells knockdown, Apoptosis
lentiCRISPRv2-puro-OXER1-sgRNA3	This paper	Yes	Caco-2 cells knockdown, Apoptosis
lentiCRISPRv2-puro-NUDT1-sgRNA1	This paper	Yes	Caco-2 cells knockdown, Apoptosis
lentiCRISPRv2-puro-NUDT1-sgRNA2	This paper	Yes	Caco-2 cells knockdown, Apoptosis
lentiCRISPRv2-puro-NUDT1-sgRNA3	This paper	Yes	Caco-2 cells knockdown, Apoptosis
lentiCRISPRv2-puro-NUDT1-sgRNA4	This paper	Yes	Caco-2 cells knockdown, Apoptosis
pCW57-hsDHRS7-EGFP	This paper	Yes	Caco-2 cells overexpression, LC-MS
pDestTol2CG2-cldn15la:QF2	This paper	Yes	Transgenic zebrafish
pDestTol2CR-cldn15la:hsNUDT1	This paper	Yes	Transgenic zebrafish
pDestTol2CR-cldn15la:drNUDT15	This paper	Yes	Transgenic zebrafish
psbtet-hsDHRS7B-eGFP	This paper	Yes	Microsome NADPH assay
psbtet-hsDHRS7C-eGFP	This paper	Yes	Microsome NADPH assay
psbtet-hsDHRS9-eGFP	This paper	Yes	Microsome NADPH assay
psbtet-hsRDH11-eGFP	This paper	Yes	Microsome NADPH assay
psbtet-hs15-PGDH-eGFP	This paper	Yes	Microsome NADPH assay

Figure 1c

IB: DHRS7 IB: β-actin

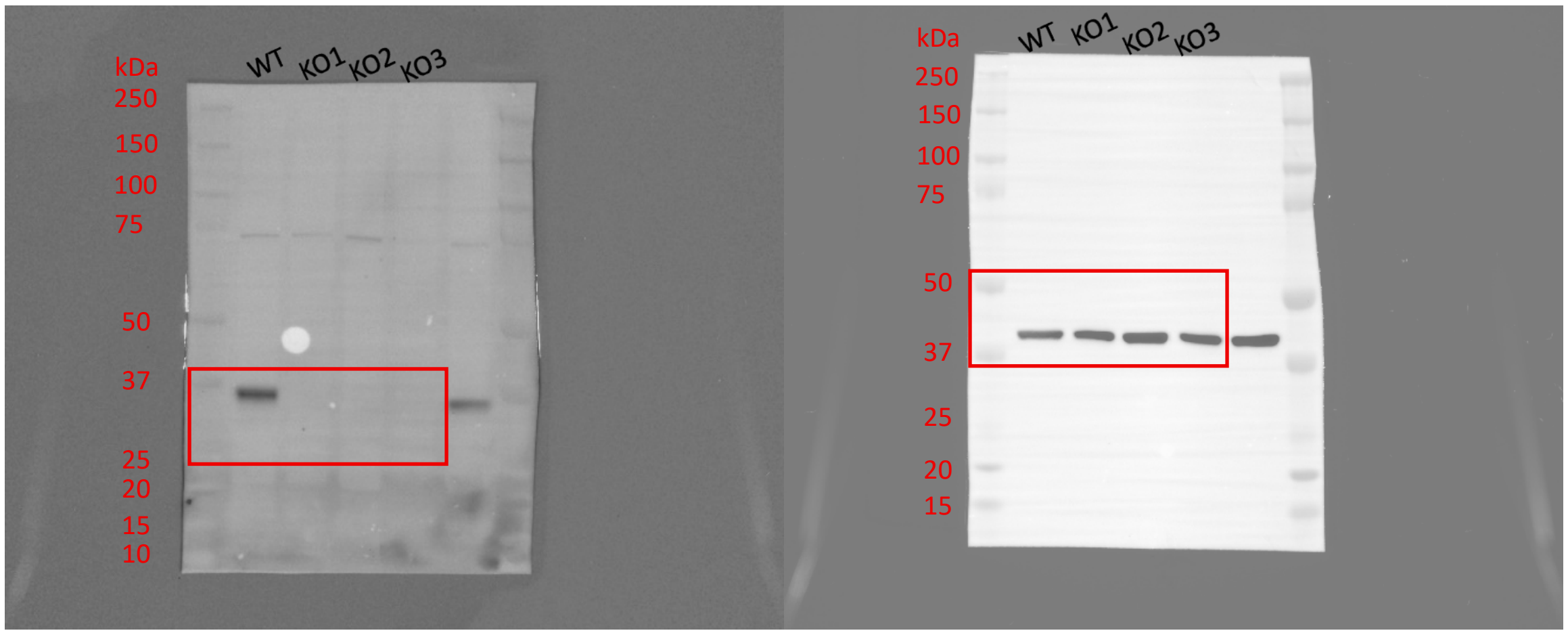

Samples used for figure were divided across two gels.

Figure 1g

IB: DHRS7 IB: β-actin

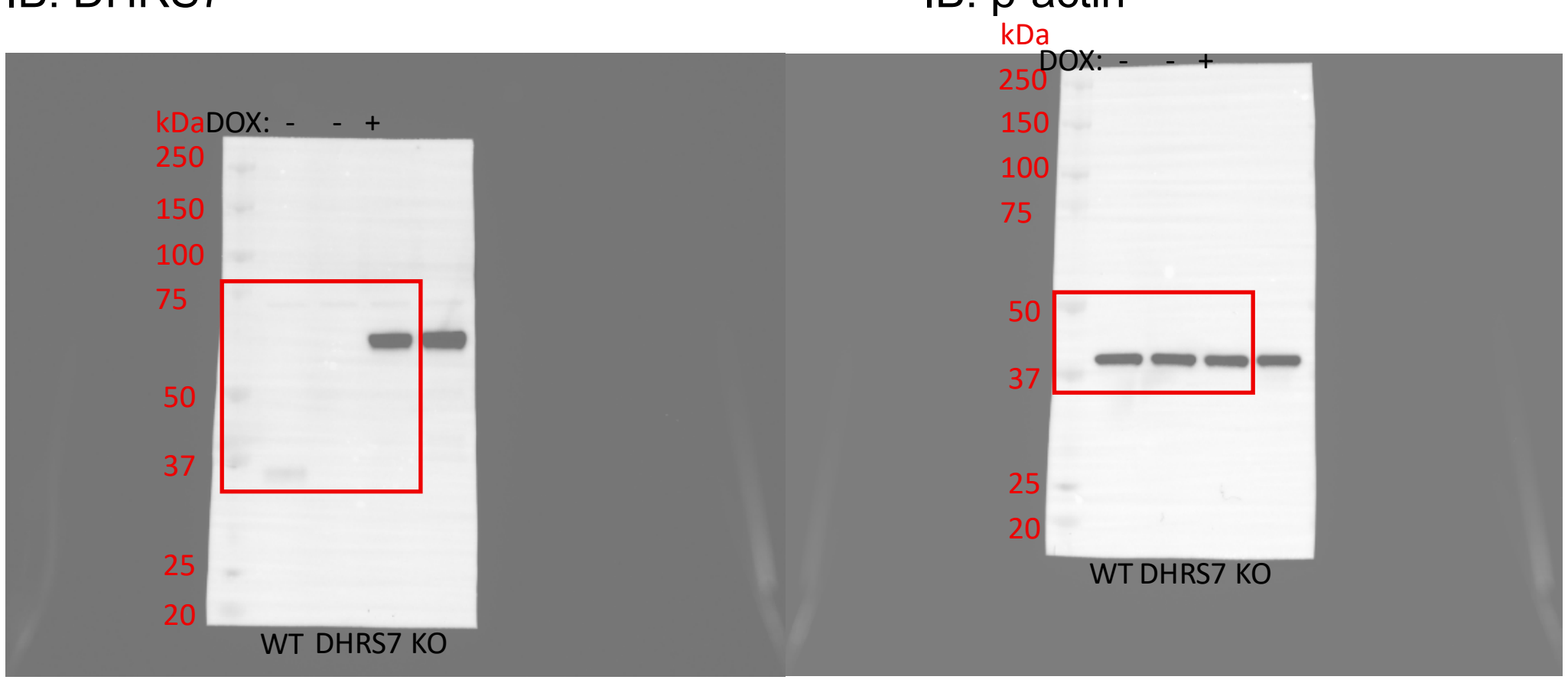

Samples used for figure were divided across two gels.

Figure 2b

IB: DHRS7 IB: β-actin

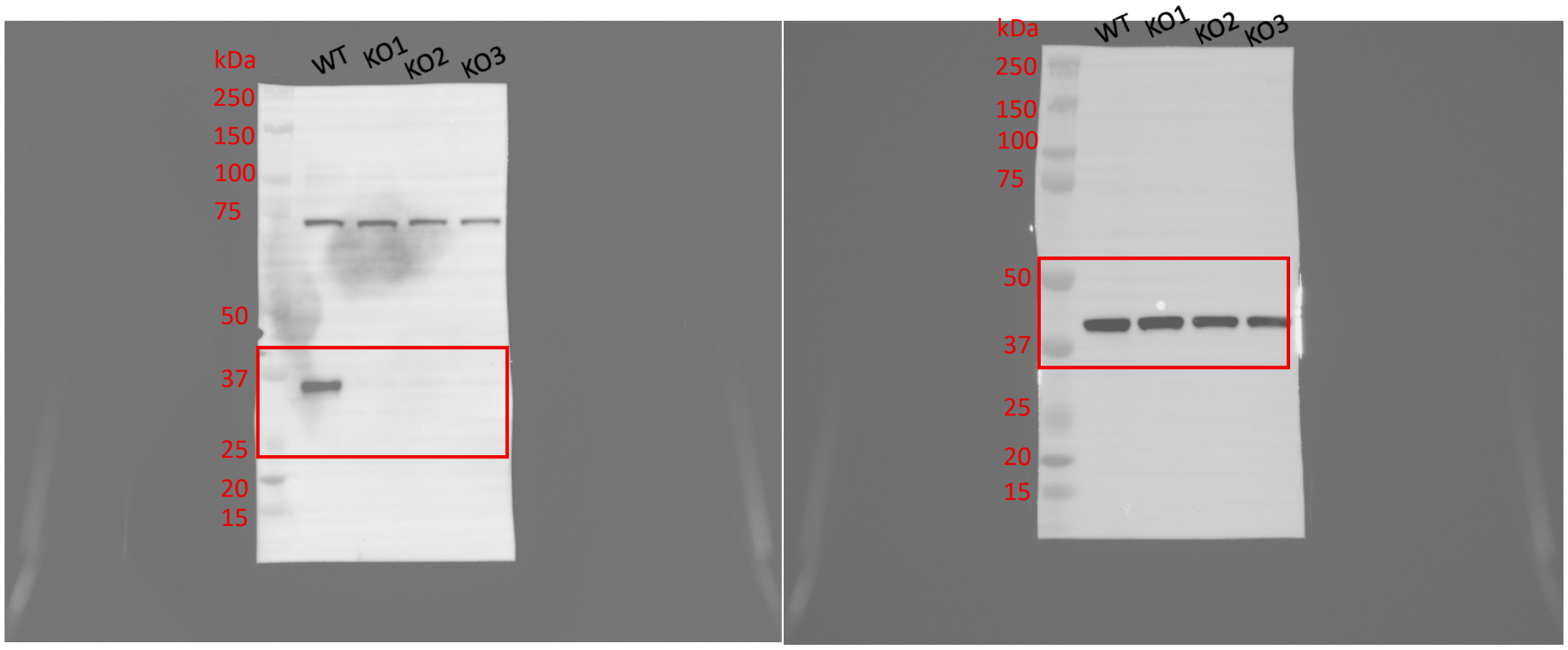

Samples used for figure were divided across two gels.

Figure 2e

IB: EGFP IB: β-actin

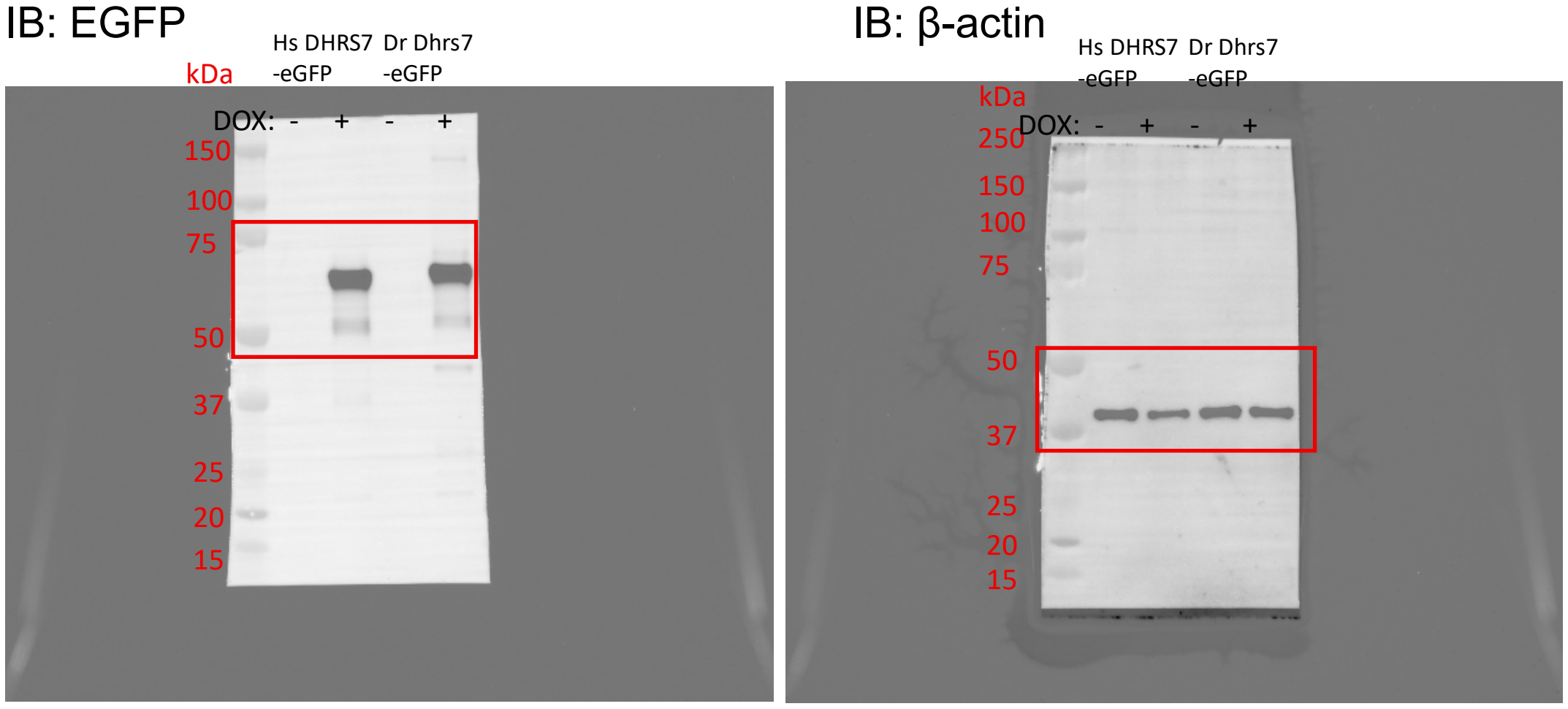

Samples used for figure were divided across two gels.

Figure 2i

IB: EGFP IB: β-actin

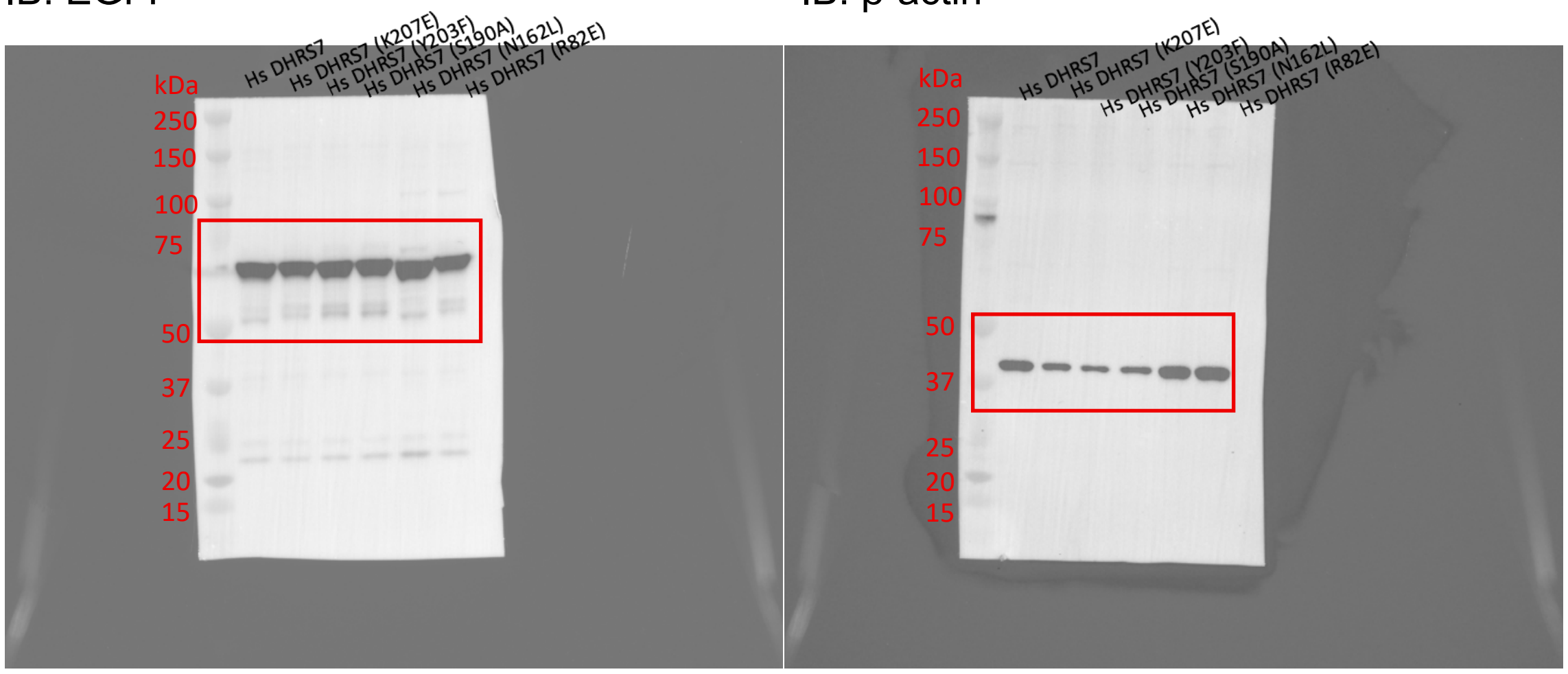

Samples used for figure were divided across two gels.

Figure 2j

IB: EGFP IB: β-actin

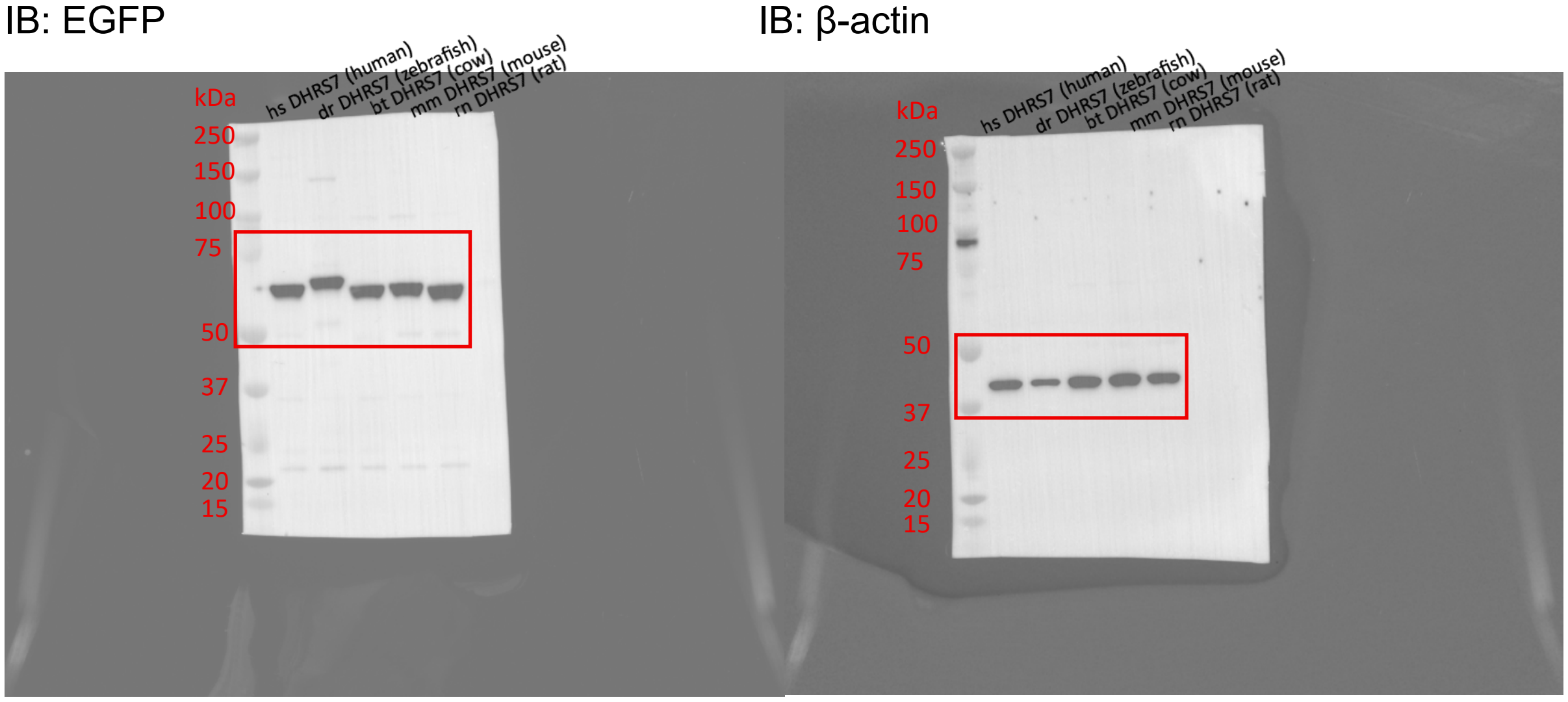

Samples used for figure were divided across two gels.

Extended Data Figure 1c

IB: DHRS7 IB: β-actin

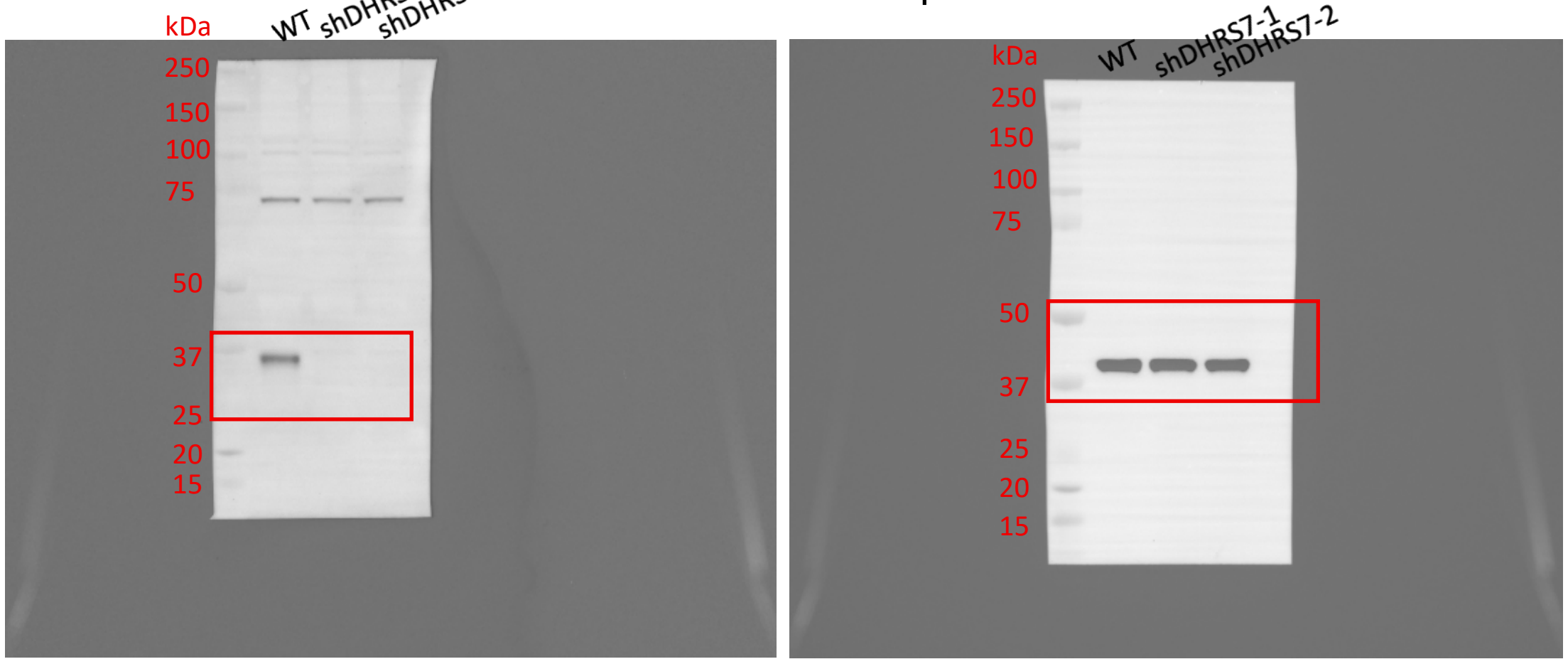

Samples used for figure were divided across two gels.

Extended Data Figure 1f

IB: DHRS7 IB: β-actin

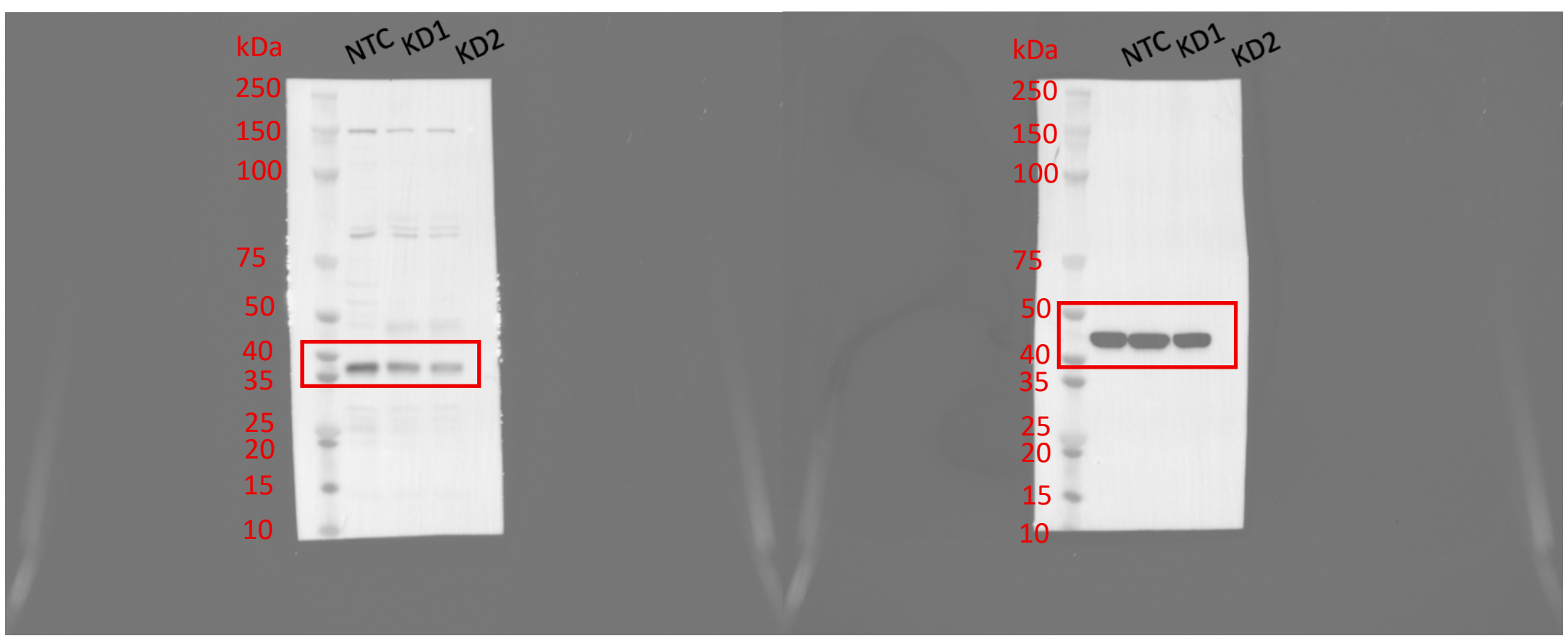

Samples used for figure were divided across two gels.

Extended Data Figure 1g

IB: DHRS7

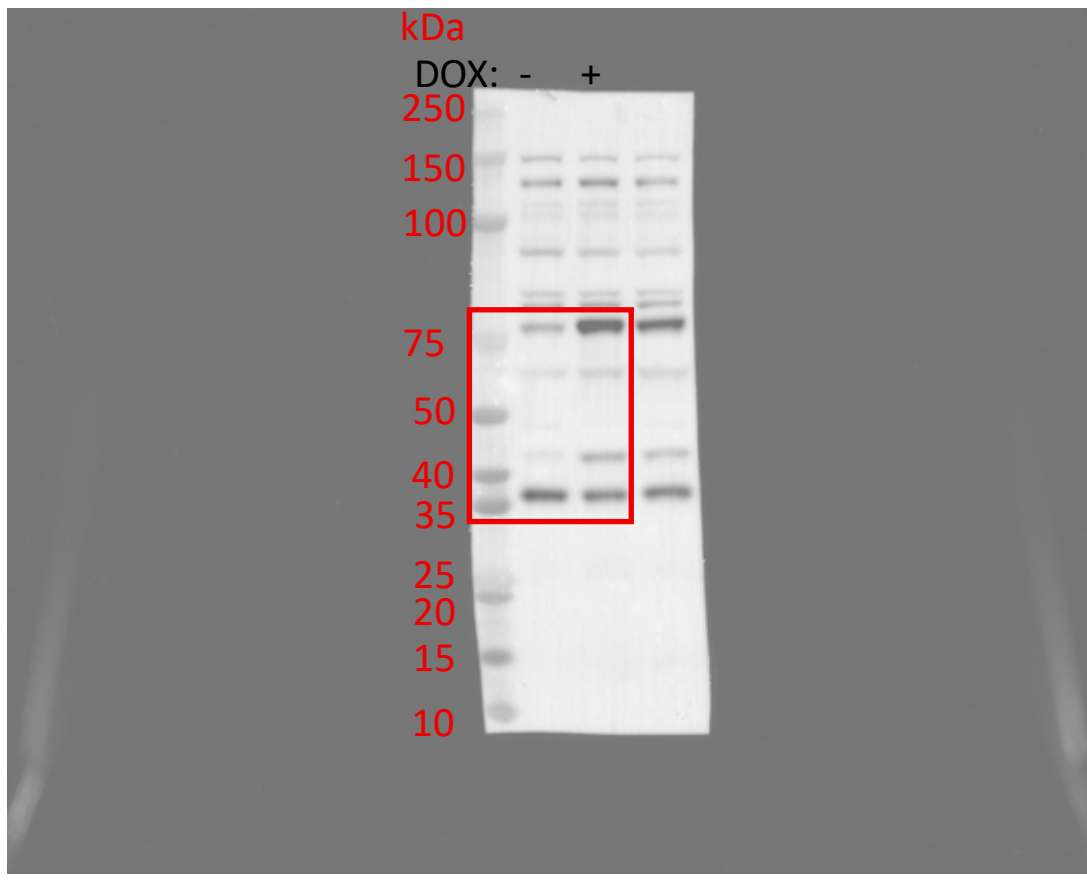

IB: β-actin

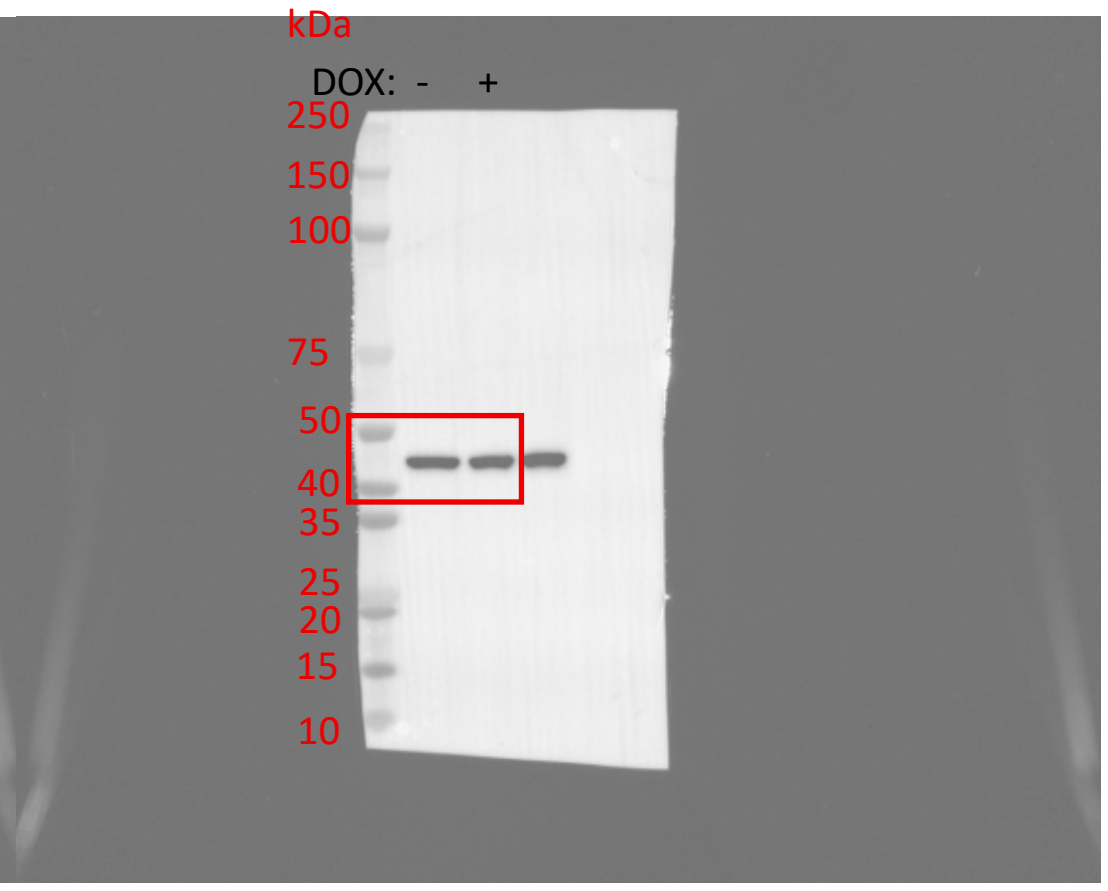

Samples used for figure were divided across two gels.

Extended Data Figure 3b

IB: DHRS7

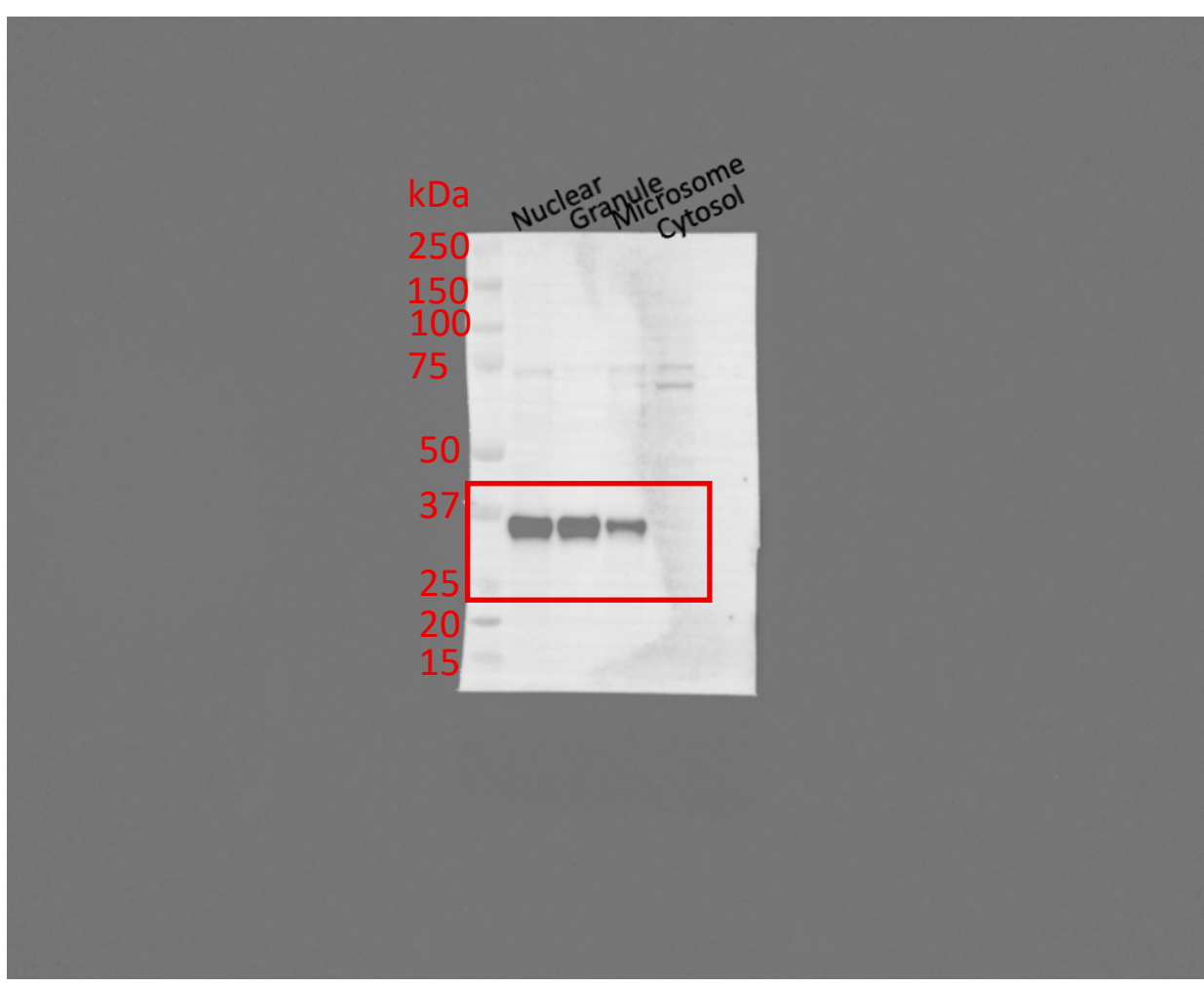

IB: β-actin

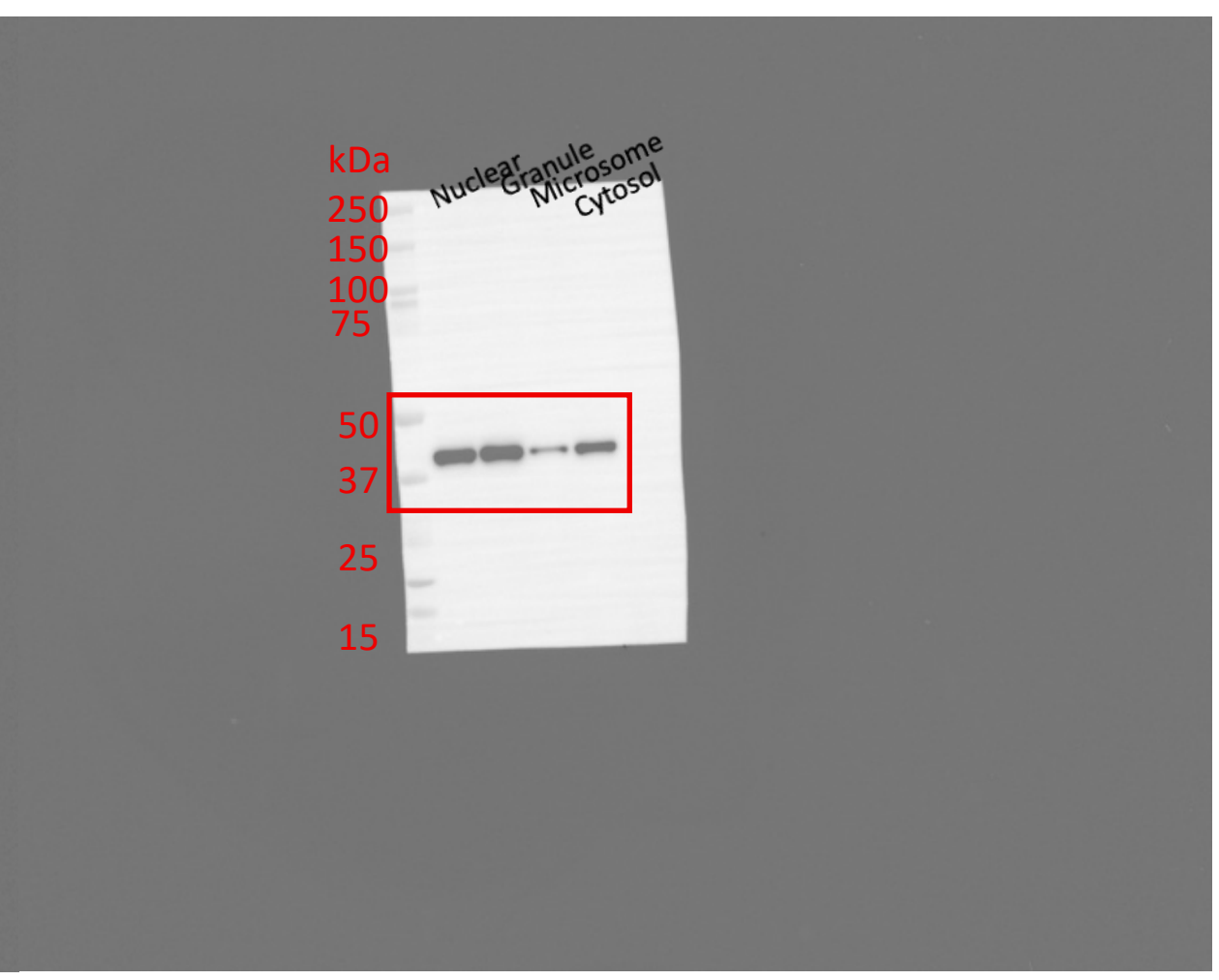

IB: Calnexin (ER)

IB: Cox4 (Mitochondria)

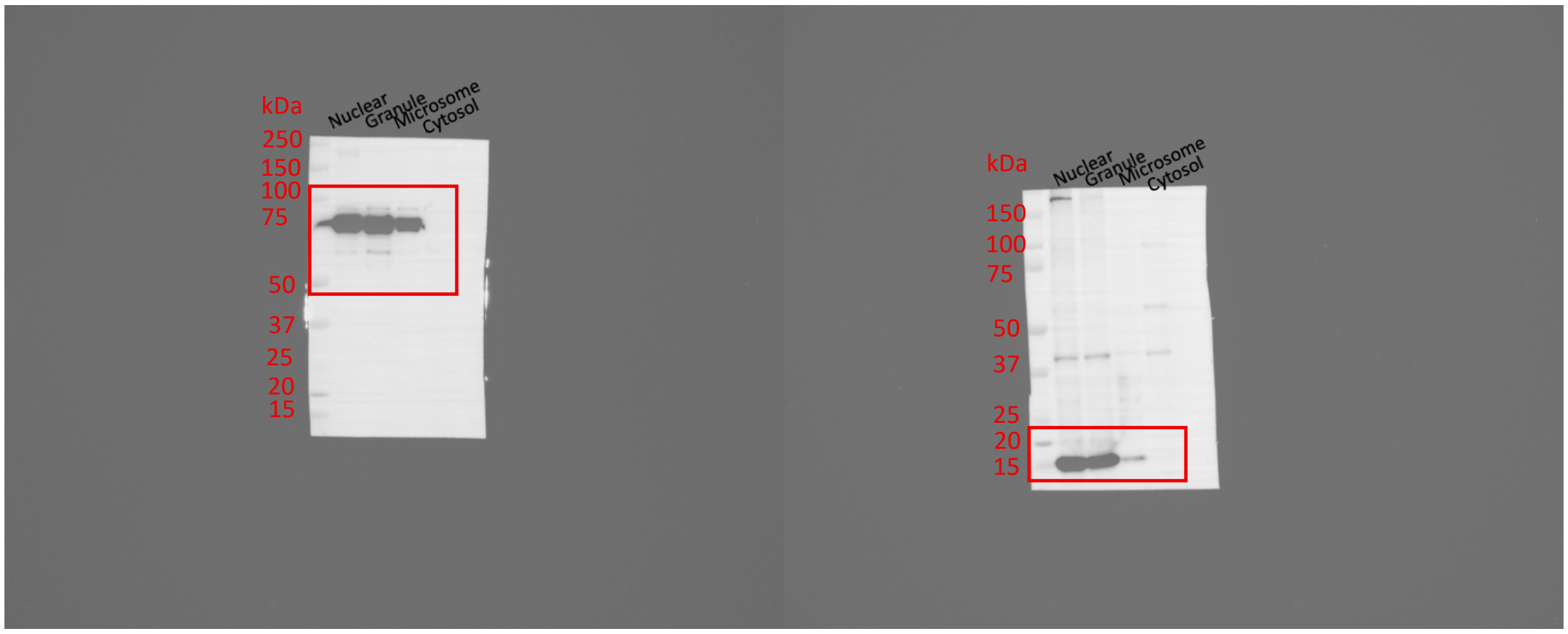

IB: Lamin A/C (Nuclear)

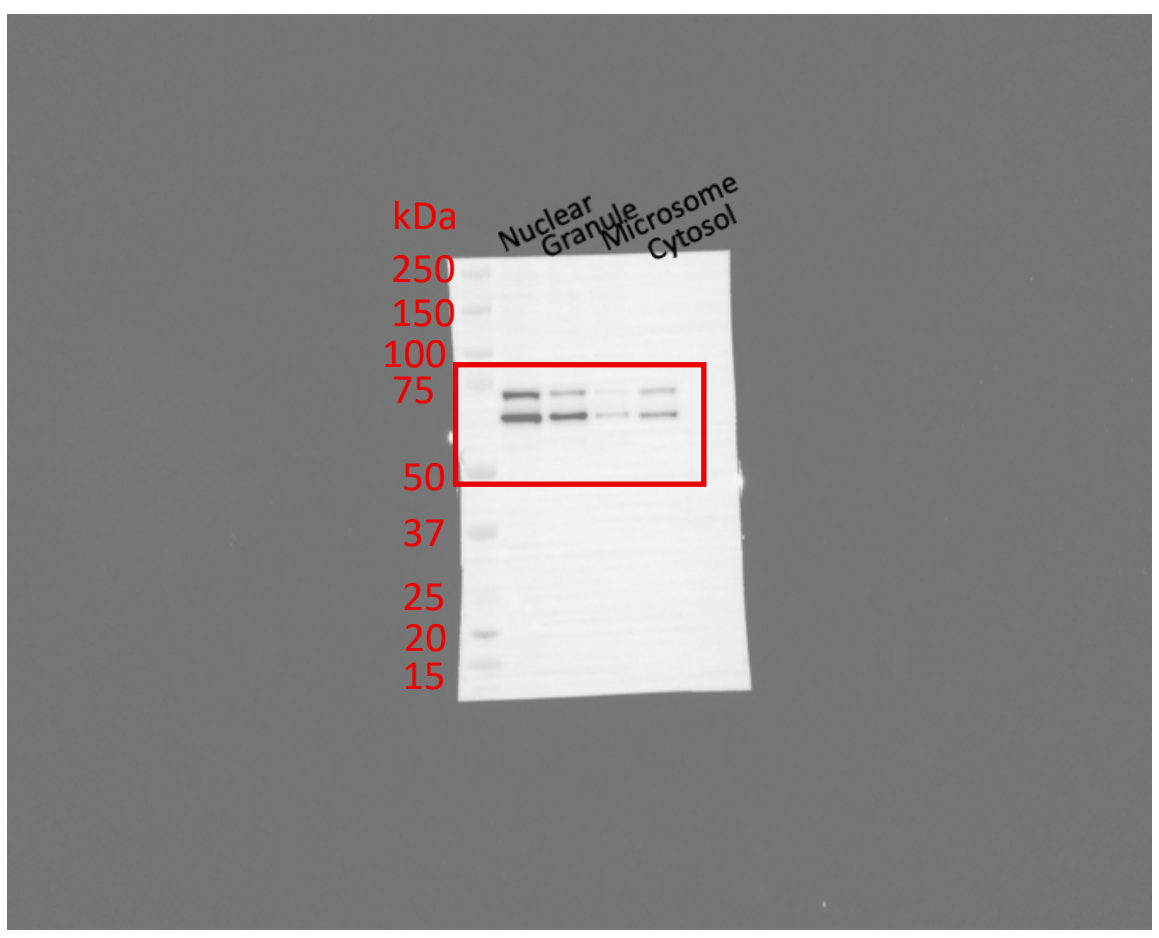

Samples used for figure were divided across five gels.

Extended Data Figure 3i

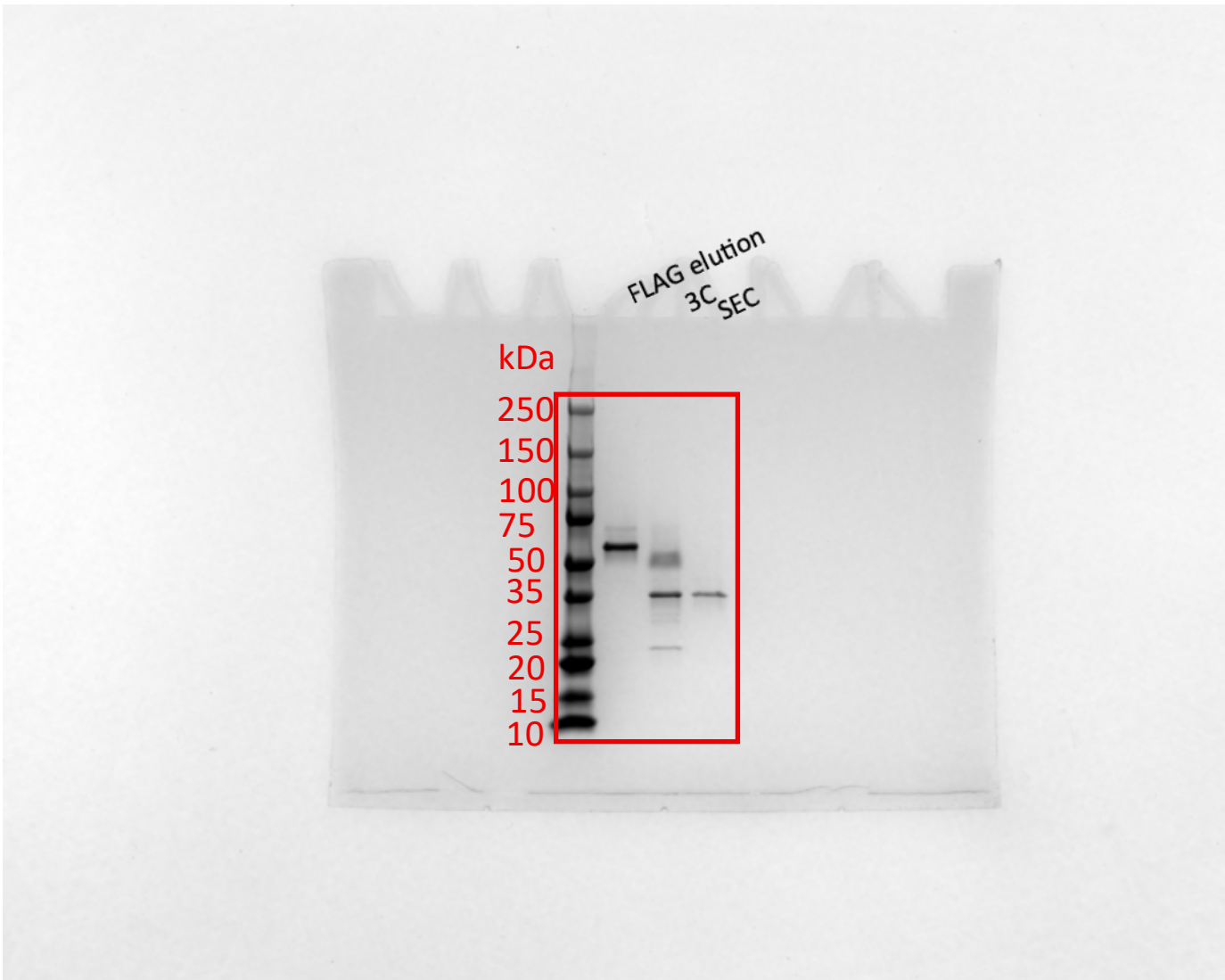

Extended Data Figure 3j

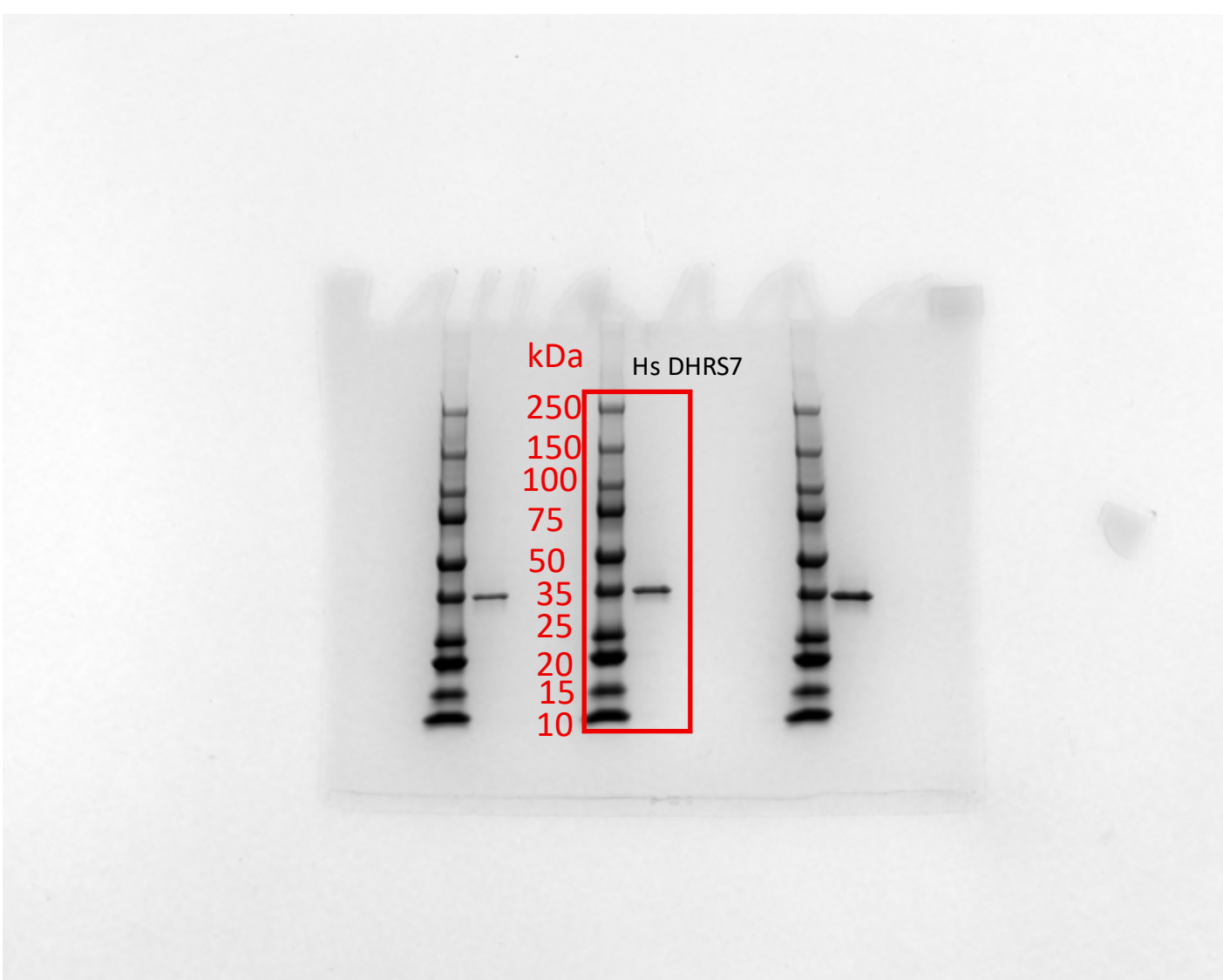

Extended Data Figure 3l

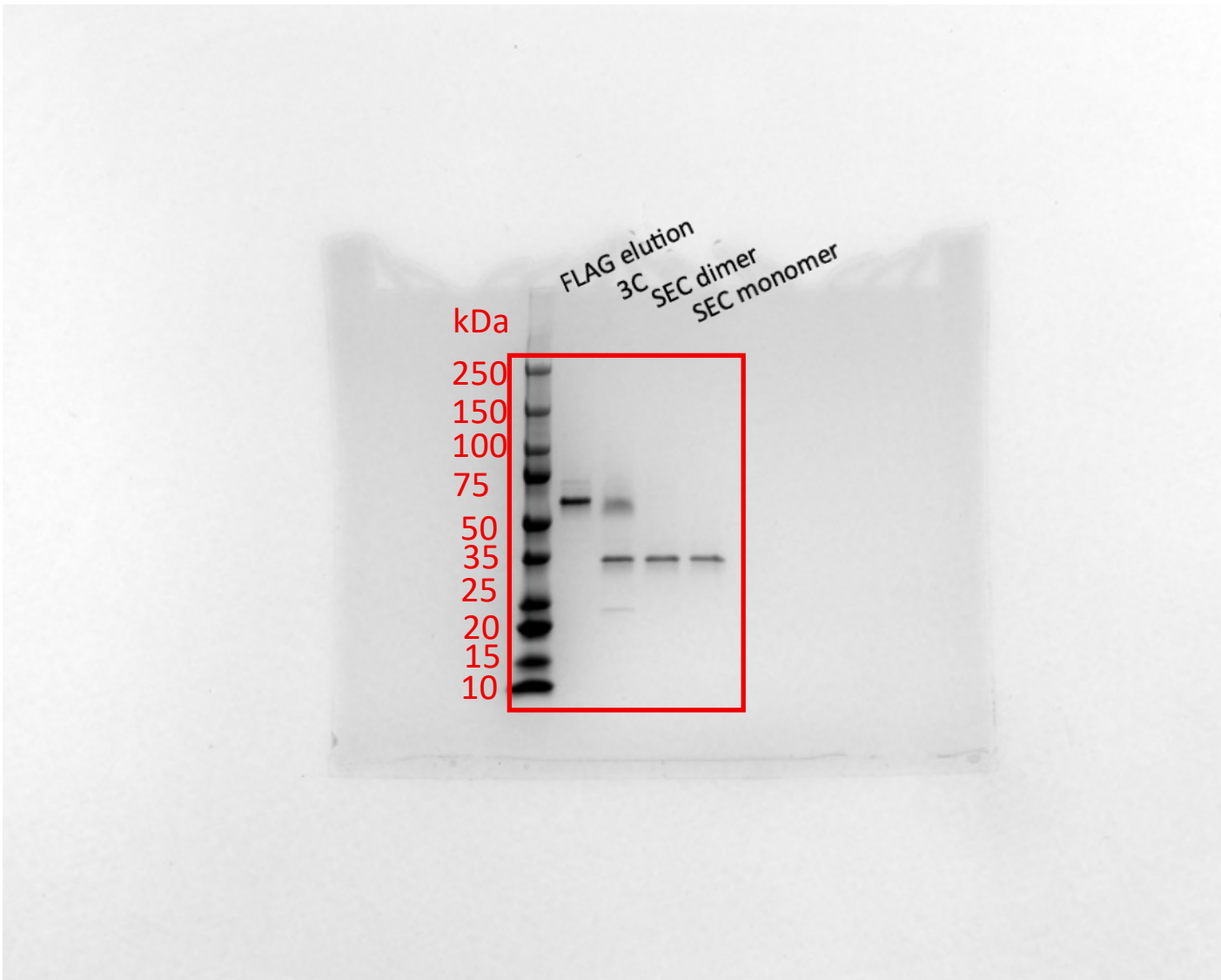

Extended Data Figure 3m

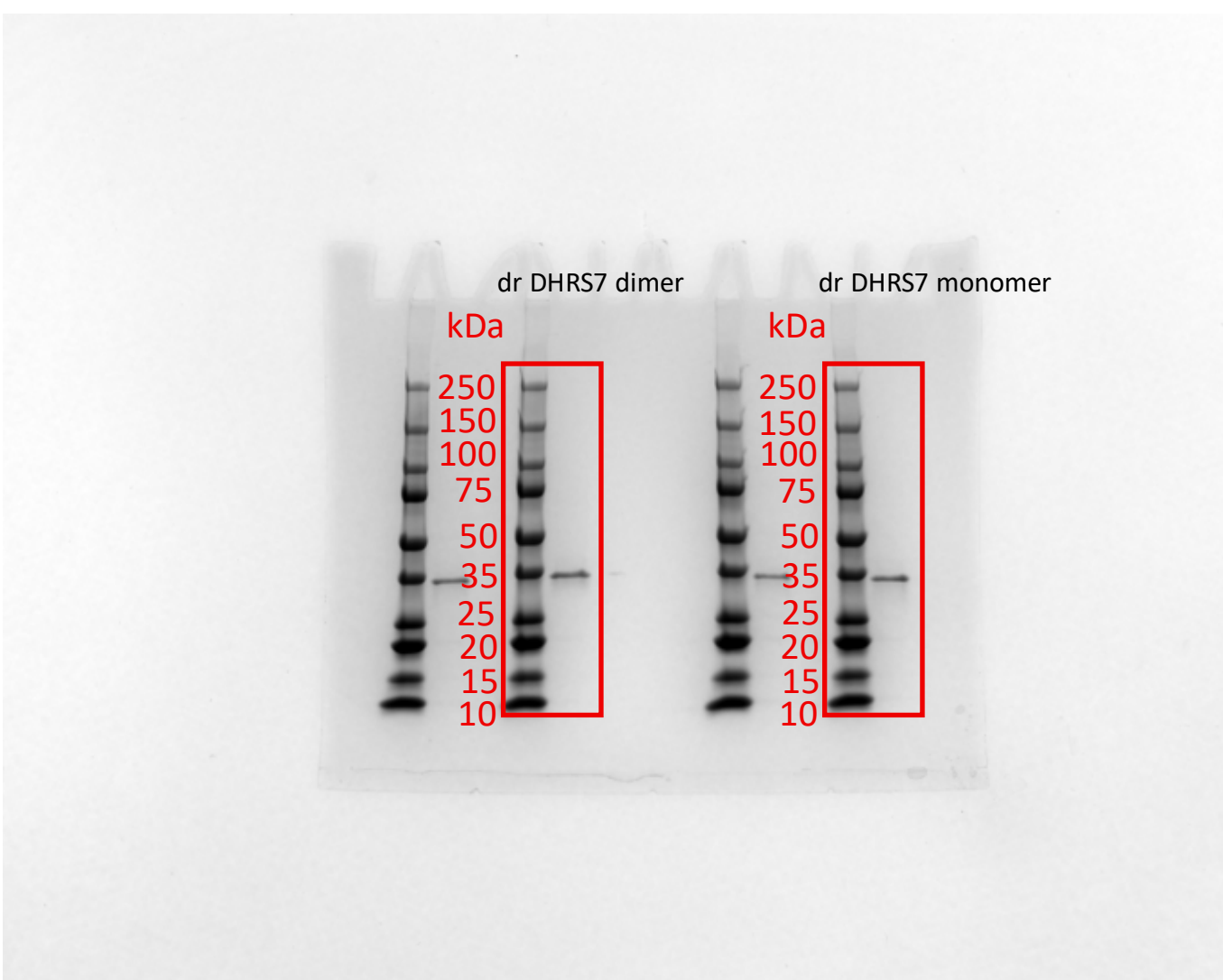

Extended Data Figure 6d:

Genotyping for *dhrs7*^{mk224}

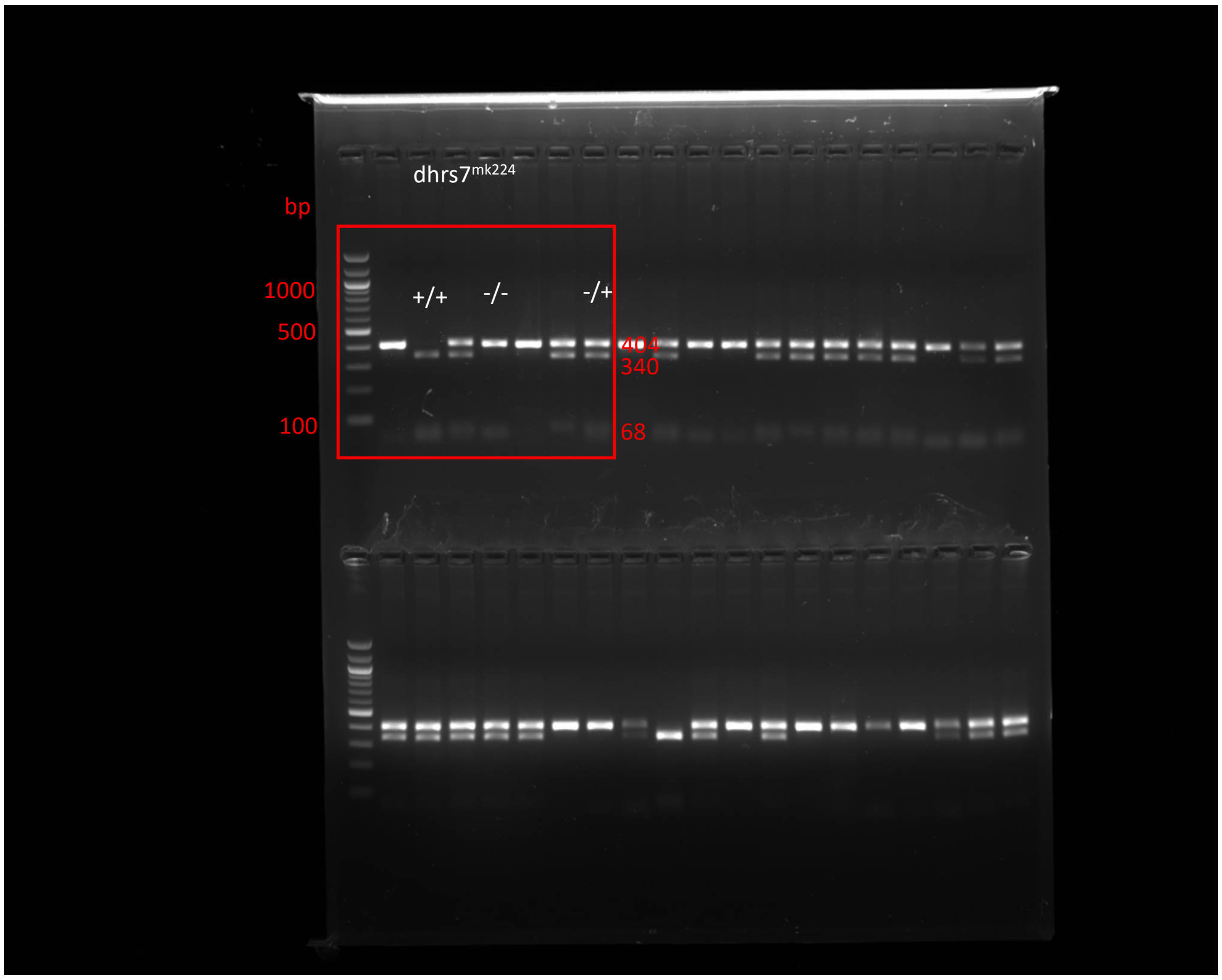

Extended Data Figure 6e:

Genotyping for *dhrs7*^{mk225}

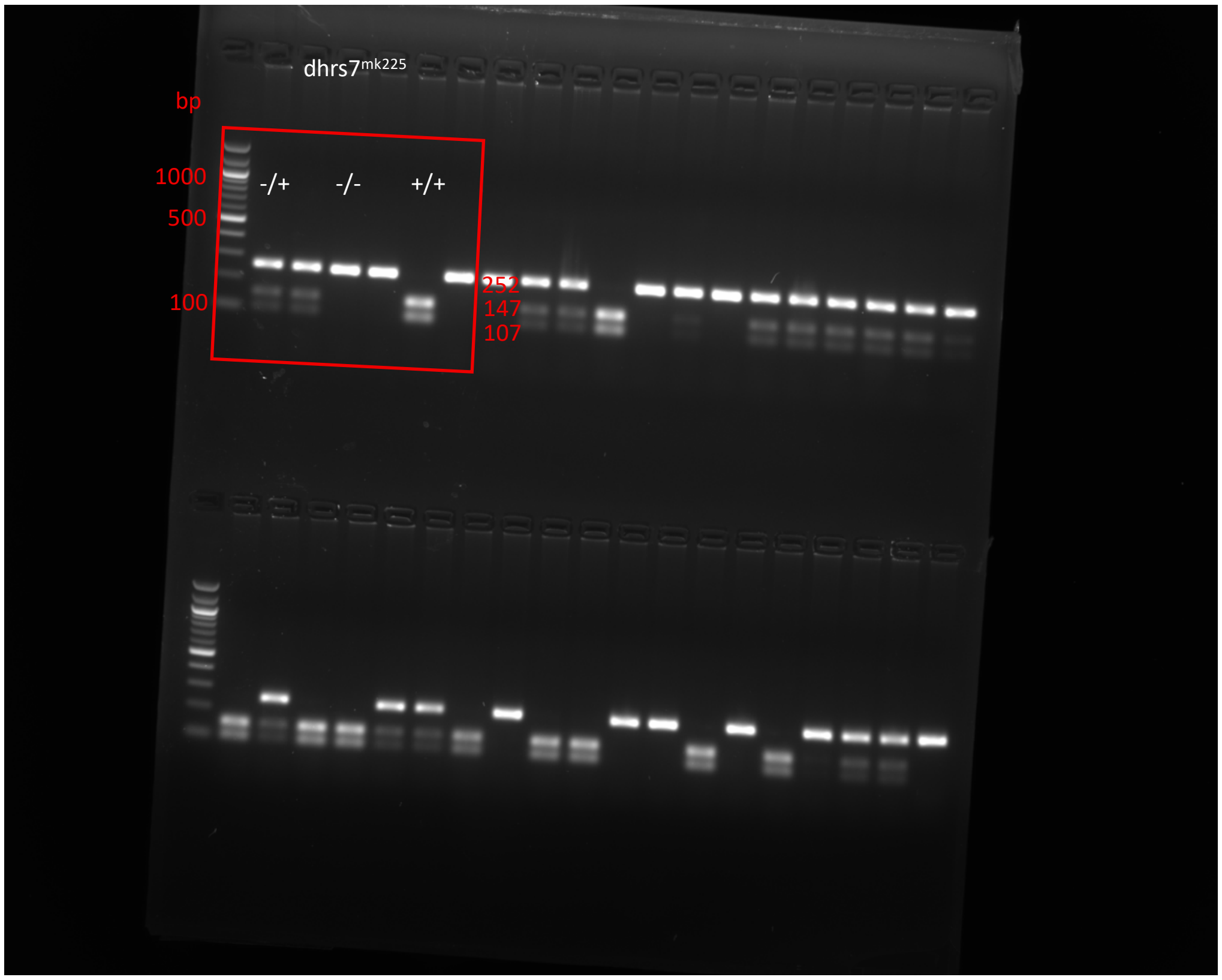

Extended Data Figure 6o:

Genotyping using the primers for *hcar1-4*^{mk214} Genotyping using the primers for *dhrs7*^{mk224}

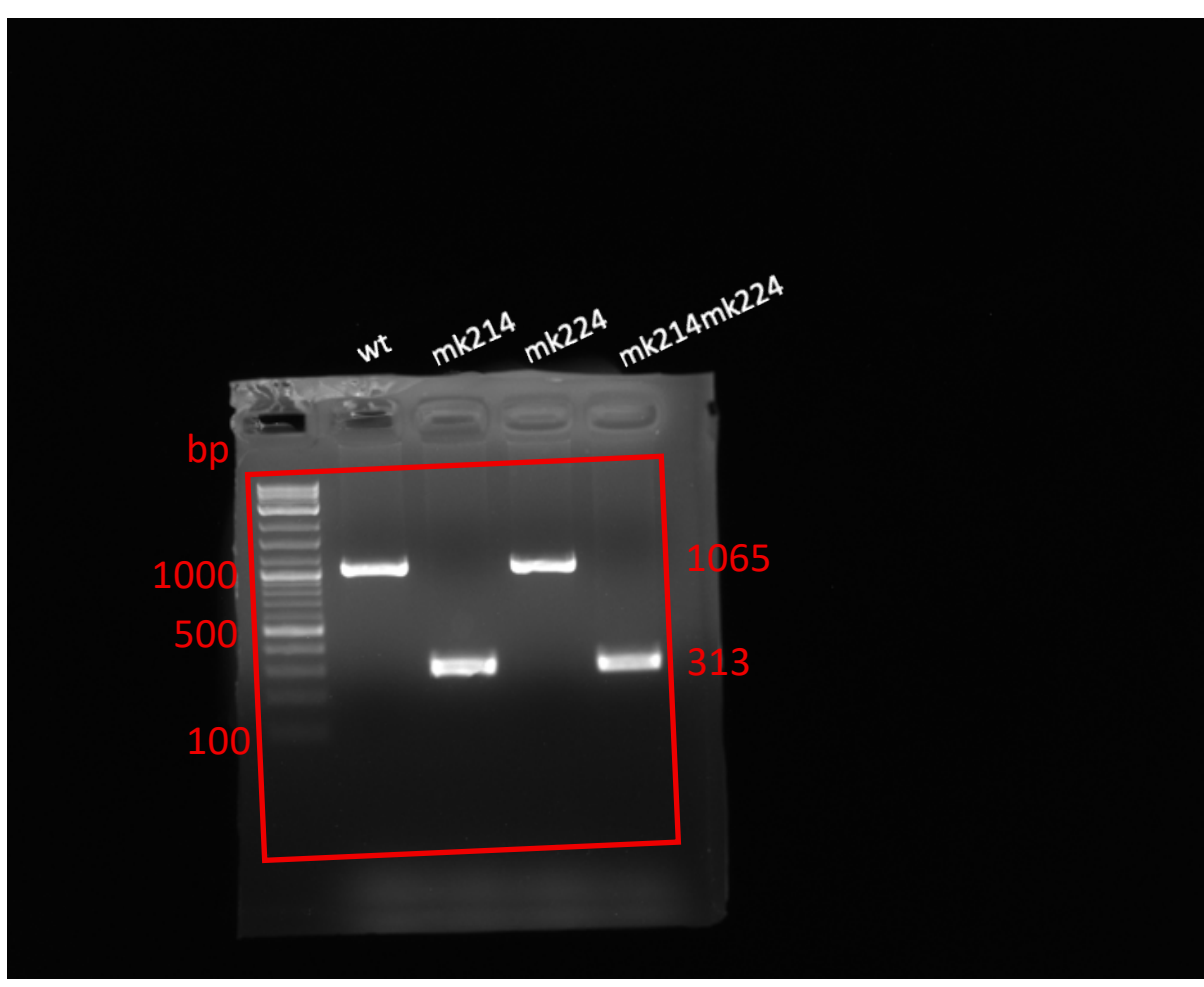

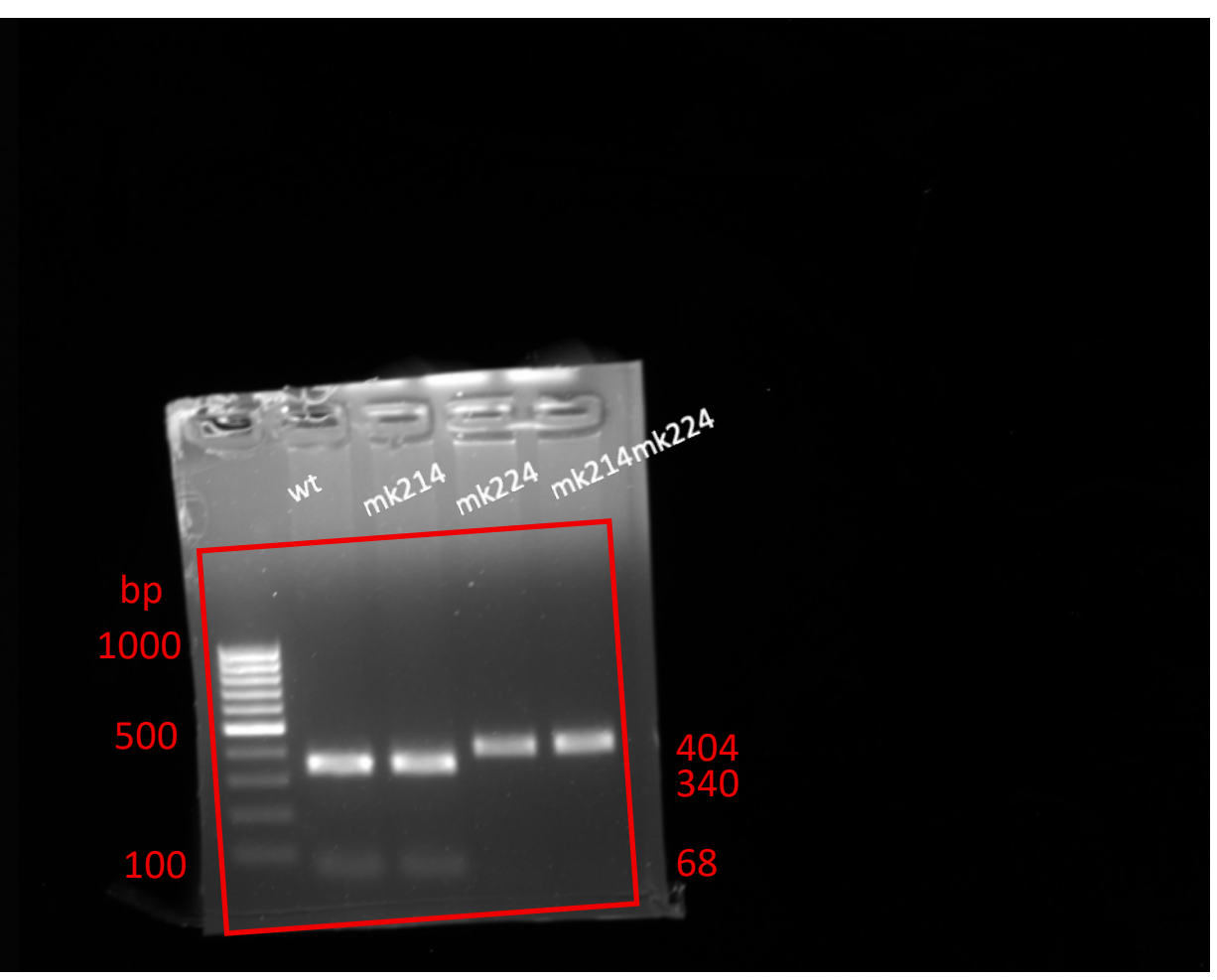

Extended Data Figure 8f:

IB: β-actin, NUDT1

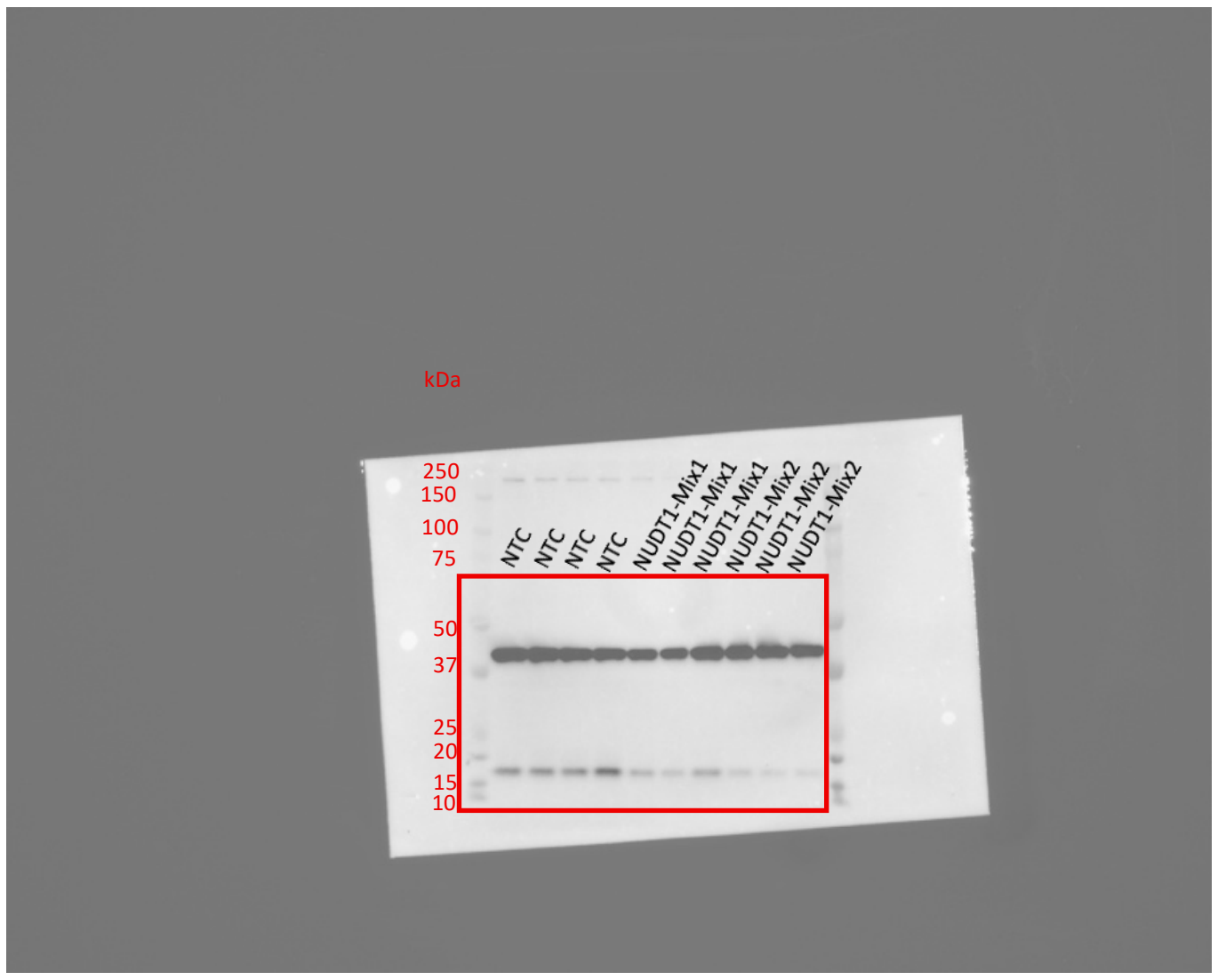

Extended Data Figure 9g:

IB: β-actin, NUDT1

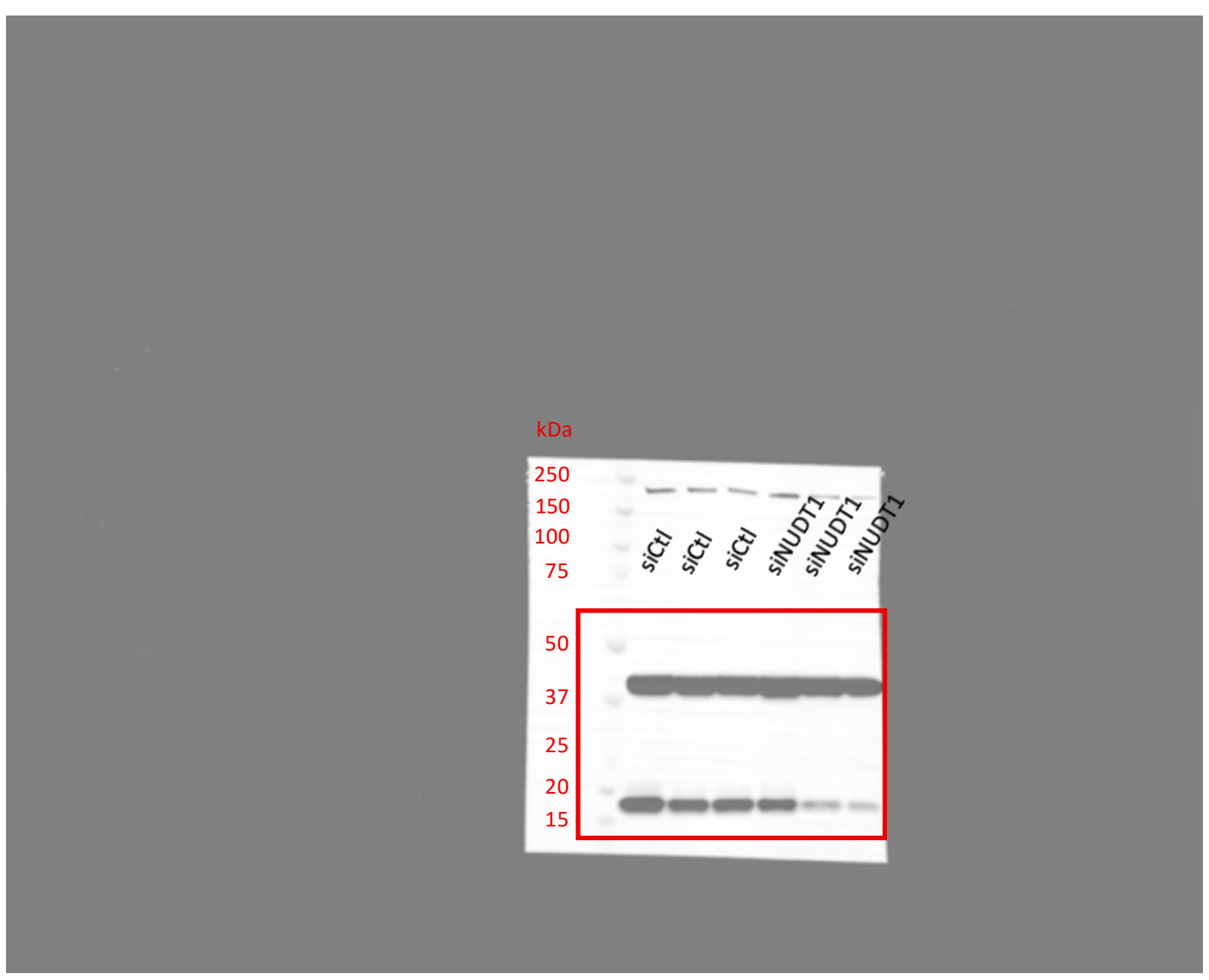

Extended Data Figure 10a:

IB: β-actin, NUDT1

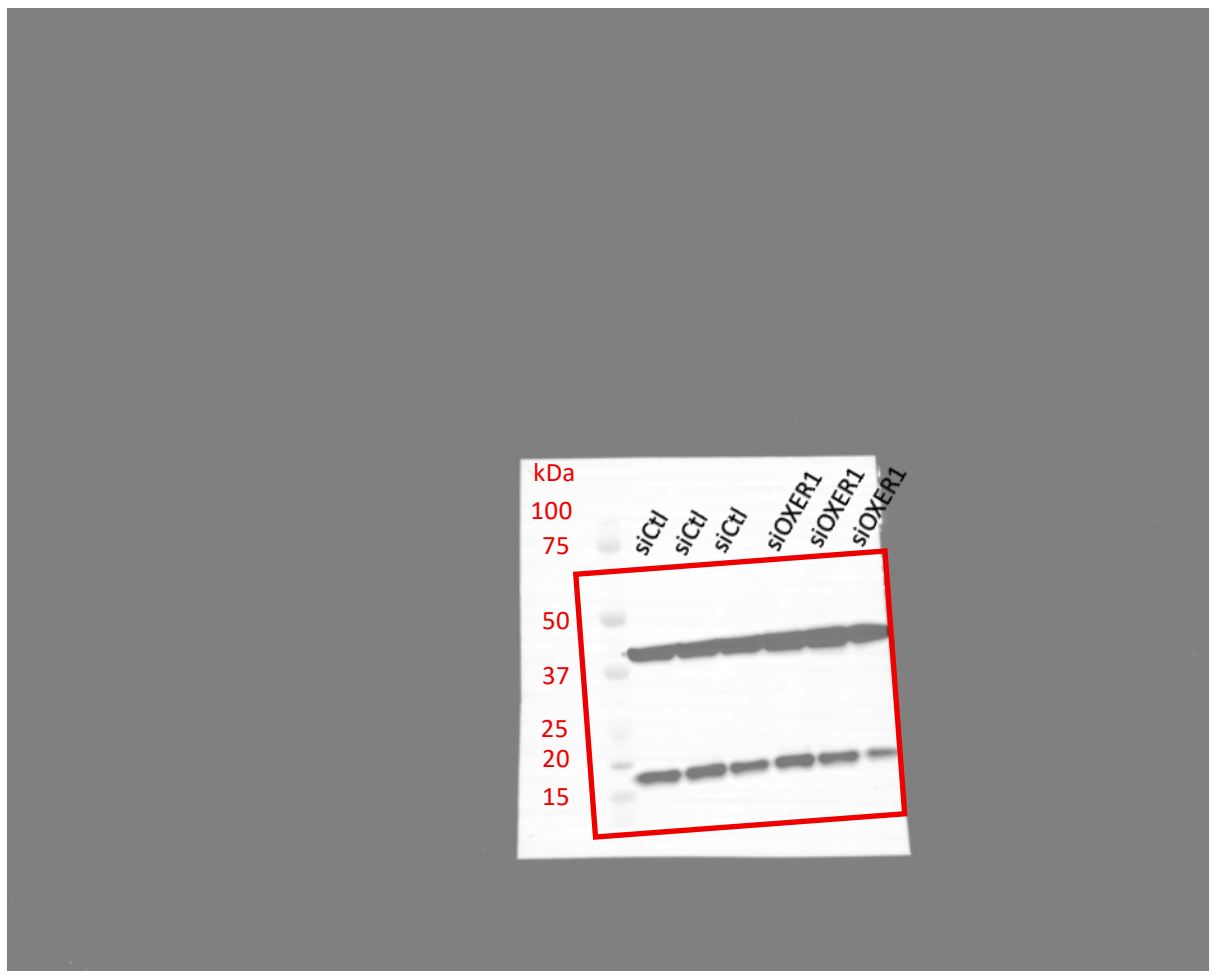

Extended Data Figure 10b:

IB: β-actin, NUDT1

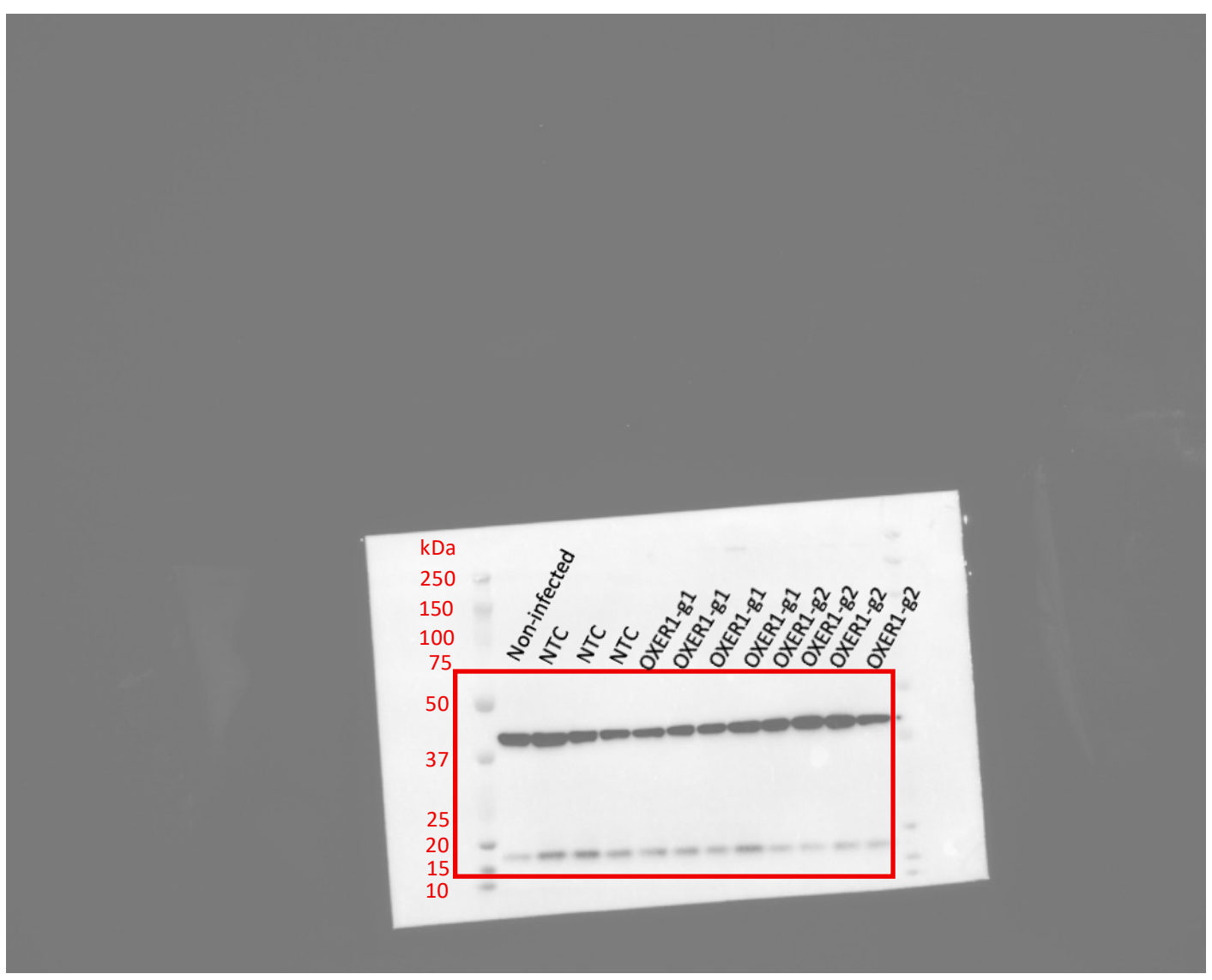

Extended Data Figure 10c:

IB: β-actin, NUDT1

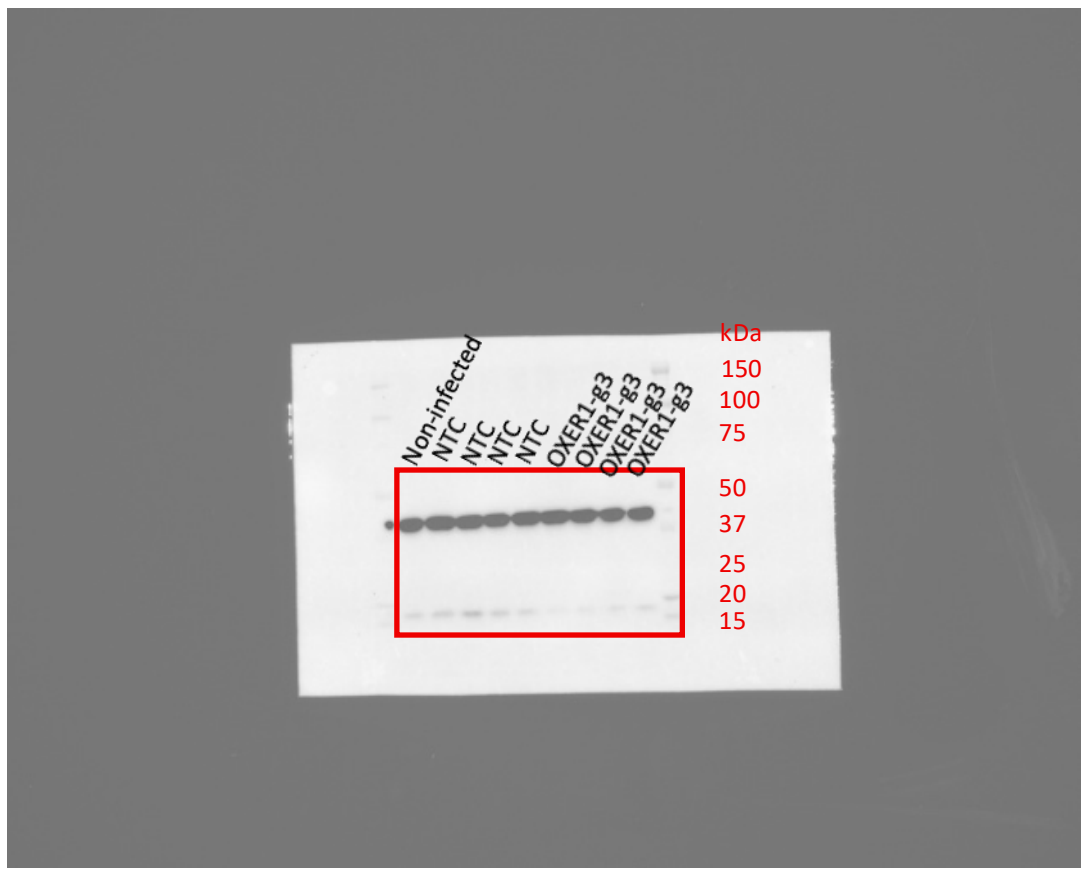

Extended Data Figure 11b:

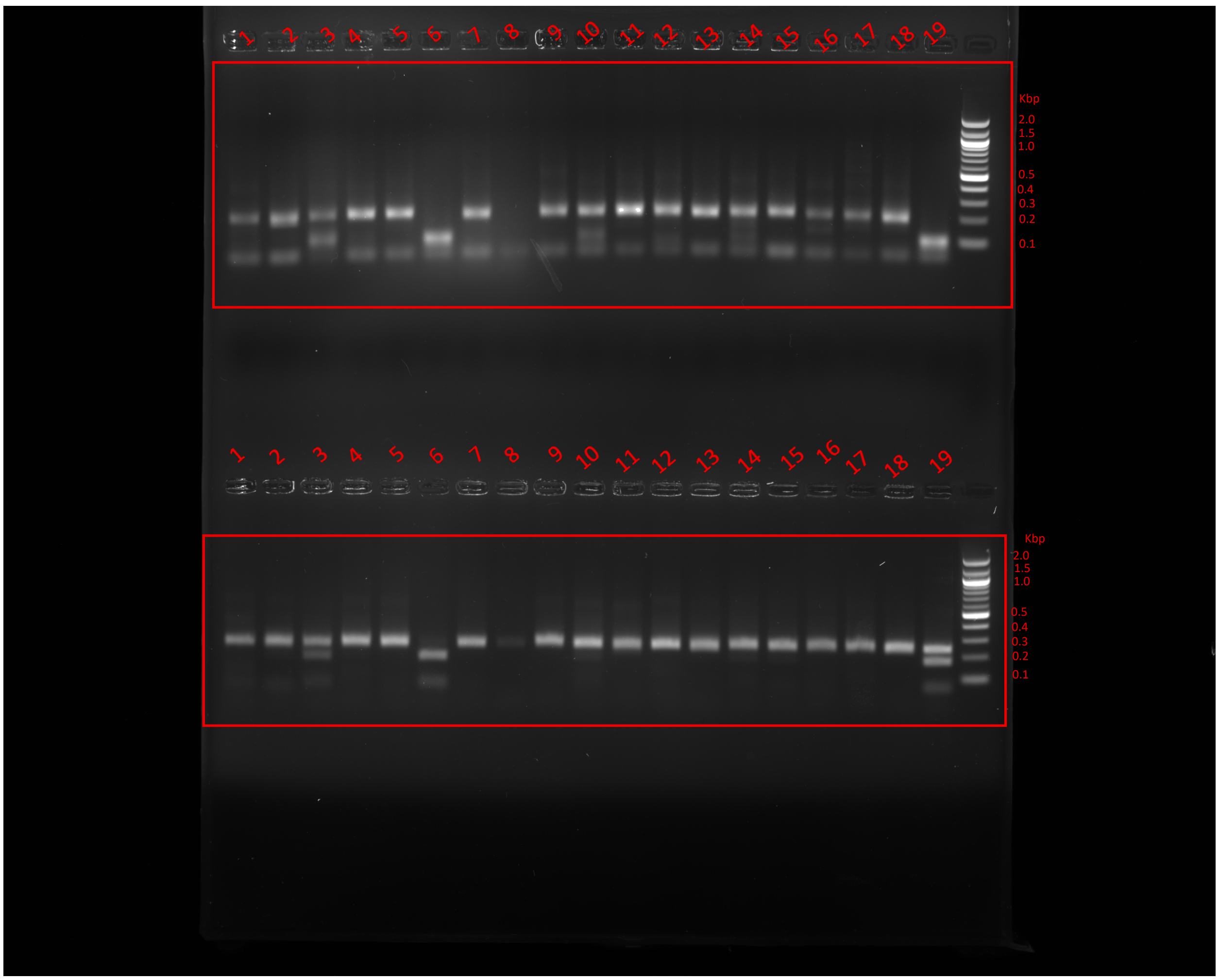

Extended Data Figure 11d:

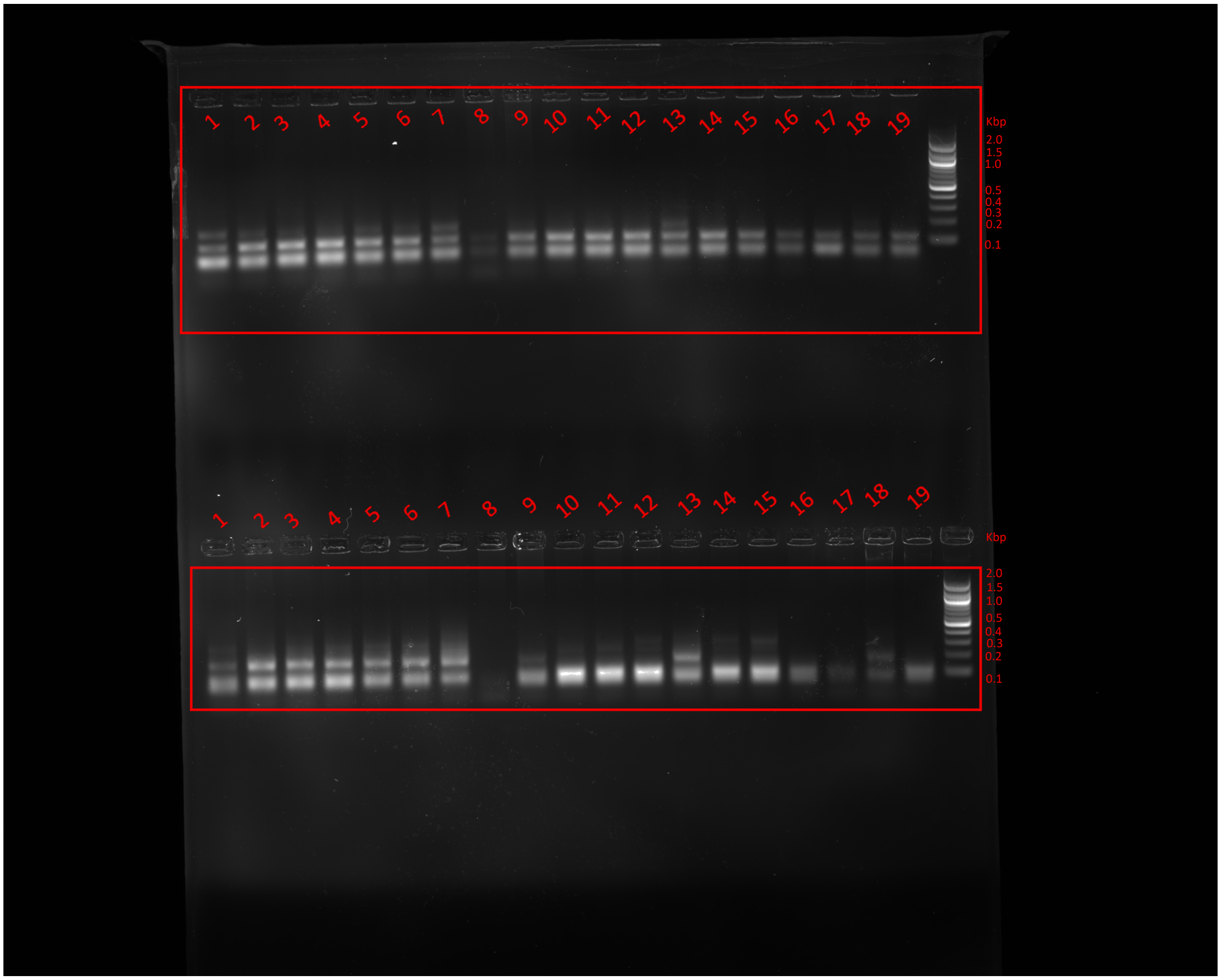

Extended Data Figure 11d:

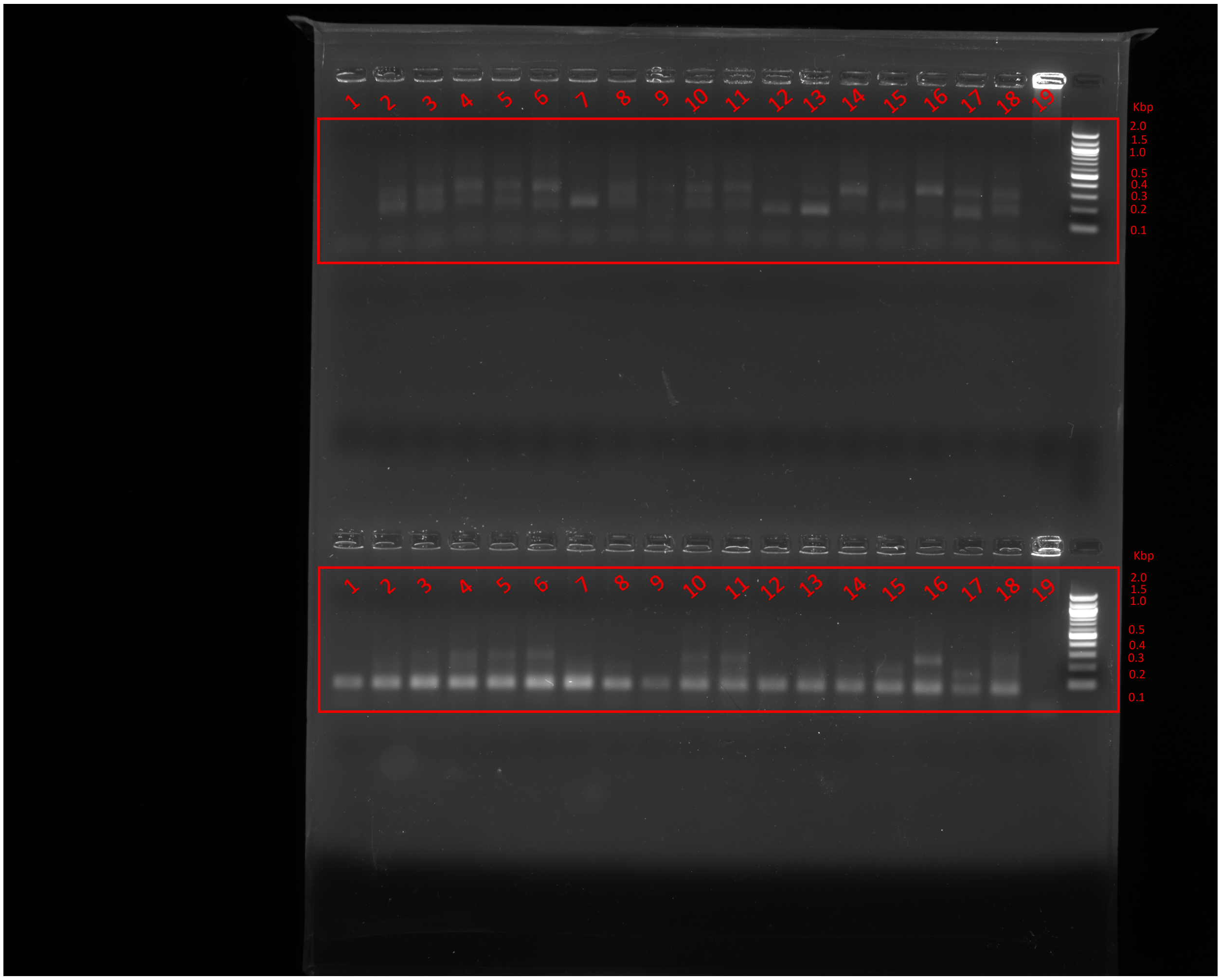

Supplementary Figure 1. Uncropped images of western blots or Coomassie shown in this study. Full length, uncropped and unprocessed scans of all immunoblots, Coomassie-stained SDS-PAGE gels and DNA agarose gel electrophoresis shown in this study. Panels are labeled with the corresponding figure and panel number and the detected target (IB, immunoblot). Molecular weights are indicated in Kilo Daltons (kDa) for protein gels, and DNA fragment sizes are indicated in base pairs (bp) for agarose gels. For immunoblots, membranes were cut, where applicable, based on the positions of molecular weight markers and the expected sizes of the proteins of interest before probing with the corresponding antibodies.

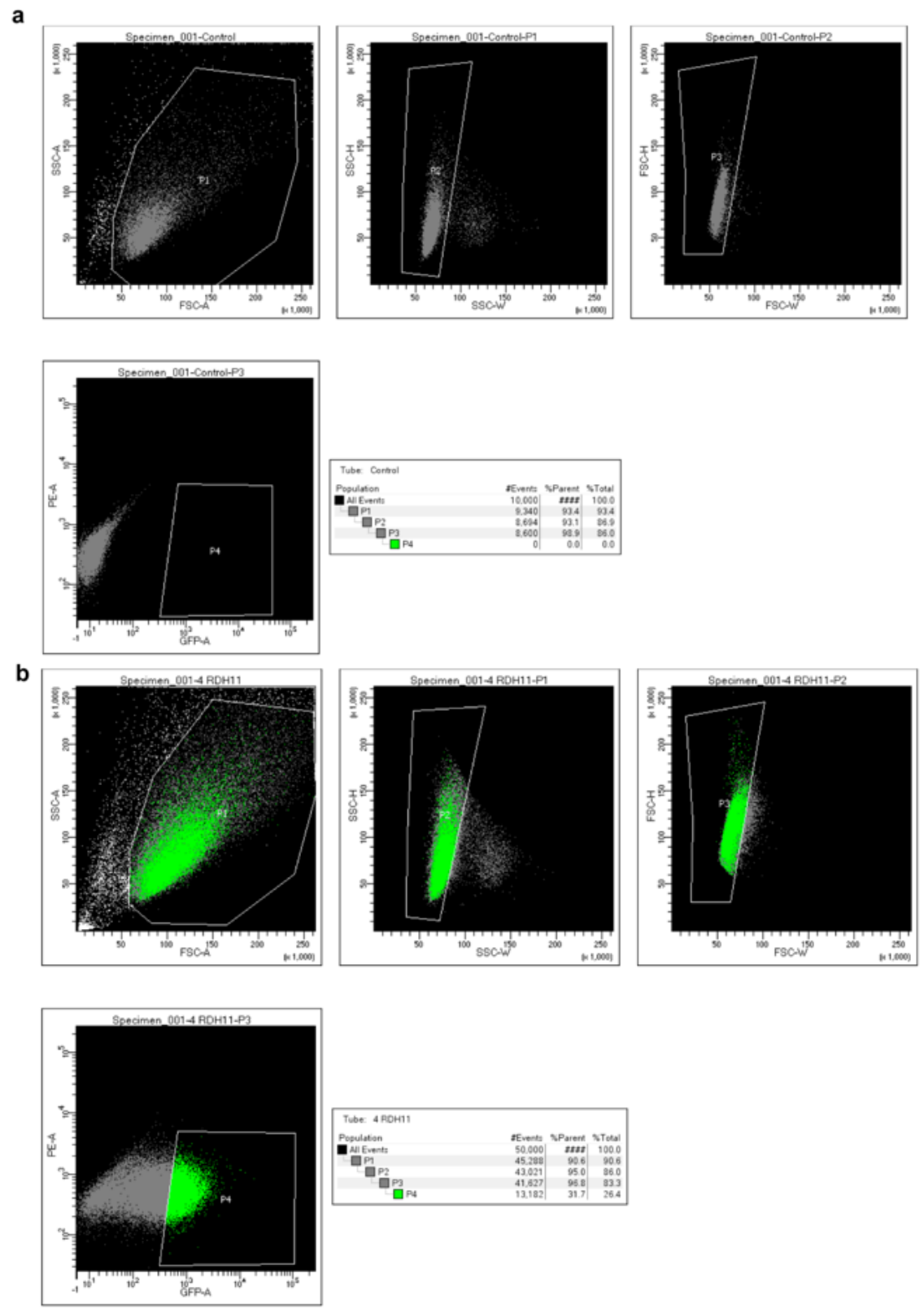

P1 FSC-A vs SSC-A — intact cells / debris exclusion.
P2 SSC-W vs SSC-H — singlet discrimination.
P3 FSC-W vs FSC-H — singlet discrimination.
P4 GFP-A vs PE-A sort gate.

Supplementary Figure 2. Representative FACS gating/sorting strategy for EGFP-positive stable cell lines. (a) GFP-negative control showing no events in the P4 (EGFP-positive) gate. (b) Representative sequential gating during FACS sorting of an EGFP-expressing RDH11 stable line. P1, intact cells (FSC-A versus SSC-A) excluding debris; P2 and P3, singlets identified by sequential singlet discrimination (SSC-W versus SSC-H, then FSC-W versus FSC-H); P4, EGFP-positive cells gated on GFP-A versus PE-A to exclude autofluorescent cells. Percentages indicate the fraction of the parent population. The same gating strategy was applied to all stable cell line sorting experiments performed on BD FACSAria (Aria 5/6), FACSymphony S6 and FACSDiscover S8 instruments. Flow cytometry was used solely to enrich fluorescent cells during stable line generation and was not used for experimental data acquisition.
